# Smoking Cessation and the Risk of Diabetes Mellitus and Impaired Fasting Glucose: Three-Year Outcomes after a Quit Attempt

**DOI:** 10.1371/journal.pone.0098278

**Published:** 2014-06-03

**Authors:** James H. Stein, Asha Asthana, Stevens S. Smith, Megan E. Piper, Wei-Yin Loh, Michael C. Fiore, Timothy B. Baker

**Affiliations:** Department of Medicine, University of Wisconsin School of Medicine and Public Health, Madison, Wisconsin, United States of America; The University of Auckland, New Zealand

## Abstract

Weight gain after smoking cessation may increase diabetes mellitus and impaired fasting glucose (IFG) risk. This study evaluated associations between smoking cessation and continued smoking with incident diabetes and IFG three years after a quit attempt. The 1504 smokers (58% female) were mean (standard deviation) 44.7 (11.1) years old and smoked 21.4 (8.9) cigarettes/day. Of 914 participants with year 3 data, the 238 abstainers had greater weight gain, increase in waist circumference, and increase in fasting glucose levels than the 676 continuing smokers (p≤0.008). In univariate analyses, Year 3 abstinence was associated with incident diabetes (OR = 2.60, 95% CI 1.44–4.67, p = .002; 4.3% absolute excess) and IFG (OR = 2.43, 95% CI 1.74–3.41, p<0.0001; 15.6% absolute excess). In multivariate analyses, incident diabetes was associated independently with older age (p = 0.0002), higher baseline body weight (p = 0.021), weight gain (p = 0.023), baseline smoking rate (p = 0.008), baseline IFG (p<0.0001), and baseline hemoglobin A1C (all p<0.0001). Smoking more at baseline predicted incident diabetes among eventual abstainers (p<0.0001); weighing more at baseline predicted incident diabetes among continuing smokers (p = 0.0004). Quitting smoking is associated with increased diabetes and IFG risk. Independent risk factors include older age, baseline body weight, baseline glycemic status, and heavier pre-quit smoking. These findings may help target smokers for interventions to prevent dysglycemia.

**Trial Registration:**

Clinicaltrials.gov NCT00332644

## Introduction

Quitting smoking is the most important behavioral change individuals can make to reduce their risk of cardiovascular disease (CVD), pulmonary disease, and cancer [1]. Cigarette smoking also is a risk factor for developing diabetes mellitus and impaired fasting glucose (IFG); therefore, smoking cessation should be associated with a decrease in the risk of type II diabetes mellitus [2]–[6]. However, smoking cessation can lead to weight gain, with various studies reporting average increases of 4–8 kg [7]–[9]. Reports of weight gain after smoking cessation raise concern that increases in adiposity could blunt or counter the proven health benefits of smoking cessation [2], [7], [9], [10] and might paradoxically increase risk for developing diabetes mellitus and IFG [2], [10], [11]. Some studies have suggested that smoking cessation leads to increased short-term risk of diabetes mellitus; however, most of these studies involved individuals from older cohorts who smoked more heavily and weighed less than contemporary smokers [2], [10]–[14]. Also, in observational cohort studies, abstainers may be motivated to quit as a result of disease exacerbation, so the association between quitting and disease outcomes may reflect worsening disease status prior to a quit attempt [15].

Although some studies suggest that quitting smoking may worsen glucose metabolism, there is a need to test this hypothesis in a modern cohort of smokers. Moreover, there is a clinical need to identify which smokers are most likely to develop diabetes mellitus and to identify possible mechanisms by which a smoking-related increase in abnormal glucose metabolism occurs. Such information could be useful for developing or targeting preventive interventions. To our knowledge, the effects of smoking and smoking cessation on incident diabetes mellitus and IFG have not been examined longitudinally in a *contemporary* cohort of active smokers. The purpose of this study was to examine the effects of smoking cessation and continued smoking on incident diabetes mellitus and IFG 3 years after a quit attempt in a large, contemporary cohort of active smokers who volunteered for a smoking cessation clinical trial. All participants were motivated to make a quit attempt and engaged in an attempt, reducing the likelihood of confounding between smoking cessation and pre-quit health status, including risk for IFG, diabetes mellitus, or CVD.

## Methods

The protocol for this study and supporting STROBE checklist are available as supporting information; see Checklist S1 and Protocol S1.

### Ethics Statement

This study was approved by the institutional review board of the University of Wisconsin School of Medicine and Public Health. All subjects provided free written informed consent. Research was conducted in accordance with the research principles in the Declaration of Helsinki.

### Study Participants and Design

This was a 3-year longitudinal, randomized, double-blind, placebo-controlled trial that evaluated the efficacy of five smoking cessation pharmacotherapies and the natural history of continued smoking and smoking cessation on CVD risk and other health outcomes (clinicaltrials.gov registration NCT00332644) [16]. The 1504 active smokers were randomized to 1 of 6 treatment conditions: nicotine lozenge, nicotine patch, bupropion SR, nicotine patch plus nicotine lozenge, bupropion SR plus nicotine lozenge, or placebo [16]. Major inclusion criteria included being ≥18 years old, smoking ≥10 cigarettes/day (cpd), expired carbon monoxide (CO) >9 ppm, and a stated motivation to quit. Major exclusion criteria were blood pressure (BP) >160/100 mmHg, recent myocardial infarction, heavy alcohol use, history of seizures or head injury, use of contraindicated medications, and current pregnancy or breastfeeding. Smoking cessation and other key study outcomes have been reported previously [16]–[18].

### Study Procedures

Participants were recruited from communities around Madison and Milwaukee, Wisconsin, from January, 2005 to June, 2007 (Figure S1). Baseline and year 3 (2008–2011) visits included measurements of anthropometric data, fasting laboratory testing, self-reported medication use, completion of validated questionnaires, and interviews. Fasting blood samples were obtained by venipuncture and refrigerated. Plasma aliquots were isolated by centrifugation and frozen at −70°.

### Definitions of Diabetes Mellitus and IFG

Participants were considered to have diabetes mellitus if they met one or more of the following criteria: use of antiglycemic medications, fasting plasma glucose ≥126 mg/dL, or HgbA_1_C ≥6.5% (48 mmol/mol). Particiants were considered to have IFG if they had a fasting glucose ≥100 mg/dL but did not have diabetes mellitus.

### Definitions of Year 3 Smoking Status

Year 3 abstainers were defined as participants who (i) attended the in-person year 3 follow-up, (ii) reported no smoking in the past 7 days at both the 6-month and year 3 follow-ups, (iii) had biochemical confirmation of abstinence (expired CO <10 ppm), and (iv) reported no smoking at the 30-month telephone follow-up (self-reported abstinence, not CO-verified). Continuing smokers were defined as those who attended the in-person year 3 follow-up and reported smoking in the past 7 days at either the 6-month or year 3 follow-ups, or both.

### Data Analysis

All analyses were performed with SAS software (Version 9.3, Cary, NC). Baseline differences between year 3 smokers and abstainers in demographic, smoking, and laboratory measures were tested with independent-groups t-tests for continuous variables and chi-square tests for categorical variables. We examined the main and interactive effects of year 3 smoking status and time (baseline, year 3) for selected measures of interest (weight, body-mass index, waist circumference, fasting glucose, HgbA_1_C, presence or absence of IFG, use of lipid-lowering medication, and use of anti-hypertensive medication). In these univariate mixed-effects models, time was modeled as a repeated (within-groups) effect and year 3 smoking status (abstinent or smoking) was modeled as a between-participants effect; the interaction between time and year 3 smoking status (time*group) also was included in these models. SAS PROC GLM was used to compute mixed-effects model analyses for continuous variables; analyses of categorical variables used SAS PROC GLIMMIX. PROC GLIMMIX computes a generalized linear mixed model that allows analysis of binary/categorical outcomes in longitudinal designs as well as specification of random effects, covariance structures, and other parameters in longitudinal models where outcomes are correlated. For these analyses, only the intercept in the models was specified as a random effect.

Multinomial logistic regression (MLR) was used in a series of models testing predictors of glycemic status at year 3. The MLR analysis sample excluded the 89 particpants with diabetes mellitus at baseline but those with baseline IFG were included so that IFG could be included as a risk factor for diabetes mellitus. In addition, because the best-fitting MLR model would be based on subjects with complete data, listwise deletion was used for these models (maximum N = 848). Three categorical outcomes served as the MLR dependent variable: no evidence of diabetes mellitus or IFG at year 3 (used as the reference group in the MLR models), IFG, and incident diabetes mellitus. One goal of the MLR analyses was to develop a best-fitting multivariate model that included the key orthogonal predictors of glycemic status at year 3. Candidate predictors for the best-fitting model were identified through a series of univariate MLR models. Predictors were retained for multivariate MLR models if the predictor was statistically significant in the univariate models (p<0.05) or was considered biologically important [19]. Next, we systematically tested a series of multivariate MLR models to identify key model effects (main effects and selected two-way interactions) for retention in a final, best-fitting model using bidirectional testing with inspection for collinearity.

## Results

### Participant Characteristics at Baseline

Participants included 252 (16.8% of the total sample) abstainers and 764 (50.8%) continuing smokers; 488 (32.5%) of the initial sample did not return for their year 3 visit. At baseline, 89 (6.0%) participants had type II diabetes mellitus and 358 (24.2%) had IFG. Table 1 describes the baseline characteristics of all participants by year 3 smoking and missingness status. At baseline, sex, weight, body-mass index, waist circumference fasting glucose, hemoglobin A_1_C, C-reactive protein, current smoking heaviness (cigarettes/day), smoking burden (pack-years), prevalence of IFG, and use of anti-hypertensive medications did not differ significantly among the 3 groups. At baseline, groups differed only by age, race, carbon monoxide level, use of lipid-lowering medications, and prevalence of diabetes mellitus at baseline (all p<0.05). Most between-group differences involved the group of participants who did not return for their year 3 visit.

**Table 1:** Baseline Participant Characteristics by Year 3 Smoking and Missingness Status.

Baseline Variable	N	Status at Year 3			Overall P-value*
		Abstinent (Group A)	Smoking (Group S)	Missing (Group M)	
Year 3 N (%)	1504	252 (16.8%)	764 (50.8%)	488 (32.5%)	-
Age (years)	1497	46.6 (11.5)	45.2 (10.6)	42.8 (11.2)	**<0.0001**
					A>M; S>M
Sex (% female)	1504	52.0%	59.8%	59.0%	0.084
Race	1504				
White		87.7%	80.0%	87.3%	**0.004**
Black		10.3%	16.5%	10.7%	A≠S
Other		2.0%	3.5%	2.1%	M≠S
Weight (kg)	1500	84.6 (21.2)	83.7 (20.2)	83.1 (20.5)	0.642
Body-mass index (kg/m^2^)	1500	29.0 (6.6)	29.2 (6.5)	28.7 (6.5)	0.423
Waist circumference (cm)	1481	96.8 (16.0)	96.0 (15.5)	95.1 (15.8)	0.339
Current smoking (cpd)	1500	20.5 (9.4)	21.4 (9.1)	22.0 (8.3)	0.102
Smoking burden (pack-years)	1499	29.1 (20.7)	29.9 (20.4)	28.9 (20.1)	0.634
Carbon monoxide (ppm)	1500	24.0 (11.5)	25.2 (12.6)	26.8 (12.8)	**0.011**
					M>A
C-reactive protein (log_10_ mg/dL)	1402	−0.40 (1.47)	−0.41 (1.47)	−0.45 (1.51)	0.892
Glucose (mg/dL)	1490	94.7 (15.8)	95.2 (17.6)	94.9 (18.4)	0.890
Hemoglobin A1C (%) [mmol/mol]	1483	5.57 (0.54) [37]	5.60 (0.67) [38]	5.52 (0.58) [37]	0.102
Diabetes mellitus (%)	1481	3.3	6.8	6.2	**0.042**
					S>A
Impaired fasting glucose (%)	1442	20.9	22.0	21.1	0.914
Use of lipid-lowering medication (%)	1492	6.0	5.0	1.4	**0.002**
					A>M S>M
Use of anti-hypertensive medication (%)	1492	5.2	5.5	3.7	0.332

All values are mean (standard deviation); *with post-hoc tests of significant group differences.

Compared to participants who did not have diabetes mellitus at baseline, those with diabetes mellitus were older, less likely to be white, had higher body weight, body-mass index, waist circumference, glucose and hemoglobin A_1_C values, and were more likely to be seen at the Milwaukee site and to be on lipid-lowering and anti-hypertensive medications (all p<0.001). Those who eventually abstained had a lower prevalence of diabetes mellitus at baseline (p<0.05). Participants with diabetes mellitus at baseline also had a greater smoking burden (pack-years, p<0.001).

### Associations of Abstinence and Continued Smoking at Year 3

Table 2 describes the baseline and year 3 levels of key parameters, by abstinence status with time*group interactions. Those who were abstinent at year 3 gained, on average, 4.3 kg more weight, had a 2.5 cm greater increase in waist circumference, and had a 4.6 mg/dL greater increase in fasting glucose levels than did continuing smokers (p<0.0001 for all). The effects of randomized treatment were not significant (see Appendix S2, Table 3B).

**Table 2:** Mixed-Effects Models Testing Selected Repeated Measures as a Function of Time and Year 3 Smoking Status.

Variable	Mean (%)						Mixed-Effects Models		
	Abstinent (Group A)			Smoking (Group S)			Model Effect	Test Statistic	P-value
	N	Baseline	Year 3	N	Baseline	Year 3			
Weight (kg)	238	83.5 (19.4)	90.0 (22.0)	676	82.7 (19.9)	84.9 (21.0)	Time Group **Interaction**	F(1,912) = 205.92	<0.0001
								F(1,912) = 3.99	0.0461
								F(1.912) = 51.19	**<0.0001**
Body-mass index (kg/m^2^)	238	28.6 (6.0)	30.9 (6.9)	676	28.8 (6.3)	29.4 (6.7)	Time Group **Interaction**	F(1,912) = 148.13	<0.0001
								F(1,912) = 1.86	0.1727
								F(1,912) = 43.20	**<0.0001**
Waist circumference (cm)	235	96.3 (15.3)	102.0 (16.3)	654	95.1 (15.1)	98.3 (16.1)	Time Group **Interaction**	F(1,887) = 194.65	<0.0001
								F(1,887) = 4.66	0.0311
								F(1,887) = 15.18	**<0.0001**
Fasting glucose (mg/dL)	232	93.0 (8.7)	98.4 (17.8)	654	92.5 (9.2)	93.3 (13.2)	Time Group **Interaction**	F(1,884) = 35.68	<0.0001
								F(1,884 = 12.99	0.0003
								F(1,884) = 19.76	**<0.0001**
Hemoglobin A_1_C (%) [mmol/mol]	232	5.5 (0.3)	5.7 (0.5)	656	5.5 (0.4)	5.6 (0.4)	Time Group **Interaction**	F(1,886) = 200.52	<0.0001
								F(1,886) = 5.76	0.0166
		[37]	[39]		[37]	[38]		F(1,886) = 5.56	**0.0186**

*All values are mean (standard deviation); note: 89 participants with diabetes mellitus at baseline are omitted from these analyses.*

**Table 3:** Best-Fitting Multivariate Multinomial Logistic Regression Model Predicting Year 3 Glucose Group (Normal, Impaired Fasting Glucose, Diabetes Mellitus).

	Year 3 IFG		Year 3 diabetes mellitus	
Main Effects	Wald χ^2^	p-value	Wald χ^2^	p-value
Year 3 smoking status (abstinent vs. smoking)	2.67	0.102	0.34	0.560
Age (years)	6.63	**0.010**	14.02	**0.0002**
Gender	0.40	0.526	3.70	.055
Race	0.03	0.869	1.26	0.262
Study site	36.77	**<0.0001**	1.05	0.306
Baseline weight (kg)	2.91	0.088	5.35	**0.021**
Δ Weight change, year 3 – baseline	0.27	0.602	5.17	**0.023**
Baseline smoking rate (cigarettes per day)	0.15	0.702	7.06	**0.008**
Baseline IFG group (glucose <100 mg/dL vs. glucose 100 mg/dL and ≤125 mg/dL)	41.96	**<0.0001**	16.30	**<0.0001**
Baseline hemoglobin A_1_C group (<5.7% vs. ≥5.7%)	1.44	0.230	19.52	**<0.0001**
**Interactions**				
Baseline weight * year 3 smoking status	5.32	**0.021**	8.25	**0.004**
Weight change * baseline cigarettes per day	1.88	0.170	0.93	0.335
Baseline cigarettes/day * year 3 smoking status	2.68	0.101	18.56	**<0.001**

*Reference group  =  normal Year 3 glycemic status.*

*IFG  =  impaired fasting glucose.*

### Incident diabetes mellitus and IFG as related to Weight Gain and Smoking Status

Development of diabetes mellitus and IFG was examined as a function of weight change, baseline HgbA_1_C and glucose levels, and smoking status at Year 3. Table 4 shows that for both abstainers and continuing smokers, elevated baseline HgbA_1_C (>5.7% [39 mmol/mol]) was associated with a heightened risk of incident diabetes mellitus, increasing risk from 1.3% to 11.4% in continuing smokers and from 0.3% to 20.3% amongst abstainers. Weight gain was less related to incident diabetes mellitus amongst continuing smokers than amongst abstainers. Table 4 shows the development of IFG status at Year 3 as a function of baseline glucose, weight gain and smoking status (those with baseline IFG are excluded). For both continuing smokers and abstinent participants, those who developed IFG gained more weight than those who did not develop IFG. The effect of smoking status is suggested by the incidence of IFG at year 3 among those who had baseline glucose from 100–125 at baseline: 52.9% of abstinent individuals had IFG at year 3, but only 19.3% of smokers did. These results suggest that the risk of developing diabetes mellitus and IFG appear to be related to baseline glycemic status and weight gain in both continuing smokers and those who became abstinent, but weight gain and both endpoints were greater in abstainers.

**Table 4:** Year 3 Weight Change, Diabetes Mellitus, and Impaired Fasting Glucose Status.

Variable	Baseline	Status at Year 3	Smoking at Year 3		Abstinent at Year 3	
			N	Δ Weight (kg)	N	Δ Weight (kg)
**Glucose**	≤90 mg/dL	No IFG	255	1.90 (7.36)	75	5.74 (8.47)
		Developed IFG	29	6.06 (6.64)	14	8.14 (10.63)
	90–100 mg/dL	No IFG	51	−0.94 (6.95)	13	6.82 (8.31)
		Developed IFG	55	1.80 (7.07)	22	6.95 (7.11)
	>100 mg/dL	No IFG	180	1.52 (6.13)	41	3.40 (11.15)
		Developed IFG	43	4.49 (6.65)	46	8.83 (7.19)
**Hemoglobin A_1_C**	≤5.7%	No	451	2.08 (7.32)	154	6.66 (8.19)
		diabetes				
	(39 mmol/mol)	Developed diabetes	6	8.55 (5.69)	5	11.20 (10.00)
	>5.7%	No	194	2.26 (8.62)	63	5.07 (10.29)
		diabetes				
	(39 mmol/mol)	Developed diabetes	25	1.68 (7.58)	16	9.08 (7.20)

*Values are mean (standard deviation); diabetes mellitus  =  diabetes mellitus; IFG  =  impaired fasting glucose. “No diabetes” includes Year 3 participants who met criteria for IFG.*

*Year 3 participants who met criteria for diabetes mellitus are excluded from the IFG analyses.*

### Univariate Multinomial Logistic Regression Models

Because development of diabetes mellitus and IFG could be related to several participant characteristics, we evaluated their associations with a broad range of variables. The first MLR models tested were univariate and included only the main effect for a given variable. Amongst the 21 variables tested, 17 were baseline measures, 3 were change variables (baseline to Year 3 change in weight, BMI, and HgbA1c), and one was Year 3 smoking status. Table 5 shows that quitting smoking significantly increased the likelihood of incident IFG and diabetes mellitus at year 3 relative to continuing to smoke. Rates of year 3 IFG were 19.7% for continuing smokers compared to 35.3% for abstainers (p<0.0001). Rates of year 3 diabetes mellitus were 4.7% in continuing smokers and 9.1% among abstainers. (p<0.01). With the exception of baseline CO and use of lipid-lowering medications, all of the measures significantly predicted either IFG, incident diabetes mellitus, or both, in the univariate models (Table 3).

**Table 5:** Univariate Predictors of Year 3 Glycemic Group Status.

Measure		Normal	IFG	DM
N		623	211	52
Year 3 Smoking Status (%)	Smoking	75.5	19.7	4.7
	Abstinent	55.6	35.3***	9.1**
Age (years)		44.2 (10.8)	47.0 (10.2)**	52.4 (9.2)***
Gender (%)	Female	74.2	20.2	5.6
	Male	64.8	29.0**	6.3
Race (%)	White	69.5	25.4	5.1
	Black	72.2	17.4	10.4
Study Site (%)	Milwaukee	78.6	15.1	6.4
	Madison	59.4	35.3***	5.2
Baseline weight (kg)		80.4 (18.9)	87.5 (19.3)***	94.6 (23.5)***
ΔWeight (kg), year 3– baseline		2.2 (7.6)	5.5 (7.6)***	5.7 (8.3)**
Baseline body mass index (kg/m^2^)		28.1 (6.0)	29.8 (6.0)**	32.4 (7.6)***
Δ Body-mass index,		0.7 (3.2)	1.9 (2.7)***	1.9 (2.9)**
year 3– baseline (kg/m^2^)				
Baseline waist circumference (cm)		92.8 (14.8)	100.1 (14.5)***	105.0 (14.1)***
Baseline smoking rate		20.67 (8.8)	21.50 (8.1)	24.2 (13.5)**
(cigarettes per day)				
Baseline pack-years of smoking (pack-years)		27.9 (19.6)	30.5 (17.7)	40.6 (27.5)***
Baseline expired carbon monoxide (ppm)		24.8 (12.3)	26.6 (12.7)	23.10 (11.2)
Baseline C-reactive protein		−0.55 (1.5)	−0.24 (1.5)**	−0.40 (1.5)
(log_10_ mg/dL)				
Baseline fasting glucose (mg/dL)		90.4 (8.1)	97.3 (9.3)***	100.8 (7.7)***
Baseline IFG (%)	Yes	41.2	43.9***	15.0***
	No	78.1	18.5	3.4
Baseline hemoglobin A1c (%)		5.4 (0.4)	5.6 (0.3)***	5.90 (0.4)***
Baseline hemoglobin A_1_C group (%)	≥5.7%	58.4%	27.5%**	14.1%***
	<5.7%	76.1%	22.0%	1.9%
Δ Hemoglobin A1c, year 3 – baseline (%)		0.12 (0.27)	0.19 (0.26)**	0.69 (0.68)***
Baseline use of lipid-lowering medication (%)	Yes	59.5%	28.6%	11.9%
	No	70.7%	23.7%	5.6%
Baseline use of hypertension medication (%)	Yes	53.5%	34.9%*	11.6%
	No	71.1%	23.3%	5.6%

*IFG  =  impaired fasting glucose; DM  =  diabetes mellitus.*

*Asterisks indicate significance levels for the variables in the prediction of either Year 3 IFG or diabetes mellitus by univariate multinomial logistic regressions; the group consisting of participants with normal glycemic status was the reference condition for the IFG and DM groups:* *  = p<0.05; **  = p<0.01; ***  = p<0.0001.

### Best-Fitting Multinomial Logistic Regression Model

A best-fitting multivariable MLR model was built from successive models to identify the variables with significant, independent relationships with year 3 glycemic status. This model used predictor variables that were associated with glycemic status in the univariate tests and that were of substantive interest (*i.e.*, year 3 smoking status, gender, study site, age, race, baseline weight, weight change, baseline HgbA_1_C, baseline smoking heaviness [cigarettes/day], and baseline IFG status [Table 3]).

For incident diabetes mellitus, significant effects were identified for age (p = 0.0002), baseline body weight (p = 0.021), baseline HgbA_1_C (p<0.0001), and baseline IFG group (p<0.0001); year 3 smoking status interacted with baseline weight and smoking heaviness (p = 0.004). The baseline weight by year 3 smoking status interaction was observed because baseline body weight differed between those who developed diabetes mellitus and those who did not, but only for participants who continued to smoke. Among continuing smokers, those who developed incident diabetes mellitus weighed 99.9 (26.0) kg at baseline, whereas those without incident diabetes mellitus weighed 82.1 (19.2) kg (p<0.0001). Incident diabetes mellitus likelihood was less related to baseline body weight among eventual abstainers. At baseline, abstainers who developed diabetes mellitus and those who did not weighed 86.8 (16.8) kg and 83.2 (19.6) kg, respectively (p = 0.422). The baseline smoking heaviness by year 3 smoking status interaction was observed because baseline smoking heaviness was related to diabetes mellitus development at year 3, but only for participants who were abstinent. Abstainers who developed diabetes mellitus smoked 28.3 (16.8) cigarettes/day; those who did not develop diabetes mellitus smoked 19.1 (7.7) cigarettes/day (p<0.0001). There was no association between baseline smoking heaviness and incident diabetes mellitus risk among those who continued to smoke. Thus, abstinence had an especially strong effect for incident diabetes mellitus among those who smoked most heavily; however, smoking heaviness did not increase risk of diabetes mellitus significantly among those who continued to smoke.

IFG was included in the overall MLR model since it was important to the prediction of incident diabetes mellitus. To ensure that its entry was not masking other important predictors, the model was run without IFG; this did not meaningfully change the pattern of relations of the other predictors with Year 3 IFG. As also shown in Table 3, for IFG, significant effects were identified for age, study site, and IFG at baseline. In addition, a significant interaction between baseline weight and year 3 smoking status was identified for Year 3 IFG (p = 0.021).

## Discussion

Successful smoking cessation is associated with weight gain and a clinically relevant increase in the risk of developing diabetes mellitus or IFG; however, the relationships between smoking cessation, weight gain, and development of diabetes mellitus and IFG are complex. Several variables are associated with an increased risk for IFG and diabetes mellitus over 3 years of follow-up (Table 3), but some factors are especially robustly associated with these outcomes (Table 3). In particular, risk for developing diabetes mellitus is independently related to age, baseline body weight, baseline IFG, and having a HgbA_1_C >5.7% (39 mmol/mol) at baseline. IFG is especially associated with age, baseline body weight, and predictably, IFG at baseline. While quitting smoking was strongly associated with both IFG and diabetes mellitus in univariate tests, the best-fitting model shows that this relationship can be best understood in terms of its significant interactions with baseline body weight and smoking heaviness.

Weight gain after smoking cessation is of considerable importance to patients [20], [21]. Weight gain is associated with increased risk for incident IFG and diabetes mellitus. In our study, weight gain was associated with incident diabetes mellitus but not IFG in our best-fitting model (the weight gain p value for incident diabetes mellitus was 0.023).

One purpose of this research was to gather data that would help a clinician evaluate whether a patient who smokes might be especially likely to develop diabetes mellitus after quitting. Based upon both the univariate and best-fitting models, the variables that most strongly predict development of diabetes mellitus are being older, higher body weight prior to quitting smoking, and having elevated levels of glucose and HgbA_1_C at baseline. If a clinician were seeing a smoker who has quit, the number of pre-cessation cigarettes smoked/day also would convey information on future risk of incident diabetes mellitus. However, a series of decision tree models [22] run in this sample (see Appendix S1) showed that the best single significant predictor of diabetes mellitus at year 3 was baseline HgbA_1_C, which had a cut-score of about 5.7%. This single predictor separated non-diabetic patients who smoked into groups that 3 years later had a 1–2% risk for incident diabetes mellitus versus a 13–15% risk (across the combined sample of those who quit and those who didn't). For IFG at year 3, baseline glucose was the best predictor. Only 8% of individuals with glucose ≤87 mg/dL developed IFG compared to 68% with higher glucose levels. In these models, abstinence status and baseline weight or waist circumference also seemed to influence risk of IFG among those with higher glucose levels at baseline (Appendix S1).

Our findings are consistent with a model in which smokers develop diabetes mellitus based upon known risk factors such as baseline adiposity, IFG, and HgbA_1_C, but that quitting vs. continuing smoking also influences risk. Specifically, baseline body weight was more of a determinant of diabetes mellitus development in continuing smokers than in abstainers. Conversely, smoking heaviness increased diabetes mellitus risk, but this risk was apparent only after a smoker became abstinent. Other studies have linked quitting-associated diabetes mellitus with heavier or longer pre-cessation smoking and higher initial body weight among quitters [14]. Quitting may unmask smoking-related pancreatic β-cell damage or dysfunction [14]; however, the effects of smoking on β-cell function are not clear [23], [24]. It is also possible that genetic factors that permit heavy smoking such as certain chromosome 15 haplotypes [25], [26], also confer increased risk for diabetes mellitus development. These haplotypes have been associated with health outcomes such as lung cancer and chronic obstructive pulmonary disease [27]–[29], but not necessarily independent of their effects on smoking heaviness. In summary, the risk of diabetes mellitus in smokers is most strongly related to age, glycemic status, initial bodyweight, and quitting versus continuing smoking, with the latter modulating the impact of other risk factors (e.g., smoking heaviness).

The magnitudes of the excess risks of incident diabetes mellitus and IFG we identified in our study are greater than reported previously, possibly because our participants were contemporary smokers with higher body-mass indexes than in historical cohorts [30], [31]. Also, our sample included a large percentage of women and was socioeconomically diverse, due in part to the Milwaukee recruiting site, where participants were more likely to be non-white and have low incomes. Indeed, site was a significant predictor of developing IFG, with IFG risk being greater in those from Madison. We have no ready explanation for this observation other than the hypothesis that smoking is less normative in Madison than in Milwaukee, and thereby perhaps selects out a more deviant population.

While smoking cessation was related to increased incident diabetes mellitus, particularly amongst the heaviest smokers, it is vital to recognize that smoking cessation has tremendous health benefits despite its relation with incident diabetes mellitus and IFG. For instance, in this same cohort, we previously demonstrated that despite weight gain, smoking cessation leads to improvements in lipids, lipoproteins, and endothelial function, each of which are established markers of CVD risk [17], [18]. Recently, longitudinal analyses from the Framingham Heart Study and Women's Health Initiative showed that weight gain after a quit attempt did not significantly attenuate the CVD risk reduction after quitting smoking [12], [13]. However, the participants described in those reports had lower body-mass indexes than those in our study. Our participants are more reflective of contemporary smokers; however, nothing in this report alters the fact that quitting smoking is the most important action that most smokers can take to protect and improve their overall health [32], [33].

Identifying baseline predictors of diabetes mellitus and IFG prior to a quit attempt is important because of the potential to target higher risk patients for interventions that may prevent diabetes mellitus development among at-risk individuals [34]–[36]. Unfortunately, the efficacy of lifestyle interventions in conjunction with a quit smoking attempt is modest [37]–[39], underscoring the critical need to develop interventions and better diabetes mellitus risk prediction tools to identify smokers that might benefit most from such interventions. A recent systematic review found that combination cessation pharmacotherapies produced less cessation-related weight gain in the short term, but the best results for long-term weight loss involved behavioral interventions [37]. That review did not highlight specific behavioral interventions or predictors of response.

### Limitations

All participants were enrolled in a smoking cessation clinical trial, so there were no non-smoking controls. Continuous smokers may have become ill and those that developed diabetes mellitus may have been too ill to attend the follow-up visit. However, clinical trial participation with an intention to quit smoking reduces the risk of confounding by health status. As is commonly seen in smoking cessation trials [40], [41], our study had a nontrivial drop out rate (32.5%) so we cannot exclude bias based on continuing participation; however, participants that do not return typlically are continuing smokers rather than successful abstainers. Because we did not serially measure glucose and insulin levels, we cannot describe the time course or pathophysiology of our observations. The long-term cardiovascular effects of smoking and risk of diabetes mellitus and/or IFG were not assessed and our particiapants were moderate to heavy smokers. We also did not assess second-hand smoke exposure in a rigorous way, though the effect is not likely to be large. Also, use of antiglycemic medications can be used for other conditions, so some misclassifcation may have occurred, though this likely would have created a null bias. Despite these limitations, this study possesses several strengths relative to some other studies evaluating smoking and diabetes mellitus risk [14] including biomarker assessment of diabetes mellitus and IFG status, measurement of body weight (vs. self-report), and biochemical verification of cessation status.

## Conclusions

Smoking cessation is associated with increased diabetes mellitus and IFG risk. The most important predictors of these post-cessation outcomes are age, baseline glycemic status, and initial body weight. Smoking more at baseline predicted diabetes mellitus among eventual abstainers. Weighing more at baseline predicted incident diabetes mellitus among continuing smokers. Weight gain after a quit attempt was related to IFG and diabetes mellitus, but not independent of other risk factors. These findings may help target smokers for interventions to prevent diabetes mellitus and IFG, and suggest mechanisms for the development of dysglycemia among successful abstainers and continuing smokers.

## Supporting Information

Figure S1 **CONSORT Flow Diagram.**
(PDF)

Appendix S1 **Decision Tree Analyses.**
(PDF)

Appendix S2 **Summary of** **Table 3** **Re-Analysis -** **Tables 3A and 3B** **.**
(PDF)

Checklist S1 **STROBE Checklist.**
(PDF)

Protocol S1 **Research Grant and Consent Form.**
(PDF)
