# Supplementary figures and images for: Smoking Cessation and the Risk of Diabetes Mellitus and Impaired Fasting Glucose: Three-Year Outcomes after a Quit Attempt
Source: PLoS One. 2014 Jun 3;9(6):e98278. doi: 10.1371/journal.pone.0098278 (PMC4043674; doi:10.1371/journal.pone.0098278)

Figure 1. CONSORT Flow Diagram

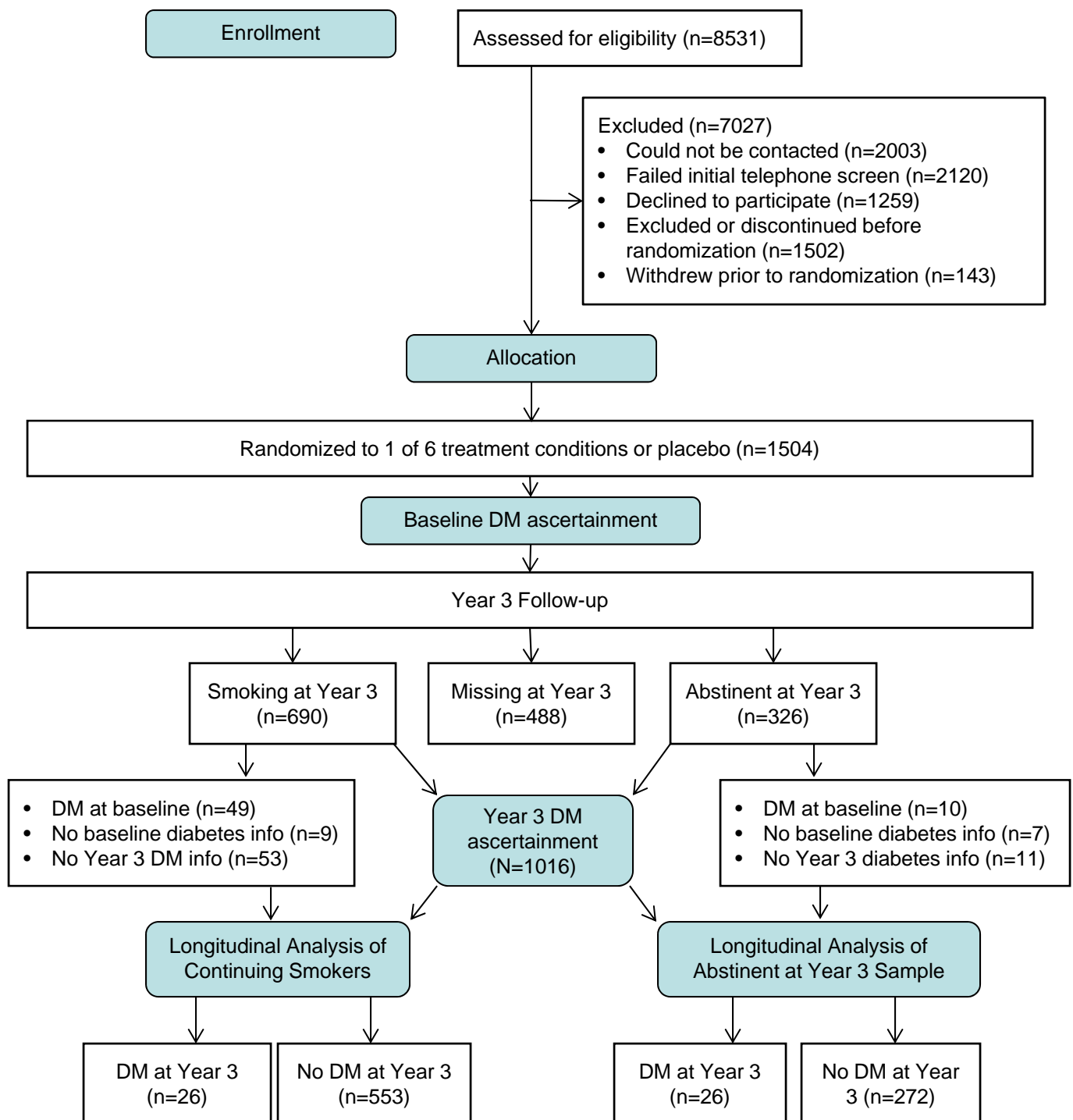

DM = diabetes mellitus

Supplement: Figure S1 — CONSORT Flow Diagram. (PDF) [file pone.0098278.s001.pdf]
