# Supplementary material for: Smoking Cessation and the Risk of Diabetes Mellitus and Impaired Fasting Glucose: Three-Year Outcomes after a Quit Attempt
Source: PLoS One. 2014 Jun 3;9(6):e98278. doi: 10.1371/journal.pone.0098278 (PMC4043674; doi:10.1371/journal.pone.0098278)
Supplement: Appendix S1 — Decision Tree Analyses. (PDF) [file pone.0098278.s002.pdf]

SUPPLEMENTAL MATERIAL

Appendix 1. Decision Tree Analyses

To better understand the predictors of risk for diabetes mellitus and impaired fasting glucose over the course of a quit attempt, decision tree analysis was performed using the GUIDE algorithm.[1] This algorithm iteratively divided subjects into 2 groups based on their maximal statistically significant separation. Each node reflected the specific predictor value that optimally split the subgroups with the group below and to the left of the node satisfying the splitting criterion. The group below and to the right are therefore the remaining subjects; below the nodes are the percent of subjects positive for the outcome and the tree stops splitting when a resulting subgroup is <20 subjects, after which it is pruned so that its estimated error rate is minimized. By decision tree analysis, the best baseline predictor for developing diabetes mellitus was having a baseline HgbA1C >5.7% (39 mmol/mol). As shown in Appendix Figure 1, 15% of subjects who had a HgbA1C >5.7% 39 mmol/mol) at baseline developed diabetes mellitus, while only 2.0% of subjects who had a HgbA1C ≤5.7% (39 mmol/mol) at baseline developed diabetes mellitus.

For new impaired fasting glucose (Appendix Figure 2), the best predictor of developing impaired fasting glucose was having a baseline glucose >87 mg/dL. For subjects with a baseline glucose ≤87 mg/dL, only 8.0% developed impaired fasting glucose, while 43% of subjects who had a baseline glucose >87 mg/dL and were abstinent at year 3 developed impaired fasting glucose. Continuing smokers were at a great risk of developing impaired fasting glucose if their waist circumference was >92 cm at baseline (25%).

Appendix Figure 1. Predictors of New Diabetes Mellitus by Year 3

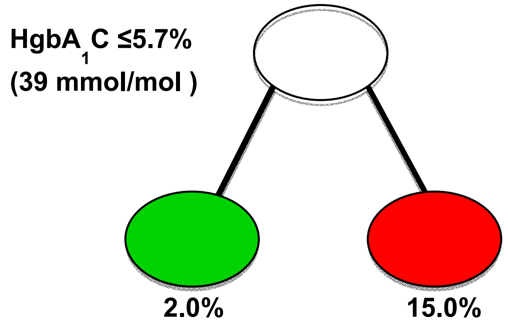

Appendix Figure 2. Predictors of New IFG by Year 3

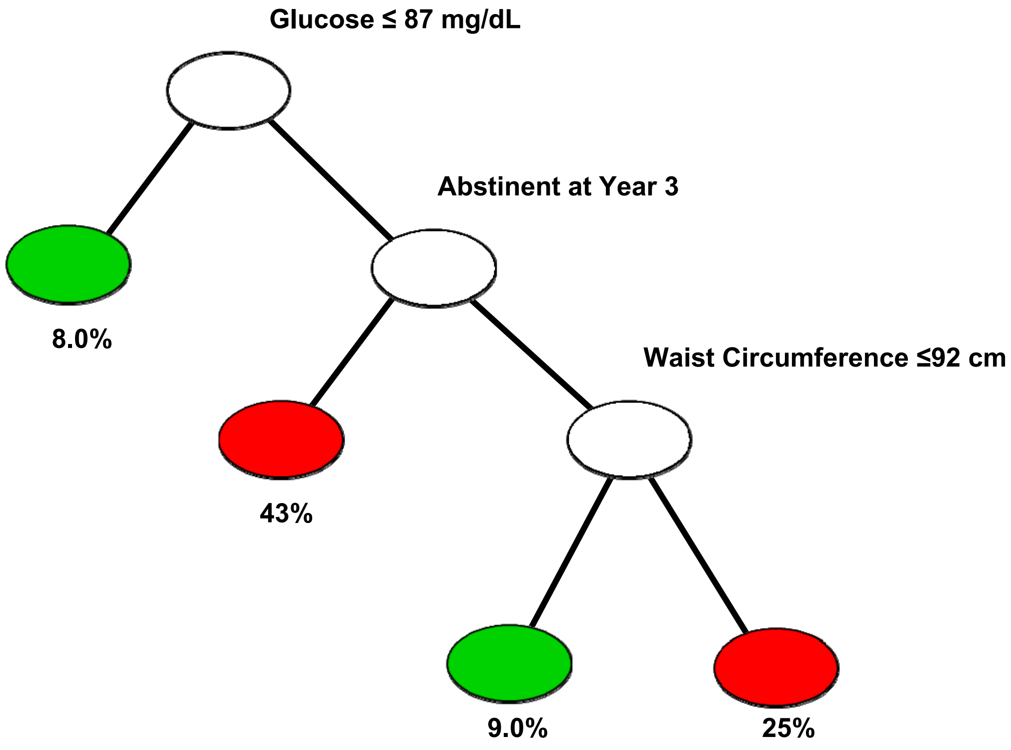

Reference

1. Daniel M, Cargo MD. (2004) Association between smoking, insulin resistance and beta-cell function in a North-western First Nation. Diabet Med 21: 188-193.
