# Supplementary material for: Smoking Cessation and the Risk of Diabetes Mellitus and Impaired Fasting Glucose: Three-Year Outcomes after a Quit Attempt
Source: PLoS One. 2014 Jun 3;9(6):e98278. doi: 10.1371/journal.pone.0098278 (PMC4043674; doi:10.1371/journal.pone.0098278)
Supplement: Appendix S2 — Summary of Table 3 Re-Analysis - Tables 3A and 3B . (PDF) [file pone.0098278.s003.pdf]

New Table 3A. Best-Fitting Multivariate Multinomial Logistic Regression Model Predicting Year 3 Glucose Group (Normal, Impaired Fasting Glucose, Diabetes Mellitus)

THIS ANALYSIS: uses the new version of Year 3 Smoking Status [if smoking at either 6-months or 3 years (or both), then the variable was coded as smoking]. Differences in the significance of effects (original vs new results) are highlighted in BLUE.

New Table 3A	ORIGINAL ANALYSIS				NEW ANALYSIS (Uses new Year 3 Smoking Status Variable)			
	Year 3 IFG		Year 3 diabetes mellitus		Year 3 IFG		Year 3 diabetes mellitus	
**Main Effects or Interactions**	**Wald χ^2**	**p-value**	**Wald χ^2**	**p-value**	**Wald χ^2**	**p-value**	**Wald χ^2**	**p-value**
Year 3 smoking status (abstinent vs. smoking)	0.75	0.387	0.73	0.392	2.67	0.102	0.34	0.560
Age (years)	9.46	**0.011**	14.53	**0.0001**	6.63	**0.010**	14.02	**0.0002**
Gender	0.85	0.357	2.29	0.131	0.40	0.526	3.70	.055
Race	0.03	0.853	1.64	0.201	0.03	0.869	1.26	0.262
Study site	37.45	**<0.0001**	1.31	0.253	36.77	**<0.0001**	1.05	0.306
Baseline weight (kg)	6.35	**0.012**	14.31	**0.0002**	2.91	0.088	5.35	**0.021**
Δ Weight change, year 3 – baseline	0.44	0.506	3.19	0.074	0.27	0.602	5.17	**0.023**
Baseline smoking rate (cigarettes per day)	0.24	0.627	1.24	0.265	0.15	0.702	7.06	**0.008**
Baseline IFG group (glucose < 100 mg/dL vs. glucose ≥ 100 mg/dL and ≤ 125 mg/dL)	40.85	**<0.0001**	15.95	**<0.0001**	41.96	**<0.0001**	16.30	**<0.0001**
Baseline hemoglobin A_1C group (< 5.7% vs. ≥ 5.7%)	1.70	0.193	16.04	**<0.0001**	1.44	0.230	19.52	**<0.0001**

New Table 3A	ORIGINAL ANALYSIS				NEW ANALYSIS (Uses new Year 3 Smoking Status Variable)			
	Year 3 IFG		Year 3 diabetes mellitus		Year 3 IFG		Year 3 diabetes mellitus	
**Main Effects or Interactions**	**Wald χ^2**	**p-value**	**Wald χ^2**	**p-value**	**Wald χ^2**	**p-value**	**Wald χ^2**	**p-value**
**Interactions:**								
Baseline weight * year 3 smoking status	1.41	0.235	6.10	**0.014**	5.32	**0.021**	8.25	**0.004**
Weight change * baseline cigarettes per day	1.46	0.228	0.09	0.755	1.88	0.170	0.93	0.335
Baseline cigarettes/day * year 3 smoking status	1.53	0.217	9.07	**0.003**	2.68	0.101	18.56	**<0.001**

Reference group = normal Year 3 glycemic status; IFG = impaired fasting glucose

NOTE: In the new analysis, "Year 3 smoking status" was coded so that "abstinent" required CO-confirmed abstinence at both 6 months and at Year 3; if smoking at either 6-months or 3 years (or both), then the variable was coded as smoking.

New Table 3B. Best-Fitting Multivariate Multinomial Logistic Regression Model Predicting Year 3 Glucose Group (Normal, Impaired Fasting Glucose, Diabetes Mellitus)

THIS NEW ANALYSIS: Uses the New Version of Year 3 Smoking Status [if smoking at either 6-months or 3 years (or both), then the variable was coded as smoking] PLUS it adds treatment (with Dummy-Coded Variables). Differences in the significance of effects (original vs new results) are highlighted in BLUE.

New Table 3B	ORIGINAL ANALYSIS				NEW ANALYSIS (Adds Treatment Variables and Uses New Year 3 Smoking Status Variable)			
	Year 3 IFG		Year 3 diabetes mellitus		Year 3 IFG		Year 3 diabetes mellitus	
**Main Effects or Interactions**	**Wald χ^2**	**p-value**	**Wald χ^2**	**p-value**	**Wald χ^2**	**p-value**	**Wald χ^2**	**p-value**
Year 3 smoking status (abstinent vs. smoking)	0.75	0.387	0.73	0.392	2.68	0.102	0.028	0.868
Age (years)	9.46	**0.011**	14.53	**0.0001**	6.83	**0.009**	13.83	**0.0002**
Gender	0.85	0.357	2.29	0.131	0.52	0.471	4.22	**0.040**
Race	0.03	0.853	1.64	0.201	0.018	0.893	1.42	0.233
Study site	37.45	**<0.0001**	1.31	0.253	38.67	**<0.0001**	1.12	0.290
Baseline weight (kg)	6.35	**0.012**	14.31	**0.0002**	3.24	**0.072**	4.74	**0.0294**
Δ Weight change, year 3 – baseline	0.44	0.506	3.19	0.074	0.29	0.590	5.43	**0.0198**
Baseline smoking rate (cigarettes per day)	0.24	0.627	1.24	0.265	0.16	0.692	6.82	**0.009**
Baseline IFG group (glucose < 100 mg/dL vs. glucose ≥ 100 mg/dL and ≤ 125 mg/dL)	40.85	**<0.0001**	15.95	**<0.0001**	41.94	**<0.0001**	16.20	**<0.0001**

New Table 3B	ORIGINAL ANALYSIS				NEW ANALYSIS (Adds Treatment Variables and Uses New Year 3 Smoking Status Variable)			
	Year 3 IFG		Year 3 diabetes mellitus		Year 3 IFG		Year 3 diabetes mellitus	
**Main Effects or Interactions**	**Wald χ^2**	**p-value**	**Wald χ^2**	**p-value**	**Wald χ^2**	**p-value**	**Wald χ^2**	**p-value**
Baseline hemoglobin A_1C group (< 5.7% vs. ≥ 5.7%)	1.70	0.193	16.04	**<0.0001**	1.59	0.208	20.90	**<0.0001**
Bupropion	-	-	-	-	0.11	.740	0.389	0.533
Lozenge	-	-	-	-	0.02	.895	1.06	0.304
Patch	-	-	-	-	0.95	0.330	0.0003	0.987
Bupropion + Lozenge	-	-	-	-	0.19	0.665	1.73	0.189
Patch + Lozenge	-	-	-	-	1.02	0.312	2.10	0.148
**Interactions:**								
Baseline weight * year 3 smoking status	1.41	0.235	6.10	**0.014**	5.12	**0.024**	8.90	**0.0029**
Weight change * baseline cigarettes per day	1.46	0.228	0.09	0.755	1.87	0.171	1.28	0.258
Baseline cigarettes/day * year 3 smoking status	1.53	0.217	9.07	**0.003**	2.45	0.117	17.48	**<0.0001**
Bupropion * year 3 smoking status	-	-	-	-	0.59	0.443	0.16	0.691
Lozenge * year 3 smoking status	-	-	-	-	1.10	0.294	0.05	0.825
Patch * year 3 smoking status	-	-	-	-	0.15	0.701	1.87	0.171

New Table 3B	ORIGINAL ANALYSIS				NEW ANALYSIS (Adds Treatment Variables and Uses New Year 3 Smoking Status Variable)			
	Year 3 IFG		Year 3 diabetes mellitus		Year 3 IFG		Year 3 diabetes mellitus	
**Main Effects or Interactions**	**Wald χ^2**	**p-value**	**Wald χ^2**	**p-value**	**Wald χ^2**	**p-value**	**Wald χ^2**	**p-value**
Bupropion + Lozenge * year 3 smoking status	-	-	-	-	0.12	0.724	0.74	0.389
Patch + Lozenge * year 3 smoking status	-	-	-	-	0.41	0.522	0.68	0.411

Reference group = normal Year 3 glycemic status; IFG = impaired fasting glucose

NOTE: In the new analysis, "Year 3 smoking status" was coded so that "abstinent" required CO-confirmed abstinence at both 6 months and at Year 3; if smoking at either 6-months or 3 years (or both), then the variable was coded as smoking.
