# Supplementary material for: Smoking Cessation and the Risk of Diabetes Mellitus and Impaired Fasting Glucose: Three-Year Outcomes after a Quit Attempt
Source: PLoS One. 2014 Jun 3;9(6):e98278. doi: 10.1371/journal.pone.0098278 (PMC4043674; doi:10.1371/journal.pone.0098278)
Supplement: Checklist S1 — STROBE Checklist. (PDF) [file pone.0098278.s004.pdf]

STROBE Statement—Checklist of items that should be included in reports of ***cohort studies***

	Item No.	Recommendation	Page No.
**Title and abstract**	1	(*a*) Indicate the study's design with a commonly used term in the title or the abstract	Abstract
		(*b*) Provide in the abstract an informative and balanced summary of what was done and what was found	Abstract
**Introduction**			
Background/rationale	2	Explain the scientific background and rationale for the investigation being reported	Introduction
Objectives	3	State specific objectives, including any prespecified hypotheses	Introduction
**Methods**			
Study design	4	Present key elements of study design early in the paper	Methods – Study Participants & Design
Setting	5	Describe the setting, locations, and relevant dates, including periods of recruitment, exposure, follow-up, and data collection	Methods – Study Procedures
Participants	6	(*a*) Give the eligibility criteria, and the sources and methods of selection of participants. Describe methods of follow-up	Methods
		(*b*) For matched studies, give matching criteria and number of exposed and unexposed	NA
Variables	7	Clearly define all outcomes, exposures, predictors, potential confounders, and effect modifiers. Give diagnostic criteria, if applicable	Methods - Definitions
Data sources/ measurement	8*	For each variable of interest, give sources of data and details of methods of assessment (measurement). Describe comparability of assessment methods if there is more than one group	Methods – Data Analysis, Appendix
Bias	9	Describe any efforts to address potential sources of bias	Methods – Data Analysis, Conclusions, Appendix
Study size	10	Explain how the study size was arrived at	Methods, Study Participants & Design
Quantitative variables	11	Explain how quantitative variables were handled in the analyses. If applicable, describe which groupings were chosen and why	Methods – Data Analysis, Appendix
Statistical methods	12	(*a*) Describe all statistical methods, including those used to control for confounding	Methods – Data Analysis
		(*b*) Describe any methods used to examine subgroups and interactions	Methods – Data Analysis, Appendix
		(*c*) Explain how missing data were addressed	Methods – Data Analysis
		(*d*) If applicable, explain how loss to follow-up was addressed	Methods – Data Analysis
		(*e*) Describe any sensitivity analyses	NA
**Results**			
Participants	13*	(a) Report numbers of individuals at each stage of study—eg	Results –

		numbers potentially eligible, examined for eligibility, confirmed eligible, included in the study, completing follow-up, and analysed	Participant Characteristics at Baseline
		(b) Give reasons for non-participation at each stage	Results – Participant Characteristics at Baseline
		(c) Consider use of a flow diagram	NA
Descriptive data	14*	(a) Give characteristics of study participants (eg demographic, clinical, social) and information on exposures and potential confounders	Results – Participant Characteristics at Baseline, Table 1
		(b) Indicate number of participants with missing data for each variable of interest	Results – Participant Characteristics at Baseline, Table 1
		(c) Summarise follow-up time (eg, average and total amount)	Results – Associations of Abstinence and Continued Smoking at Year 3 and Incident diabetes mellitus as related to Weight Gain and Smoking Status, Table 1
Outcome data	15*	Report numbers of outcome events or summary measures over time	Results, Tables
Main results	16	(*a*) Give unadjusted estimates and, if applicable, confounder-adjusted estimates and their precision (eg, 95% confidence interval). Make clear which confounders were adjusted for and why they were included	Results, Tables
		(*b*) Report category boundaries when continuous variables were categorized	Results, Tables
		(*c*) If relevant, consider translating estimates of relative risk into absolute risk for a meaningful time period	Results
Other analyses	17	Report other analyses done—eg analyses of subgroups and interactions, and sensitivity analyses	Results, Tables, Appendix
**Discussion**			
Key results	18	Summarise key results with reference to study objectives	Discussion
Limitations	19	Discuss limitations of the study, taking into account sources of potential bias or imprecision. Discuss both direction and magnitude of any potential bias	Discussion – Limitations
Interpretation	20	Give a cautious overall interpretation of results considering objectives, limitations, multiplicity of analyses, results from similar studies, and other relevant evidence	Discussion

Generalisability	21	Discuss the generalisability (external validity) of the study results	Discussion – Limitations
**Other information**			
Funding	22	Give the source of funding and the role of the funders for the present study and, if applicable, for the original study on which the present article is based	Online Editor Manager

*Give information separately for exposed and unexposed groups.

Note: An Explanation and Elaboration article discusses each checklist item and gives methodological background and published examples of transparent reporting. The STROBE checklist is best used in conjunction with this article (freely available on the Web sites of PLoS Medicine at http://www.plosmedicine.org/, Annals of Internal Medicine at http://www.annals.org/, and Epidemiology at http://www.epidem.com/). Information on the STROBE Initiative is available at http://www.strobe-statement.org.
