# Supplementary material for: Smoking Cessation and the Risk of Diabetes Mellitus and Impaired Fasting Glucose: Three-Year Outcomes after a Quit Attempt
Source: PLoS One. 2014 Jun 3;9(6):e98278. doi: 10.1371/journal.pone.0098278 (PMC4043674; doi:10.1371/journal.pone.0098278)
Supplement: Protocol S1 — Research Grant and Consent Form. (PDF) [file pone.0098278.s005.pdf]

PHARMACOTHERAPIES: EFFICACY, MECHANISMS, AND ALGORITHMS

A. Specific Aims

There is substantial evidence that smoking cessation pharmacotherapies are effective. Numerous studies and meta-analyses show that medications significantly boost long-term cessation rates, and they do so across types and levels of adjuvant interventions.[1-4] In addition, cessation pharmacotherapies are popular with smokers[5] and their use appears to be increasing.[6] Finally, there is convincing evidence that cessation pharmacotherapies are cost-effective relative to many other sorts of preventive interventions.[7,8]

While cessation pharmacotherapies are cost-effective and widely used, there is a great need to improve their efficacy and discover strategies for their optimal use. Even with pharmacotherapies, the majority of smokers trying to quit still relapse.[2] One reason that we cannot use or apply cessation strategies more effectively is that we have little direct knowledge of how treatments compare to one another in general, or in particular populations (e.g., in women or the highly tobacco dependent). This gap in knowledge arises due to a lack of “head-to-head” comparisons of different pharmacotherapies in the same study. Without such head-to-head trials, researchers and clinicians are limited to estimating relative efficacies across studies. However, cessation studies differ in myriad respects: intensity of clinical contact, type of outcome assessed, duration of pharmacotherapy, recruitment/inclusion criteria, assessment burden, and so forth. As such, inter-study comparisons are risky and fraught with error such that meta-analyses may yield conclusions that conflict markedly with large scale focused studies.[9] In part, these limitations have forced clinical guidelines to recommend equally all FDA-approved pharmacotherapies and not distinguish among them.[2,10] On the other hand, head-to-head (intra-study) comparisons are quite capable of distinguishing among the efficacies of different pharmacotherapies or pharmacotherapy combinations.[11,12]

We need to determine not only overall levels of efficacy, but also efficacy in particular populations. This would allow us to match smokers optimally with treatments. Evidence now suggests that some types of smokers particularly benefit from particular treatments. For instance, evidence is mounting that women are little helped by standard NRT interventions, but are helped substantially by bupropion.[13,14] Direct comparisons of different treatments administered to diverse samples of smokers would facilitate the development of accurate treatment algorithms. Although smokers are encouraged to quit each year, and millions make an attempt, most fail, leaving them vulnerable to early death and disability. This must be attributed, in part, to researchers’ failure to provide smokers and clinicians with accurate data on which treatments work best for which smokers.

Our limited progress in developing improved tobacco dependence treatments may be due to an additional factor; viz. we have little understanding of how current treatments work. For example, we don’t know whether treatments work by suppressing urges, enhancing positive affect, or reducing stress reactivity. Knowledge about mechanisms provides the only rational basis for understanding treatments and promoting treatment improvement and development.[15-17] Such knowledge provides a rational basis for treatment assignment as well. Understanding how treatments work can tell us not only what treatments *are* doing, but also what they are *not* doing: e.g., not reducing urges or not reducing post-quit irritability. With the knowledge of which mechanisms are not addressed by current treatments, new treatments can be developed to address these lacunae. Finally, knowledge about mechanisms may provide valuable information about the nature of addiction. For instance, if the suppression of urges is a sine qua non of successful treatment, this suggests that urge severity/intransigence is a potent motivational force in maintaining addictive behavior.

This proposal is designed to address core issues regarding how smoking cessation pharmacotherapies work, how well they work, and for whom they work. We will gather the best evidence to date on:

- The relative efficacies of major types of pharmacotherapies
- Mechanisms that are responsible for the pharmacotherapeutic effects
- Which smokers should get which treatment (evidence-based treatment algorithms)

Such information is vital for the optimal use of existing pharmacotherapies, and for the development of new pharmacotherapies.

B. Background And Significance

Numerous clinical trials have examined the efficacies of cessation pharmacotherapies, but only a handful have directly compared the efficacies of different pharmacotherapies within the same study. In general, these studies show that the nicotine gum, patch, spray and inhaler all have comparable efficacy. For instance, a recent study[18] compared the efficacies of different nicotine replacement therapies (NRTs: gum, patch, inhaler, and nasal spray), finding little difference among them (e.g., these agents produced similar continuous abstinence rates at 12 weeks: i.e., 20 - 24%). Meta-analytic studies also suggest similar efficacies for these NRTs.[2] However, one recent study reported a somewhat higher 6-week abstinence rate for the patch (21%) than for the nasal spray (13.6%).[12] Another study[11] comparing the efficacy of the nicotine patch with bupropion showed that bupropion was superior to the nicotine patch at both 6- and 12-month follow-up. A similar finding was recently reported in an additional (nonrandomized) study where individuals were allowed to select between nicotine patch and bupropion treatments.[19] While there are not enough data to conclude that bupropion is superior to the nicotine patch, the data currently suggest that this is so, particularly for women.[13,20]

Given the existence of these earlier studies, why conduct additional head-to-head comparisons of different treatments? First, since these studies were published, a new promising pharmacotherapy (i.e., nicotine lozenge) has been shown to be efficacious relative to placebo. No data regarding the comparative efficacy of this new agent vs. more established pharmacotherapies have yet been published nor has any study examined any combination treatments involving the nicotine lozenge. It is important to compare new agents with existing agents. Second, it is cost-effective to promote the most efficacious/effective therapies available. If one drug is found to be superior to others, it should be promoted above them. Third, a study comparing multiple drugs in multiple groups will permit development of efficient treatment algorithms to optimize use of available agents.[21] Finally, differences in the effects of specific agents may reflect different mechanisms of action. Uncovering the potentially diverse ways in which pharmacotherapies support abstinence may help us identify new treatments or treatment combinations that target multiple pathways to cessation. For these reasons this proposal calls for placebo-controlled, head-to-head comparisons of the following treatments for tobacco dependence: nicotine lozenge, nicotine patch, bupropion, a combination of the nicotine patch and nicotine lozenge, and a combination of bupropion and the nicotine lozenge.

Agents to be Compared

Nicotine patch. The nicotine patch will be included in this study because it is the most commonly used pharmacotherapy for smoking cessation.[18,22,23] Given that so many smokers use the nicotine patch, it is of great public health importance to determine its efficacy relative to other agents. In addition, we include the patch in order to evaluate the efficacy of the nicotine patch + nicotine lozenge combination. Without including the nicotine patch alone as a treatment condition it would be impossible to determine whether the combination treatment is efficacious because of the patch per se vs. the combination of NRTs. Finally, it is important to examine the efficacy of the patch since current data suggest that the patch may be less efficacious than in the past. Recent studies have cast doubt on its efficacy[11] and additional evidence suggests that patch efficacy may have declined over the past 5-10 years.[6,24]

Nicotine lozenge. It is vital to determine the relative efficacy of the nicotine lozenge for several reasons. First, it is relatively new and its efficacy should be examined in additional populations of smokers. Second, early data on the lozenge suggest that it is both highly acceptable to smokers and strikingly efficacious.[25] Third, the lozenge is available over-the-counter (OTC), which should promote broader use than prescription medications and therefore have a greater public health impact.

Table 1. Continuous abstinence rates for nicotine lozenge

	Low Dependence (2-mg)			High Dependence (4-mg)		
Time (Weeks)	Active (N=459)	Placebo (N=458)	OR	Active (N=450)	Placebo (N=451)	OR
6	46.0	29.7	2.1	48.7	20.8	3.7
12	34.4	21.6	2.0	35.3	14.0	3.4
24	24.2	14.4	2.0	23.6	10.2	2.8
52	17.9	9.6	2.1	14.9	6.2	2.7

*Reprinted from Shiffman et al., (2002).

The nicotine lozenge may be highly acceptable to smokers because it does not share some limitations of other forms of NRT. For instance, it does not produce nasal irritation (spray), require a special chewing technique (gum), adhere to dental work (gum), or create embarrassment when used in social situations (inhaler, spray[25]. In the Shiffman[25] study the lozenge was well tolerated and used at high

rates. In addition, the older NRTs, when used in a quit attempt, produce blood nicotine/cotinine levels that are only a fraction of those produced by precessation smoking[26,27] whereas 2- and 4-mg lozenges deliver some 25 to 27% more nicotine during a quit attempt than do the 2- and 4-mg nicotine gum.[28] Moreover, the lozenge permits acute administration whereas the nicotine patch does not. Therefore, the lozenge provides a way to cope with urges in a relatively rapid, effective, non-aversive manner. The efficacy data presented by Shiffman et al.[25] are quite encouraging (Table 1) in both Low- and High-Dependence smokers. These tabled data show that both doses of the lozenge were efficacious, with the high-dose lozenge producing odds ratios that rival or exceed those of the prescription drug bupropion (bupropion typically produces OR's in the 2.1 – 2.3 range).[2,29] In sum, the nicotine lozenge was targeted for evaluation in this proposal because it has been little studied, initial data on its acceptability and efficacy are quite encouraging, and it is available OTC. These characterizations rest upon a fairly modest database, which underscores the importance of doing research described in this proposal.

Bupropion. There are many reasons for selecting bupropion as a comparison cessation pharmacotherapy. First, aside from nicotine replacement products, bupropion is the only first-line cessation pharmacotherapy recommended in the PHS Guideline[2],based upon its efficacy and safety. One other antidepressant, nortriptyline, may have similar efficacy,[29,30], but it is associated with a higher rate of side effects and has a narrower range of therapeutic dose[30] and is not used nearly as widely as bupropion for smoking cessation. Second, the available evidence suggests that bupropion may be more efficacious than NRTs. Specifically, in head-to-head comparisons it was shown to be superior to the nicotine patch.[11,19] If bupropion is indeed more efficacious than OTC NRTs such as the patch or the lozenge, it is vital to know this so that smokers can be encouraged to seek out a prescription for this agent, and so that insurers and health care systems can be encouraged to make this treatment available. However, if it can be shown that an OTC medication (e.g., the nicotine lozenge) is equal or superior to bupropion, documenting the superiority of these OTC agents might result in more successful quit attempts and greater reductions in smoking prevalence.[31,32] Finally, there is reason to believe that bupropion and NRTs may have differential efficacy in subpopulations of smokers (i.e., women, affectively vulnerable).[13,33] Therefore, comparing NRTs with bupropion in the same study might allow us to develop effective treatment-matching algorithms so that each smoker receives optimal treatment.

Nicotine patch + nicotine lozenge. Research has generally supported the efficacy of NRT combinations. For instance, the PHS Guideline[2] stated that, "Combining the nicotine patch with a self-administered form of nicotine replacement therapy (either the nicotine gum or nicotine nasal spray) is more efficacious than a single form of nicotine replacement…" (p. 77; also, cf Silagy et al.[4]). At the time that conclusion was drawn, only three studies had evaluated NRT combinations. However, since the time of the Guideline meta-analyses, Bohadana[34] compared the 15-mg nicotine patch + inhaler with the inhaler alone. Results were similar to earlier studies using multiple vs. single forms of NRT. Although no significant differences were obtained at 12-month follow-up, a survival analysis indicated significant treatment effects at one year, and results favored the combined therapy condition across all time points. In addition, Croghan[12] recently compared a nicotine nasal spray + patch combination vs. either drug alone. In general, results favored the combination. At 6 weeks, the CO-confirmed point-prevalence abstinence rates were 20.7% (patch alone), 13.6% (spray alone), and 27.1% (combination). Differences were not statistically significant at 6-months post-treatment, and the authors speculate that the short period of pharmacotherapy treatment (6 weeks) may have contributed to high relapse rates across conditions. We recently found a similar drop-off at the end-of-treatment with a gum + bupropion combination[35], which further suggests that the lozenge's 12-week use period may be critical to the longterm success that we expect with this agent. Finally, in the Croghan[12] study, the high rates of adverse events produced by the spray may have reduced the efficacy of the combination therapy relative to the patch per se.

Thus, most evidence suggests that combination therapy with NRT agents is superior to monotherapy with NRT agents. The superiority of a patch + gum combination may be due to the fact that the patch supplies 24-hour exposure to nicotine while the gum permits individuals to respond to crises with self-administration of more nicotine. Similarly, we suspect that the lozenge will act through a different mechanism (acute urge reduction) than the patch (tonic suppression of withdrawal). Thus, combining the two agents should have an additive effect. We contend that the lozenge will produce even more impressive results when paired with the nicotine patch than does the nicotine gum. This is because the lozenge has, thus far, shown impressive efficacy when used in isolation and may be more readily self-administered than the gum.

Bupropion + nicotine lozenge. In the fifth active pharmacotherapy condition participants will receive bupropion plus the nicotine lozenge. We have chosen this untested treatment combination despite the fact that some data suggest that NRTs do not enhance the efficacy of antidepressants.[11,36] Our reasons for evaluating this combination are both theoretic and empiric. First, since bupropion and NRTs have distinct neuropharmacologic mechanisms of action (although both have dopaminergic effects), it is logical that combining two such dissimilar agents might produce truly complementary effects. For instance, the effects might be either interactive, or one drug would work with some smokers while the other would work for different smokers, thereby increasing the net effect. Moreover, the two agents involved differ not only with respect to likely neuropharmacologic mediation, but also with respect to use strategy (e.g., prn use, ability to respond to crises, half-life). In addition, recent UW TTURC data suggest that prn NRT plus bupropion may, in fact, be more efficacious than bupropion alone, especially for females.[35] Preliminary Study 1 also showed that the bupropion plus gum combination was superior to bupropion alone in suppressing withdrawal symptoms. However, once medication use was discontinued (at 8 weeks post-quit), abstinence rates fell sharply. We posit that the bupropion plus nicotine lozenge combination will be relatively efficacious in the proposed research because we believe participants will use the lozenge at higher rates than they used the gum. In sum, the never before studied combination of these two highly effective medications holds promise based on the efficacy of previous combination therapies and Preliminary Study 3 data.

In closing, it is remarkable that so little is known about the relative efficacies of treatments for a condition that is so common, so commonly treated, and so costly if not treated successfully. Identifying the most efficacious agents available may lead to important changes in treatment planning and policy that will encourage efficient use of treatment resources.

Moderation of Treatment Effects

In order to treat smokers optimally, we need to know which therapies work best with which smokers. Cessation pharmacotherapies are used by millions of people but they may not work equally well for all people, making it essential to know how to match therapies to individuals. While some treatment algorithms exist, at present there are no clear, empirically grounded algorithms to guide the matching of patients with the multiple pharmacotherapies currently available.[37] Hughes has noted that, "Because we have no head-to-head comparisons of the therapies, because no treatment has replicated evidence of superior efficacy or adverse event profiles, and because no method to match smokers to a particular treatment has been empirically validated, patient preference should be a primary basis for choice among treatments"[38] (p. 75). Unfortunately, patient preference may not provide optimal algorithms for matching patients with the most efficacious interventions.[39]

The development of empirically based algorithms requires the detection of interactions between patient characteristics and treatment efficacy. An ideal algorithm would involve an individual difference that is easy to assess and that predicts a response to treatment such that one value predicts little response to a treatment and a different value is associated with great benefit. Ideally, smokers not benefiting from one type of intervention would benefit from a different type. Authorities on behavior change have noted that it is extremely difficult to identify reliable interactions between types of people and types of treatments.[21] However, recent efficacy data suggest that smoking treatment may be one area in which such interactions are reliable and of a magnitude that has public health significance. These data *suggest* reliable interactions between individual differences (e.g., dependence, gender, and affective/depression status) and cessation pharmacotherapies.

Gender. Numerous clinical trials have shown that women tend to have lower abstinence rates than do men following either NRT or placebo treatment.[14] In fact, in some studies there is no long-term benefit of NRT treatment among women.[13] Bupropion, however, tends to boost long-term abstinence rates among women, with the benefit being similar to that found among men.[13,20,40] The magnitude of this effect may be considerable. In the Smith[13] study, one-year abstinence rates for women were 11% for those using the patch, and 26% for those using bupropion. Such findings underscore the need for new head-to-head comparisons among pharmacotherapies since much of the older literature does not examine gender differences in cessation,[41] and it seems vital to determine if a prescription drug (e.g., bupropion) is necessary in order for women to attain optimal

results or if there is an OTC medication that is highly efficacious for women. The importance of this derives from the fact that women may have greater difficulty quitting than do men,[14] smoking prevalence among women (19.4% in 2002) is now relatively close to the prevalence among men (24.0% in 2002),[42] and the majority of quit attempts do not occur in the context of formal, professional assistance. Therefore, OTC aids may have great utility among women. In the study proposed here, it is conceivable that the novel combination of OTC NRTs (nicotine patch and lozenge) will perform as well as prescription bupropion in women. However, if it is indeed the case that women are less dependent upon the pharmacologic effects of nicotine than are men,[43,44] then it may be that even a highly efficacious combination NRT will not be as effective as bupropion.

Nicotine dependence. Research has shown that nicotine dependence as assessed by questionnaires[45,46] interacts with nicotine gum dose in influencing cessation success. In these findings, highly dependent smokers benefit from 4-mg nicotine gum, but not from 2-mg gum.[47] Two-mg gum, on the other hand, benefits less dependent smokers, and such smokers do not appear to require the higher gum dose.[47] A recent study[48] replicated these results among smokers when random assignment to 2- or 4-mg gum was blocked on dependence level. The relation between dependence and success with combination pharmacotherapy that includes dual NRT has not been tested to date. However, it seems logical to believe highly dependent individuals would benefit especially from the addition of a second NRT. In theory, highly dependent smokers are most likely to experience severe withdrawal symptoms, prompting relapse back to smoking. The symptomatic relief provided by dual NRT, one creating a steady state of nicotine and one being used to cope with urges, should prevent relapse, especially among highly dependent smokers. Therefore, we predict that high scores on one or more dependence measures should predict magnitude of benefit from the addition of a second NRT. On the other hand, it is possible that highly dependent smokers would profit most from a combination of pharmacotherapies that produces its actions via multiple distinct routes. If this is the case, it may be that the bupropion plus lozenge combination will be most efficacious with highly dependent smokers.

A key to investigating this issue is to use psychometrically sound dependence measures that are valid and simple to use. We will use the FTND[49] and a new measure of nicotine dependence (the WI-PRISM[50], Preliminary Study 2) to investigate the relation between dependence and treatment success. There is some evidence that the WI-PRISM (and individual WISDM-68 [Preliminary Study 3] scales which will also be available) may have greater validity for this purpose than the FTND,[51] thereby increasing the importance of new research on this topic.

Negative affect/depression. In addition to nicotine dependence, it is possible that individuals with a history of depression will benefit more from one form of pharmacotherapy than another. Some evidence indicates that such individuals face an especially high risk of relapse following a quit attempt[52-54] (but see Hitsman et al.[55]), and may respond better to certain treatments. For example, Hall[56] and Zelman[57] found that some types of counseling were especially beneficial to smokers with a history of depression (but cf. Kahler et al.[58]). Because of their ease of standardization, we believe that pharmacotherapies will produce more consistent evidence of differential efficacy than counseling. Smith et al. reported that smokers with a history of depression benefited more from bupropion treatment than from nicotine replacement treatment (i.e., the nicotine patch).[13] This is consistent with recent evidence that bupropion may “work” via suppression of negative affect.[33] Thus, bupropion may reduce the negative affect that smokers with a history of depression would otherwise experience during a quit attempt, and this effect reduces relapse risk.

It is important to note that a single question on history of depression may not provide the optimal predictor of differential treatment effects. Therefore, several measures of affective vulnerability will be used to predict differential response to treatment, and ultimately, for a treatment assignment algorithm. Therefore, we will use several brief measures of negative affect vulnerability in tests of moderation. Specifically, we will use the Depression Proneness Inventory (DPI[59]), select items from the Negative Emotionality Scale of the brief MPQ,[60] and the “month version” of the NPANAS.[61] Thus, in addition to assessing depression diagnosis (current & historical) we will assess trait negative affectivity (the NES & NPANAS) and depression diathesis (the DPI). The DPI has predicted differential response to smoking treatment in previous research.[57] The NCS-R-CIDI will be available to determine correspondence between continuous measures and actual diagnoses.

Challenges. An optimal algorithm is one that is easy to apply in a clinical setting and that results in a high overall success rate across the entire sample of smokers. In the proposed study, we will strive to create a sophisticated, easy to use treatment-matching algorithm that examines the impact of multiple individual difference variables, such as gender, dependence, and negative affect. Because we suspect all of these variables will confer important information about treatment response, we will use a multivariate approach. However, individual difference variables may be highly correlated (e.g., gender & depression history), making it difficult to determine which variable should be used for treatment assignment (e.g., Smith et al.[13]). We will use decision tree analyses since they are effective at contrasting inter-related predictors. For instance, if depression history is somewhat redundant with gender, and conveys similar information regarding optimal treatment, it may be warranted, for the sake of simplicity, to ignore status on depression history and use only gender and dependence level for assignment. Conversely, data may suggest higher-level algorithmic rules such that interactions exist among the predictors (e.g., men should routinely be given combination NRT unless they have a prior history of depression, in which case they should be given bupropion). The effectiveness of algorithms will vary across populations such that an individual difference variable may be more or less useful depending upon base-rates of characteristics. Sensitivity analyses will be conducted to determine how well an algorithm fares across different modeled base-rates. Finally, other moderators (e.g., alcohol abuse/dependence history [not current abuse/dependence[62]]) will also be examined to determine whether these factors affect the utility of generated rules. In sum, it is important not only to determine which treatments produce optimal outcomes overall, but also, which produce optimal outcomes in different groups of smokers. There is already strong evidence that treatment X individual difference interactions exist, however, it is vital to discover the specific nature of these interactions, and to discover how they are manifested in the context of current, efficacious interventions.

Mechanisms

At present we have only a rudimentary understanding of how efficacious smoking cessation pharmacotherapies work. Some data suggest that pharmacotherapies work by reducing withdrawal symptoms. This is consistent with the observation that all of the efficacious pharmacotherapies do, in fact, reduce withdrawal symptoms.[11,63-65] In addition, there is mounting evidence that withdrawal symptoms prompt or trigger lapses/relapse.[63,64,66] Thus, logically, if withdrawal prompts relapse, then pharmacotherapeutic management of withdrawal symptoms should reduce relapse. However, other research and theory suggest that withdrawal does not instigate relapse[67] and that efficacious treatments do not work via withdrawal reduction.[68,69] Thus far, research on mechanisms is only suggestive and not definitive. This is because: (1) *Formal mediational analyses are very rarely conducted*. Of the many clinical trials of cessation pharmacotherapies conducted, only a handful attempted to determine which effects of treatment were determinant of success. (2) *Mechanisms are typically not measured in a multidimensional manner*. For instance, withdrawal symptoms, possible mediators of treatment effects, are typically studied in one dimension (e.g., severity[67]). Our research shows that there are multiple dimensions of withdrawal (e.g., trajectory, volatility[63,64]) that are affected by treatment and independently predictive of relapse (see Preliminary Studies 4 and 5) and thus merit mediational analyses. (3) *Mediators are not measured in real time.* Previous attempts to uncover mediators[33] used paper and pencil assessments of mediators that involved recollection and integration of events across lengthy time periods (but see Preliminary Study 5). This may produce error due to recall difficulties, poor integration of data across time, and filling-in of data after long delays. (4) *Few mechanisms have been studied.* As noted above, some research has examined whether withdrawal or negative affect suppression accounts for pharmacotherapy efficacy. However, almost no other mechanisms have been examined.

Because of the above limitations, the few tests of mediation that have been conducted have shed relatively little light on treatment mechanisms. For instance, Lerman[33] found evidence that reductions in negative affect may partially mediate bupropion's efficacy. However, the magnitude of that effect was quite small, suggesting that other factors play a bigger role. In addition, the mediator (negative affect measured at 4 discrete time points: pre-treatment, pre-quit, post-quit, and end of treatment 8 weeks after the quit date) and the outcome variable (abstinence at end-of-treatment) were contemporaneous with one another so that reciprocal effects challenge interpretation.[70] It is remarkable that so little is known about the mediation of pharmacotherapy effects because understanding causal process is a key goal of the scientific enterprise.[71]

It is vital to identify treatment mechanisms. First, if we can discover how treatments work, this may provide insights into how to improve treatments and make them more efficacious. For instance, if suppression of negative affect accounts for the efficacy of a pharmacotherapy, then use of the pharmacotherapy could be modified so as to achieve maximal impact on these symptoms. Or, if we find that treatments do *not* affect a potential mechanism (e.g., no treatment actually reduces affective response to stressors) then researchers could target this potential mechanism in the development of new treatments (e.g., perhaps complementing pharmacotherapy with counseling that targets stress coping). Second, information on mechanisms might suggest matching strategies. For instance, highly dependent individuals might be given treatments that work via withdrawal suppression. Third, characterizing mechanisms might provide variables that can be tracked for treatment modification or dosing. That is, one could adjust dosing based upon the measured mechanism in order to achieve optimal clinical results. An example of this would be tracking morning urge reports over time[72] in order to adjust pharmacotherapy dose. Medication types or levels might be adjusted until urge reports consistently fall below an empirically validated threshold. Finally, it seems very important to determine if our theories of how our treatments work are correct. If our theories are not supported by mediational research, we need to develop new theoretical models that can foster new, more effective interventions.

Candidate mechanisms. A review of relevant research and theory helped us identify the following potential mechanisms of pharmacotherapy effects:

- *Reduction of withdrawal symptoms/negative affect*. Withdrawal (including negative affect) will be measured using state-of-the-art electronic diary (ED) assessment methods that allow us to assess symptoms in real time and in the context of participants' daily lives. In addition, we will strive to determine which particular aspects of the withdrawal *profile* are involved in mediation. For instance, do treatments work because they prevent growth in symptoms or reduce fluctuations in symptoms? In addition, it is vital to determine when, relative to the start of treatment and the quit date, mediation occurs. Is suppression of symptoms in the first 48 hours important, or is maintenance of effects over longer timeframes critical?
- *Maintenance of positive affect*. In addition to increasing negative affect, withdrawal has been found to decrease positive affect[73] and the rewarding properties of non-drug reinforcers.[74-76] It is possible that bupropion, which has activating dopaminergic actions, may counteract the depression-like anhedonia that accompanies withdrawal.[66] Therefore, participants treated with bupropion may not only report elevated levels of positive affect, but also greater enjoyment from appetitive activities.
- *Reduction of stress reactivity.* Considerable research suggests that stressors may provoke lapses/relapse.[77] It is possible that treatments work by reducing affective/urge reactions to stressors. Thus, electronic diaries will be used to measure the occurrence of different types of stressors (e.g., social/interpersonal, work, etc.) and affective and other reactions to such stressors.
- *Reduction of craving in response to temptation events.* Recent data from our site (Preliminary Study 4) indicate that urge reactivity to temptation events doubles from pre-quit to post-quit. This, of course, may be highly determinant of lapses/relapse, and reduction of such reactivity may account for treatment effects. Moreover, Preliminary Study 5 data also suggest that craving reduction may, in part, mediate the impact of bupropion on abstinence. Using ED data we will assess temptation events and participants' urge/craving and affective reactivity to such events. We predict that a steady-state pharmacotherapy (e.g., patch), combined with an effective prn NRT (i.e., lozenge) that allows smokers to effectively self-medicate their cravings, will be the most effective pharmacotherapeutic treatment for reducing cue-elicited urges.
- *Detoxification of slips.* Some research has suggested that agents such as bupropion might work by reducing the likelihood that a slip will lead to relapse.[64, 65, 78] We will use ED data to examine this issue by exploring whether a slip is more or less predictive of full relapse (and the latency to full relapse) across the different treatment conditions.

In sum, while a handful of investigators[33,79] have begun to try to identify how and why treatments work, this work is in its infancy. Investigators have addressed few treatments, few mediators, and we currently possess little well validated information on this topic.

Genetics

Genotype will be determined in order to gauge the relations of key candidate genes with important indicators of nicotine dependence (e.g., withdrawal dimensions, relapse latency, and multifactorial measures of nicotine

dependence: the WISDM-68). In addition, we will analyze gene-treatment interactions with respect to outcome to explore substrata of treatment effects. There is extensive biological evidence for the involvement of nicotinic receptors in tobacco dependence.[80-83] A recent human study of haplotypes of four novel single nucleotide polymorphisms in the beta-2 subunit (CHRNB2) did not confirm the hypothesized differences in either smoking initiation or progression to dependence.[84] However, as the authors point out, molecular studies of only one subunit may give an incomplete picture of the function of nicotine receptors. We propose, therefore, to test all common haplotypes for the 8 common alpha and beta subunits of the nicotinic receptors (see Table 2).

Table 2. Nicotine-receptor Candidate Genes

	Symbol	Gene size	cDNA size	#SNPs	# Confirmed	Name
Nicotinic receptors						
	CHRNA1	18.1	1.7	7	0	alpha1
	CHRNA2	20	2.7	20	0	alpha 2
	CHRNA3	202	1.5	97	0	alpha 3
	CHRNA4	17.8	3.3	9	0	alpha 4
	CHRNA5					alpha 5
	CHRNA6	17	1.7	4	1	alpha 6
	CHRNA7	82.8	1.5	86	0	alpha 7
	CHRNA9	no genomic seq.				alpha 9
	CHRNA10	7.3	1.9	0	0	alpha 10
Chr.17	**CHRNB1**	9.5	1.6	1	0	beta 1
	CHRNB2	10.4	2.4	6	0	beta 2
	CHRNB3	7.6	1.2	3	0	beta 3
	CHRNB4	15	2.4	2	0	beta 4
	CHRND	18.8	1.7	5	0	delta
	CHRNG	7.5	1.5	6	0	gamma
Chr.17	**CHRNE**	6.8	2.4	6	0	epsilon

In addition to nicotinic receptors, other candidate genes have been identifiedand will be evaluated. These include ones involved in nicotine metabolism (CYP2A6, CYP2D6), dopamine re-uptake (SLC6A3, SLC18A1, SLC18A2), dopamine receptors (DRD1-5), dopamine metabolism (MAOA, MAOB, COMT, DBH, DDC), ACh re-uptake and metabolism (SLC18A3, ACHE, CHAT), as well as positional candidates as per genome wide linkage studies (CACNB4, TNFAIP6). These candidates include two genes identified as positional candidates from genome wide linkage studies.

In terms of the dopaminergic system, a positive association has been reported between the 9-repeat allele of the DAT1 VNTR locus and smoking,[85] between smoking behavior and DRD1,[86] between the dopamine transporter gene and smoking cessation,[87] and between the A1 allele of the DRD2 gene and various smoking behaviors[88,89] (although see Spitz et al.[90]). In the Bergen[91] genome-wide linkage study, the authors note that modestly positive results for DBH (chromosome 9) were detected using ever/never smoked (p=0.01) and a log transformed pack-year (p=0.005) analysis. The chromosome 5 region most positive in the Duggirala[92] study contains the DRD1 locus. A recent study[93] showed a gene X gene interaction on prolonged abstinence involving SLC6A3 and DRD2. The DRD2-A2 genotype in the presence of the SLC6A3-9 genotype (compared with the SLC6A3-10 genotype) had higher abstinence rates and a longer latency to relapse (also see Caporaso[94]). Also, an association has been found between smoking initiation and two polymorphisms in the tryptophan hydroxylase (TH) gene.[95]

Genes involved in nicotine activity and metabolism may also be involved in nicotine addiction and smoking behavior. Microsomal cytochrome P450 2A6 (CYP2A6) has been suggested as a protective genetic factor against nicotine dependence.[96] Two mutations, each producing an inactive enzyme, were both implicated in this study as protective factors. The study reported that a single copy of either mutation was sufficient to produce impaired nicotine metabolism, found more often in controls than nicotine dependent smokers (p=0.04), see [97,98] for concerns regarding genotyping in this study. A subsequent study in a population of lung cancer patients found only a statistical trend in nonsmokers having two copies of the mutation.[99] A positive association has also been reported with pack-years of smoking and another CYP450 enzyme, CYP1A1.[100]

C. Preliminary Studies

Since 1992, the Center for Tobacco Research and Intervention scientists have conducted 24 randomized smoking cessation clinical trials and developed valid assessments of tobacco-related constructs. More than 1500 community smokers enrolled in the first UW TTURC studies, including more than 1100 smokers who agreed to participate in demanding ecological momentary assessment. The proposed research will build upon recent UW TTURC accomplishments in clinical trial methods, statistical modeling, assessment, and genetic analyses as described below.

Preliminary Study 1. Bupropion Monotherapy and Combination Therapy with Nicotine Gum.[35]
This randomized placebo-controlled double-blind clinical trial compared the efficacy of bupropion alone and in combination with nicotine gum in a large (N=608), ethnically diverse (22% African American) sample of smokers.

Results indicated that use of gum and bupropion increased end-of-treatment cessation rates, particularly for women and suppressed withdrawal symptoms, relative to a placebo condition.

Preliminary Study 2. The Wisconsin Prediction of Relapse in Smoking Measure (WI-PRISM).[50]
A five-item instrument to assess relapse proneness in clinical settings was developed using data collected in Preliminary Studies 1 and 5 from the first UW TTURC. The two item-parcels in the WI-PRISM (physical dependence and environmental risk) predicted relapse, and total WI-PRISM scores predicted relapse better than the Fagerström Test of Nicotine Dependence[49]. Environmental risk uniquely predicted relapses occurring between the end of treatment and the 6-month follow-up. WI-PRISM scores were related to treatment response, such that smokers with lower scores benefited from the addition of a second pharmacotherapy in a way that high scorers did not. The WI-PRISM is a promising measure for a pharmacotherapy algorithm (see Projects 1 & 3).

Preliminary Study 3. The Wisconsin Inventory of Smoking Dependence Motives (WISDM-68).[51]
The WISDM-68 is a theoretically derived, multifactorial measure of tobacco dependence that was developed and validated using data from 775 daily and non-daily smokers. All 13 scales had good internal consistency (alphas >.85). WISDM-68 scores predicted smoking heaviness and select subscale scores predicted relapse at the end of treatment and withdrawal experiences in Preliminary Study 1. This inventory will be administered to participants in the proposed Project 1.

Preliminary Study 4. Life Before and After Quitting Smoking.[101]
In this prospective cessation study, 90 adult smokers completed four daily nicotine withdrawal assessments using an ED for up to ten weeks. Multilevel modeling techniques were used to estimate individual growth-curves for withdrawal symptoms to describe what life was like before and after a quit attempt. Results replicated previous TTURC findings[63] showing that smokers differ in withdrawal experiences and symptom reactivity to smoking. Larger increases in distress prior to quitting predicted early relapse. After quitting, participants became more symptomatically reactive to smoking cues and quitting appeared to release large individual differences in affective symptoms. As in this study, participants in the proposed project will record data using an ED both before and after a quit attempt and multilevel models will be fit to the data.

Preliminary Study 5. Negative Affect in Smoking Relapse: Withdrawal and Treatment Mediation.[102]
In this large (N=463) clinical trial examining the efficacy of bupropion and individual counseling, we conducted intensive EMA assessment of potential mediators of treatment effects. Participants carried EDs for six weeks, responding to five to seven prompts per day. Estimates of symptom growth derived from multilevel models were submtted to mediational analyses. Results showed that combined pharmacotherapy and psychosocial intervention led to reduced craving severity and differences in craving trajectories, and that these changes in craving experience predicted subsequent abstinence. Similar analytic strategies will be used in Project 1.

Preliminary Study 6. Exploring the Genetic Correlates of Tobacco Dependence and Cessation.
Wisconsin has shipped to the University of Utah Hunstman Cancer Institute over 1100 DNA samples of smokers and community controls. All of these samples have undergone quality control at Utah and have been diluted to a single concentration, 200 mg/ml, to facilitate subsequent DNA sequencing and SNP genotyping. After identification of common haplotypes in the nicotinic subunits in a subset of these smokers and controls, we will identify tagged SNPs and genotype them in the remaining samples. A total of 192 samples from smokers and nonsmokers (two trays of 96) have been defined. Amplification and sequencing has begun on the first tray of 96 samples for the CHRNA3 and CHRNB4 genes. A subset of smokers (n=80) with extreme values on a novel nicotine dependence index have been identified and will be compared genotypically with one another and a sample (n=50) of nonsmokers as well. Genetic data from participants in the proposed research will be submitted to similar and additional analyses (e.g., examining the genetic correlates of responses to different pharmacotherapies).

D. Research Design And Methods

Participants will be randomized to six different pharmacotherapy conditions (see Figure 1). All six types of pharmacotherapy intervention, including the placebo condition, will be accompanied by brief counseling.

Figure 1. Randomization procedure for the study.

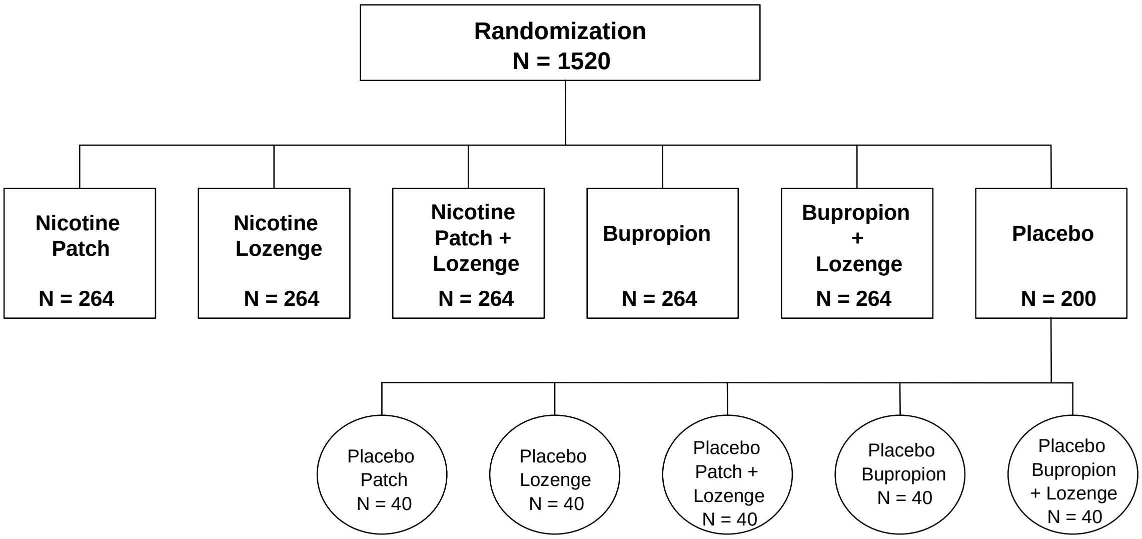

It should be noted that this randomization figure does not reflect blocking that will occur by sex (male/female), ethnicity (minority/non-minority) or site (Madison/Milwaukee).

Timing of Events.
The study involves four basic phases: Recruitment & Orientation, Pre-quit Assessment, Treatment & Intratreatment Assessment, and Follow-up. Table 3 portrays the timing of study events. All participants will receive reminder calls prior to their clinic visits.

Table 3. Schedule of Assessment and Treatment

**Evaluation**	**Orient.**	**Visit 1**	**Visit 2**	**Visit 3**	**Visit 4**	**Visit 5**	**Visit 6**	**Visit 7**	**Visit 8**	**Visit 9**	**Visit 10**
		**Wk. -3**	**Wk. -2**	**Wk. -1**	**Quit Day**	**Wk. 1**	**Wk. 2**	**Wk. 4**	**Wk. 8**	**Wk. 26**	**Wk. 52**
Sign Informed Consent and HIPAA	X										
Demographics	X										
Smoking History	X										
Wisconsin Inventory of Smoking Dependence Motives (WISDM-68)	X										
Wisconsin Predicting Relapse in Smoking Measure (WI-PRISM)	X										
Fagerström Test of Nicotine Dependence (FTND)	X										
Wisconsin Smoking Withdrawal Scale (WSWS)	X			X	X	X	X	X	X	X	X
Multidimensional Personality Questionnaire-Short Form (MPQ-S)	X										
Positive and Negative Affect Scale (PANAS)	X			X	X	X	X	X	X	X	X
Depression Proneness Inventory (DPI)	X										
Social Readjustment Rating Scale (SRRS)	X										
International Physical Activity Questionnaire (IPAQ)	X										
PrimeScreen (dietary questionnaire)	X										
Food Frequency Questionnaire (FFQ)	X										
Vital Signs[a]		X	X	X	X	X	X	X	X	X	X
Concomitant Medications and Adverse Events		X	X	X	X	X	X	X	X	X	X

Suicidality Assessment		X	X	X	X	X	X	X	X	X	X
Complete Physical Exam		X									
Waist Circumference		X									
National Comorbidity Survey – Revised (NCS-R-CIDI)[b]		X									
Short Inventory of Problems (SIP-2R) – Alcohol related problems		X									
Social Network Interview		X									
Kansas Marital Satisfaction Scale (KMSS)[c]		X									
Quality of Life Inventory (QOLI)		X									
Pregnancy Test for women		X									
Ultrasound Carotid Artery Intima – Medial Thickness (Carotid-IMT)			X								
Ultrasound Brachial Artery Reactivity Testing (UBART) of Endothelial Function			X								
Exercise Stress Test			X								
ED Training			X								
Pedometer Training and Assessment			X								
Blood Draw[d]			X								X
Smoking Status[e]				X	X	X	X	X	X	X	X
Counseling				X	X	X	X				
Medication Distribution				X	X	X	X	X			
Return Unused Medication					X	X	X	X	X		
ED Data Upload[f]				X	X	X	X				
**Time Required For Each Visit (in hours)**	**2**	**3**	**3**	**.5**	**.5**	**.5**	**.5**	**.5**	**.5**	**.5**	**.5**

[a] Vital signs includes carbon monoxide, blood pressure, weight, temperature, pulse, and height at Visit 2. [b] NCS-R-CIDI, which should last on average 90 minutes, includes a Screener and the following modules: Depression, Irritable Depression, Panic Disorder, Specific Phobia, Social Phobia, Agoraphobia, Generalized Anxiety Disorder, Suicidality, Use of Services, Alcohol Use, Tobacco Use, Chronic Conditions, Neurasthenia, 30-Day Functioning, 30-Day Symptoms, Obsessive-Compulsive Disorder, Worries and Unhappiness. [c] This will be administered to participants who are married or who have a domestic partner. [d] Blood will be drawn for the following tests: NMR lipid profile, creatinine, simultaneous fasting blood glucose and insulin, blood carotenoids, hemoglobin A1C, and high-sensitivity c-reactive protein. [e] Smoking status includes a review of the abstinence calendar, nature of relapse contexts, and new quit attempts. [f] See Table 4 for a list of EMA assessments

Recruitment & Orientation

Eligibility. 1520 individuals will be randomized. Inclusion criteria will be that individuals: report smoking at least 10 cigarettes/day for the previous 6 months, produce a breath sample with a CO level > 9 ppm, report being motivated to quit, able to read and write English, agree to respond to EMA prompts throughout the day, and plan to remain in the treatment catchment area for at least 12 months after the initiation of treatment. Medical screening will ensure that participants can safely be prescribed any of the medications to be used (e.g., no uncontrolled hypertension, history of bipolar illness, recent myocardial infarction, recent history of alcohol dependence, seizure history, or use of MAO inhibitors or other contraindicated medications). In addition, any individual who reports thoughts of suicide or self-harm will be interviewed by a clinical psychologist, and those judged to be at risk for suicide will be excluded and offered referrals to appropriate treatment resources. All women of child-bearing potential will be given a pregnancy screening test and required to agree to use an approved method of birth control to prevent pregnancy during the course of the study. Finally, only one member per household will be allowed to participate in the study. We will not exclude individuals based upon their prior use of a study medication because we recognize that a high percentage of U.S. smokers have tried one or more of the pharmacotherapies we will use. We wish to sample from this general population of smokers rather than a smaller population of smokers who have never used a cessation aid previously. We will, however, statistically control for past use of study medications.

Recruitment. As in our previous research, we will advertise for participants via newspapers, radio, television, billboards, and other media. In addition, free media (e.g., press conferences) will be utilized. Past experience shows that, in Madison, WI, such recruitment strategies yield over 500 smoker enrollees per year. The proposed research will also occur in Milwaukee, WI, a city with a population of 1,500,741 in the major metropolitan area and significant racial/ethnic diversity. Our past experience conducting research in Milwaukee suggests the capacity to recruit an additional 800 participants per year. We will not run this study at this rate, but it is important

to note that our use of two sites allows us to conduct multiple trials with large sample sizes. Our recruitment procedures have allowed us to complete multiple large N studies over the past five years with studies often running simultaneously (Preliminary Study N's = 608, 463, 284, along with several highly selective pharmaceutical company clinical trials with combined N's > 300). Our success in recruitment is due to access to the largely unstudied population of smokers in Milwaukee, as well as to development of highly effective media recruitment campaigns.[103] In addition, over the last two years, the Wisconsin Tobacco Quit Line (WTQL) has received approximately 2880 calls from smokers in the Milwaukee area and approximately 1400 from smokers in the Madison area. The WTQL will be used in the proposed research to inform callers in the Madison and Milwaukee areas of the availability of this treatment research opportunity.

Advertisements and publicity will contain a phone number for individuals to call to contact study personnel. After calling this number, participants will undergo initial phone screening to rule out those with clear contraindications. The study will be briefly described, questions answered, and potentially qualifying individuals will be invited to attend an Orientation Session. Between 60 and 150 participants will be scheduled for each Orientation Session. Previous experience shows that between 50-75% of these actually attend the Orientation, and about 80-90% of these qualify to participate and enroll in the research study. These figures have held true across studies that differ dramatically in their participant burden.

Enrollment. At the Orientation Session the general requirements for participation will be reviewed (e.g., session attendance, need for follow-up, participation in assessments, participation in the proposed follow-up cohort study Research Project 2: *Natural History of Smoking & Quitting: Longterm Outcomes*, see below). In addition, participants will be informed of the nature of the treatments involved. They will be told that five different pharmacotherapy conditions will be studied and that participants will receive either one of those treatments or a placebo medication. In addition, participants will be told that everyone will receive counseling designed to aid them in their cessation attempt. After answering additional questions about research participation and treatment, participants will be asked to read and sign a consent form that contains appropriate HIPAA information.

Assessment

This study will involve assessment of a number of constructs using a variety of methodologies, including paper-pencil measures, ecological momentary assessment (discussed in detail below), interviews, and collection of physiological data and biological specimens. There are five main categories of constructs to be assessed in this study: 1) Tobacco-related constructs – tobacco use, dependence, and withdrawal; 2) Mental health – diagnoses of mental illness and social support; 3) Physical health – cardiovascular functioning, general physical health and disease states; 4) Lifestyle – alcohol, diet, exercise, quality of life, and stress; and 5) Psychosocial – personality, affect and social relations. One might wonder why we wish to collect certain data that do not seem directly relevant to cessation (e.g., disease states, diet, and exercise levels). These data will serve as baseline measures for a longitudinal study aimed at characterizing the long-term effects of cessation vs. continued smoking (Project 2: Longterm Outcomes). Because the longitudinal study will address how cessation, relapse, and continued smoking affect life, broadly conceived, we will collect a broad range of measures in the baseline period.

Tobacco-related Constructs

Tobacco use. Tobacco use history will measure age of first cigarette, number of years smoking, etc. On-going tobacco use will be assessed using ecological momentary assessment during the two weeks before and the two weeks after the quit attempt. After that period, tobacco use will be assessed using a daily diary through the end of treatment and then using time-line follow-back methods during telephone follow-up contacts.

Dependence and Withdrawal. Tobacco dependence will be assessed using the Wisconsin Inventory of Smoking Dependence Motives (WISDM-68),[51] the Wisconsin Predicting Relapse to Smoking Measure (WI-PRISM),[50] and the Fagerström Test of Nicotine Dependence (FTND).[49] These measures have been shown to predict dependence outcomes such as relapse and withdrawal severity.[50,51] While the reliability of the FTND is questionable,[51] it is very brief, and it has been used extensively in the past and this will permit a comparison of future results with previous findings (e.g., on the genetic basis of dependence).

Tobacco withdrawal will be assessed using the Wisconsin Smoking Withdrawal Scale (WSWS),[104] which has been shown to correlate highly with other measures of withdrawal, and is sensitive to both nicotine withdrawal and replacement. It is preferred to some other measures of smoking withdrawal since it contains scales with multiple items permitting determination of reliability. Participants will complete the WSWS at all study visits and they will also complete select WSWS items based on how they have felt in the last 30 minutes as part of the EMA battery. In addition to assessing withdrawal per se, participants will answer questions regarding the occurrence of temptation events since the last prompt and how they coped with them as part of the EMA paradigm described below.

Finally, participants will have the opportunity to volunteer to participate in a genetics study to assess the relations of genetic markers to tobacco dependence, withdrawal and cessation success. DNA sequencing will take place at the University of Utah Huntsman Cancer Institute (see Preliminary Study 6). It has four ABI 3700's and one ABI3730, which can run a total of 28,000 sequence ladders per week. SNP typing will be performed by the ABI SNPlex system. This system can detect 4500 SNPs in parallel in as little as 15 minutes. Extensive capabilities also exist in this center for bioinformatics that permit collection and analysis of DNA sequence, haplotype and SNP data.

Mental Health

Mental health will be assessed in this study using the new computer-assisted National Comorbidity Survey-Replication Composite International Diagnostic Interview (the NCS-R-CIDI), which arose out of a joint project of the World Health Organization and the former US ADAMHA. The reliability and validity of the original paper-pencil CIDI was demonstrated in both large epidemiological studies as well as in smaller validity studies.[105-108] The NCS-R-CIDI is now being used by three International Consortia as part of the World Mental Health 2000 Initiative,[109] which seeks to characterize mental health in 27 countries[110]. This newer CIDI version has been adapted for computer-assisted administration of a structured interview (it is not the CIDI-Auto which is self-administered). It generates mental health diagnoses using DSM-IV criteria. The reliability and validity of the CIDI is supported by the extensive field testing of the instrument[110], the prior validation of the paper-pencil CIDI, its reliance on ICD-10 and DSM-IV criteria, and early validation studies.[111]

The NCS-R-CIDI has notable strengths that make it appropriate for the present research. First, it permits assessment and diagnosis over three timeframes (lifetime, past year, and past month). In the present work we will administer the lifetime NCS-R-CIDI in Project 1: Efficacy and the past year version in Project 2: Longterm Outcomes during Years 1, 2 & 3. It captures continuous measures of symptoms as well as mental health symptom impact and constructs relevant to physical illness (major diseases, use of clinicians). It permits skip patterns so that individuals may be briefly screened for a disorder/condition. Finally, it is suitable for administration by trained lay interviewers. NCS-trained staff will train lay interviews in all structured interview measures. Interviewers will be subject to repeated reliability assessments throughout the study. In the present research we plan to administer the following NCS-R-CIDI components to assess mental health: Depression, Irritable Depression, Panic Disorder, Specific Phobia, Social Phobia, Agoraphobia, Generalized Anxiety Disorder, Suicidality, Alcohol Use, Tobacco Use, Neurasthenia, Obsessive-Compulsive Disorder, Worries and Unhappiness, 30-Day Symptoms, Use of Services, 30-Day Functioning, and Chronic Conditions. It is important to note that we are deleting a number of CIDI scales in the interest of reducing burden (e.g., all childhood disorder scales, gambling, demographics). Administered scales will provide information on diagnosis and dimensional information on mental and physical health, healthcare utilization, and quality of life. Our pilot research indicates that the NCS-R-CIDI should take approximately 90 minutes if there is a positive diagnosis.

We will also administer the Depression Proneness Inventory (DPI[59]), a 10-item self-report questionnaire developed to assess proneness or vulnerability to depression. Items are rated on a 7-point Likert-type scale and yield a summed score. The DPI has predicted differential response to smoking treatment in previous research.[57,112]

Physical Health

A number of different measures of physical health will be collected as part of this research. Blood tests will assess atherosclerotic risk progression (lipoprofiles, creatinine, C Reactive Protein), diabetes risk (simultaneous

fasting blood glucose and insulin, hemoglobin A1C), and diet (lipoprofile). Cardiovascular health will be assessed using tests such as Carotid Intima-Medial Thickness (Carotid-IMT) and Brachial Artery Reactivity Testing (BART) of endothelial functioning. In addition, a number of the modules from the NCS-R-CIDI will provide information regarding physical health (e.g., 30-Day Symptoms, Use of Services, 30-Day Functioning, and Chronic Conditions). Concurrent medication usage will be assessed at every study visit.

Lifestyle

Alcohol. Alcohol use will be assessed in three ways during the course of this study. Participants will provide a nightly summary of their alcohol intake using palm-top computers. Specifically, participants will be asked to report how many standard drinks (e.g., how many 12 oz. servings of beer, 5 oz. glasses of wine, and 1.5 oz. servings of spirits[113] they consumed each day. This quantity/frequency data will be collected for two weeks pre-quit and two weeks post-quit. Then, at each follow-up telephone call, participants will be asked to provide a daily summary of their alcohol intake for the 7 days preceding the call. This time frame is appropriate to allow interviewers to determine the exact number, size and type of drinks the participants consumed on each day[114] using a time-line follow-back method.[113,115] This type of assessment has been found to be more accurate than long-term retrospective data collection that asks individuals to remember back over months and years.[116] Alcohol abuse will be assessed, using the NCS-R-CIDI. As a final measure of alcohol-related problems, participants will complete the Short Inventory of Problems (SIP-2R),[117] a 15-item scale that assesses the frequency or extent to which negative consequences (physical, intra-personal, social responsibility, inter-personal, and impulse control) occurred in the last 3 months. The inter-item reliability coefficient for the full scale SIP is .89 with sub-scale coefficients ranging from .59 (impulse control) to .93 (intra-personal consequences). Combining these measures will permit tests of relations among alcohol use, consequences and smoking over time.

Diet. In this proposal, dietary factors are important outcome variables and can also serve as explanatory variables for other outcomes, such as changes in blood lipids, glucose control, endothelial dysfunction, and intima-medial thickness. For these purposes, measurement of individual food intakes (as opposed to group means) is essential. To accomplish this, we propose to assess dietary factors using three assessments. The first is a well-documented food frequency questionnaire (FFQ[118,119]), that assesses the average frequency of consumption of approximately 130 food items with specified serving sizes. The validity of the FFQ is well-established in both Caucasian and African-American populations[119-125] as is its association with various diseases.[120,124,126-141] To track shorter-term changes in diet during follow-up phone calls we will use a brief screening questionnaire (PrimeScreen) that predicts important health outcomes.[142] Finally, blood tests will be conducted to examine lipoprofiles and possibly blood carotenoids.

Exercise. Exercise will be assessed using three different approaches. First, the International Physical Activity Questionnaire (IPAQ),[143] which covers all realms of physical activity (occupational, housework, sports, etc.) will be completed at the Orientation session. This measure has been shown to have acceptable reliability (Spearman's rho = 0.80) and criterion-related validity (rho = 0.30) similar to other physical activity questionnaires.[143] Second, participants will be asked to carry a pedometer for one week before they quit to provide a baseline measure of activity. Pedometry has proven to be a very reliable and valid measure of steps walked when specific pedometer models are used.[144] Third, participants will participate in a symptom-limited exercise stress test during Visit 2 to assess overall physical fitness.

Quality of Life (QOL). While the NCS-R-CIDI will produce data relevant to health and mental health quality of life, the Quality of Life Inventory (QOLI)[145] will be used to capture QOL more generally. This measure has demonstrated good test-retest reliability and internal consistency ranging from 0.77 to 0.89 across three clinical and three non-clinical samples.[145] QOLI scores correlate positively with other measures of well-being and negatively correlated with general psychopathology, anxiety, and depression.

Stress. Stress will be measured using two different approaches. Participants will complete the Social Readjustment Rating Scale (SRRS),[146] a 34-item measure of stressful life events that require some adaptive or coping behavior. For two weeks pre-quit and two weeks post-quit participants will provide data on stressor

occurrence using EMA to capture hassles in real-time. Participants will identify stressor type (e.g., work, interpersonal, financial), severity, and how they coped with it.

Activities. Participants will be asked to complete EMA assessments regarding what types of activities they have engaged in since their last prompt. These activities will comprise: physical activity (e.g., running, walking, sports, other exercise), active engagement in work (paid/other), conversation (pleasant, neutral, other), other activities (e.g., phone use, cooking, driving, reading), inactivity (e.g., resting), eating, and drinking (alcohol, caffeine, other). These categories are not meant to be mutually exclusive. Each of these activities will then be rated on hedonic valence using a 7-point Likert scale ranging from Extremely Unpleasant to Extremely Pleasant. Individuals will also be asked to indicate with whom they engaged in the activity.

Psychosocial

Personality. Personality will be assessed using the Multidimentional Personality Questionnaire (MPQ) short form. This measure has excellent psychometric properties as a personality measure.[60]

Affect. Affect will be assessed primarily using the Positive and Negative Affect Survey (PANAS)[61]. Once treatment begins, participants will be asked to complete the 20-item paper-pencil version of the PANAS at each visit. In addition, two positive and two negative affect items, based on how the participant has felt in the last 30 minutes, will be administered four times per day via EMA.

Social Support/Network. Social support/network will be assessed using a structured interview[147-149] administered by a trained interviewer. This interview will include a set of elicitation questions that will direct the participant to list the members of their social network. Multiple elicitation questions will be used to increase the likelihood that important members of the network are not omitted due to recall errors.[150] Participants will be asked to report on several attributes of each person listed and to rate characteristics of their relationship. Information collected will include: demographics (e.g., age, gender, kin vs. friend); amount of contact; smoking and drinking patters and attitudes/social control behaviors related to the respondent's smoking cessation; and perceived qualities of the relationship (e.g., degree of satisfaction, support and conflict). The interview will be thoroughly pilot tested to ensure reproducible use. Participants who are married or living with a domestic partner will also be asked to complete the Kansas Marital Satisfaction Scale[151] to assess relationship quality.

Electronic Momentary Assessments (EMA)

EMA will be collected by use of palm-top computers and will be directed at three general targets for measurements: 1) Processes related to cessation and relapse (e.g., withdrawal symptoms, urges, negative affect, stressors, temptation events); 2) Processes thought to reflect treatment effects (e.g., withdrawal symptoms, positive affect, pleasurable activities), and 3) Processes that capture general life-style, physical and psychosocial functioning, and activities. These assessments are designed to provide insight into the factors that lead to cessation success and failure, to identify the mechanisms via which treatments work, and finally, to elucidate how cessation success and failure are related to important aspects of life functioning.

Participants will carry palmtop computers (EDs) for two weeks preceding and two weeks following a quit attempt. The proposed EMA timeframe will allow us to establish baseline levels, as well as capture important prequit phenomena, such as anticipatory affective reactions to the impending quit date (see Preliminary Study 4). This will also allow researchers to capture the first two weeks following the quit date, a time when relapse is most likely[152,153] and when withdrawal is typically at its worst.

The ED will prompt participants to complete: a Morning Report shortly after waking, a Standard Report at one random time in the late morning (2-6 hours after waking), another Standard Report at a random time in the afternoon/evening (6-10 hours after waking), and finally, an Evening Report shortly before bedtime. The Morning Report will take 2 minutes, the Standard Report will take 2-3 minutes, and the Evening Report will take 5 or fewer minutes. Select reports will include a medication prompt/enquiry to provide a daily reminder and index of medication adherence. During the post-quit period, if a participant reports smoking in a Standard Report then the Evening Report will solicit information on further attempts to quit and attitudes about quitting (e.g., quitting

self-efficacy, motivation to quit). ED questions will be written at a 6th grade reading level. See Table 4 for a schedule of EMA items.

Table 4. Measurement domains for Ecological Momentary Assessment

Morning Report	Standard report	Evening Report
Withdrawal symptoms<br>Affect<br>• Positive<br>• Negative<br>Smoking	Withdrawal symptoms<br>Affect<br>• Positive<br>• Negative<br>Stressors<br>• Type (work, interpersonal/ argument)<br>• Severity<br>• Coping response (smoking, talking to someone, distraction)<br>Smoking<br>Temptation events<br>• Present<br>• Coping response<br>Urges<br>• Present<br>• Coping response<br>Activities<br>• Type (work, social, recreation, shopping, driving, relaxation)<br>• With whom<br>• Pleasantness rating<br>Use of lozenge	Withdrawal symptoms<br>Affect<br>• Positive<br>• Negative<br>Stressors<br>• Type (work, interpersonal/ argument)<br>• Severity<br>• Coping response (smoking, talking to someone, distraction)<br>Smoking[1]<br>Temptation events<br>• Present<br>• Coping response<br>Urges<br>• Present<br>• Coping response<br>Activities<br>• Type (work, social, recreation, shopping, driving, relaxation)<br>• With whom<br>• Pleasantness rating<br>Alcohol quantity/frequency<br>Patch, bupropion, lozenge use

[1] Smoking enquiries in the Evening Report will target overall daily smoking plus smoking since the prior Standard Report.

The assessment targets above will hold for all ED assessments both prequit and postquit. Some of the assessment dimensions comprised by Table 4 are multifaceted and will require participants to complete branching responses via drop-down menus (Activities). Specifically, participants will choose items from lists of stressors, activities, temptation events, and coping styles. We have used similar assays in our prior work (Preliminary Studies 4 and 5). Withdrawal will be assessed with selected items from the Wisconsin Smoking Withdrawal Scale (WSWS),[104] and positive and negative affect will be assessed with selected items from the Positive and Negative Affect Schedule.[61] In our previous electronic diary assessments we have also successfully developed assessments for stressor types, occurrence of smoking, and temptation events (Preliminary Study 4). Some targets will be assessed anew in this research or assessed more fully.

The time frame for which data are requested will depend upon the variable assessed. For instance, participants will report on urge intensity and affect over the past 30 minutes. This will be done to ensure that the data being captured indeed reflect the current state and are not subject to various recall biases or summaries. However, participants will be asked to report on significant stressors that have occurred since the prior report in an effort to capture a more complete picture of daily events, which tend to be more objective and perhaps less variable than constructs such as affect and urges.

Participants will have the opportunity to alter the time of the Morning and Evening Reports on an ad hoc basis to accommodate special circumstances. If a prompt is missed or deferred it will be reinitiated in 30 minutes. All assessments will be prompted by a beep (none will be participant-initiated). This will be done since we believe that assessments will be frequent enough so that all major events will be captured with little temporal delay, and in our prior research we have found tremendous individual differences in likelihood of participants self-initiating a report. Thus, we believe that participant-initiated reports produce a biased sample of reports.

We plan to use only four ED prompts/day because in our previous research (Preliminary Study 4; McCarthy et al. [101]), we found that participants tolerated this relatively small number of assessments well, and yet this relatively light sampling schedule allowed us to capture important information such as individual differences in withdrawal and affective symptoms and to sensitively index the impact of smoking, detect the impact of situations and temptation events, and predict relapse vulnerability.[101] Thus, in the balance of intensive assessments vs.

reduced participant burden, we are opting to reduce burden. In addition, all EMA assessments will be thoroughly pilot-tested for burden and ease of use in order to optimize study participation.

Treatment

At Visit 2, participants will be assigned to one of the six pharmacotherapy conditions shown in Figure 1. Assignment will be random within groupings formed by sex, ethnicity, and site. The medication dosage regimens used in the study will be:

Patch: Eight weeks of nicotine patch treatment will begin on the quit day. Participants will use 21-mg nicotine patches for 4 weeks, 14-mg patches for Weeks 5 and 6 post-quit, and 7-mg patches for Weeks 7 and 8 post-quit. This treatment regimen accords with the recommendations contained in the PHS Guideline.[2] Participants will be given instructions on how to use the patch consistent with recommended use.[2] They will be told to apply one patch each morning to a non-hairy body surface between the neck and waist. Moreover, they will be encouraged to report any side effects promptly so that steps may be taken to facilitate effective medication use (e.g., use of cortisone cream for skin reactions).

Lozenge: Participants will be instructed to begin using either 2-mg or 4-mg lozenges on the quit day, based on whether they smoke their first cigarette within 30 minutes of waking. They will be told to allow lozenges to dissolve in the mouth rather than chewing or swallowing the lozenge. Participants will be told to use one lozenge every 1-2 hours during the first six weeks of treatment, using a minimum of 9 lozenges/day. Participants will be told to decrease lozenge use to one lozenge every 2-4 hours during Weeks 7-9, and then to one lozenge every 4-8 hours for Weeks 10-12. Participants will receive instructions not to use the lozenge while eating or drinking and to report promptly any troubles or side effects encountered due to lozenge use.

Bupropion XL: Bupropion XL (extended release) will be the formulation of bupropion used in this trial. The total daily dose of bupropion administered will be identical to that recommended in the PHS Guideline[2] – 150 mg per day for three days followed by 300 mg per day for 7 to 12 weeks. The only difference is that patients will take the 300 mg extended release tablet as a single dose in the morning rather than in two 150-mg divided sustained release doses. As with the other medications, participants will be briefed on common side effects (e.g., insomnia, dry mouth) and asked to report promptly any side effects encountered.

Bupropion + Lozenge/Patch + Lozenge: Each drug will be administered according to the appropriate protocols detailed above.

Placebos: Instructions and schedules for use of placebo medications will be the same as those used for the respective active medication. For instance, participants receiving a pill placebo (see Figure 1) will be told to take one pill each morning on days 5-7 pre-quit, and to take a single pill daily for eight weeks following the quit date, as in the active bupropion conditions. Placebo participants will receive pills, lozenges, and patches in the same proportions as active participants, except all medications will be placebo. Thus, one of the Placebo groups will receive placebo patches, and another will receive both placebo patches and placebo lozenges. Therefore, regardless of whether a person receives a patch, a lozenge, a pill, a patch + lozenge combination or a pill+lozenge combination, s/he will have the same likelihood of receiving an active drug or placebo (13%). Placebo treatments will be donated by the pharmaceutical company and will be identical in appearance to active drug. All participants will be asked to return all unused medication at the end of treatment.

Manipulation check. To assess the effectiveness of the placebo manipulation, at the Week 12 telephone contact we will ask participants whether they believed they were taking active or placebo medication and we will compare their responses with their actual treatment conditions. We will compare prior medication use with clinical success in all conditions. Individuals who have previously failed using a medication (e.g., nicotine patch) may be less confident if they are given that same medication as part of the study. However, it is important to note that although this is an efficacy study, the results need to be generalizable to the many smokers who have tried different medications and failed to maintain abstinence. In fact, our recent clinical trial data indicate that more than half of our study volunteers have prior experience with cessation medications. We could not complete the proposed research if we excluded all those with prior medication experience.

Counseling. Counseling will emphasize those elements of counseling shown to be efficacious in the PHS Guideline meta-analyses.[2] Therefore, counseling will provide support within sessions (intratreatment support), engineer support for the quit attempt outside the sessions (extratreatment support), and inculcate skills for coping with temptations to smoke such as stressors and smoking opportunities (problem solving/skill training). The last element will involve the participant anticipating any upcoming situations that might increase the chances of relapse. In addition, the counseling will comprise elements of effective brief interventions such as helping the individual clarify reasons/motives for quitting. We have a long history of successfully conducting such interventions[154] and believe that such components are representative of interventions employed in most intensive cessation programs. Study protocol will include a total of four counseling sessions. The choice of four 10-minute sessions was guided by findings of the PHS Guideline[2] that showed that optimal or near optimal outcomes were produced by four or more sessions and by total counseling contact time that exceeded 30 minutes. We wish to use a relatively intensive intervention in this study for two reasons. First, individuals in this study will face a considerable burden due to their study participation. We believe that a closer relationship with the treatment team will foster their adherence to the experimental protocol. Second, we intend to contrast the results obtained with an intensive psychosocial program with those obtained with the brief intervention program that will be used in Project 3: *Pharmacotherapies: Effectiveness in Primary Care.* This will allow us to determine the stability of inter-agent effects across different use contexts and populations.

Counselors will be university students supervised by licensed psychologists. Quality/fidelity assurance strategies will include: intensive training in counseling techniques and ethical conduct (30 hours over 2 weeks), mock sessions with trainees, regular supervisory review of session audiotapes, and quarterly team meetings to discuss safety, confidentiality, and fidelity to the manual. A licensed psychologist will be on call at all times should a psychiatric crisis occur (e.g., serious depression, suicide risk). We have used university students as counselors in our previous cessation trials with good success (Preliminary Studies).

Follow-up Assessment

All follow-up phone assessments (Weeks 12, 18, 24, 30, 42 & 52) will assess continuous and point-prevalence abstinence, the nature of relapse contexts (setting, stress occurrence, use of alcohol, social context and so on), use of any cessation aids, new quit attempts, alcohol use in the last 7 days, withdrawal (WSWS) and affective status (PANAS), including suicidality. In addition, experimental manipulation will be assessed at Week 12 by asking participants if they thought they were on active or placebo medication. Individuals who report 7-day point prevalence abstinence at Week 24 will be invited to come in for CO testing to biochemically confirm abstinence. All abstinent participants will be invited to return for a follow-up visit after their Week 52 follow-up phone call, as well as a random selection of relapsed participants (for Project 2: Longterm Outcomes). At this visit abstinence will be biochemically confirmed using CO and blood cotinine (cotinine < 20 ng/ml) and participants will then be asked to complete the first follow-up study battery, described in Project 2: Longterm Outcomes.

Promoting experimental participation

In our previous studies we have had excellent success in recruiting and retaining participants despite time-consuming, demanding data collection. For instance, in one study[101] participants successfully completed ten weeks of ED recording (Preliminary Study 4). We will make a special effort in the proposed research to promote long-term participation because we wish to ensure a high retention rate in our follow-up study (Project 2: Longterm Outcomes). We believe that participation will be fostered by the following: (1) provision of free state-of-the-art smoking cessation treatments, (2) complete and early disclosure of all experimental requirements/activities, (3) use of face-to-face counseling to promote a social bond between participants and study staff, (4) flexibility in scheduling that allows for study requirements to be met within participants' work and family obligations, and (5) compensation and recognition for completion of study milestones. With respect to the latter, we will compensate participants $50 for completing Visits 1 and 2, $50 for returning their EDs at Visit 6, $50 for attending a 6-month visit and $100 for returning for their 12-month follow-up visit. Compensation for time and travel for all other visits will be $25 per visit (i.e., total reimbursement of $485 for Project 1).

Data screening and development of databases

As in our previous research we will take extraordinary care to ensure the integrity of our databases. To reduce data entry errors, we will derive data from scannable forms that will be inspected for completion after the participant has returned them. In cases where forms are completed by researchers (e.g., follow-up forms), the researcher/interviewer will follow a computerized script that will detect errors of entry and incompletion. All databases will be inspected routinely for missing values and out-of-range values will be detected automatically.

Efficacy

Outcome Measures. As recommended by the SRNT Subcommittee on Abstinence Measures[155] we will use two definitions of abstinence for analyzing treatment efficacy: *point prevalence abstinence* and *prolonged abstinence.* Point prevalence abstinence will be defined as a biochemically-confirmed report of total abstinence from any tobacco use (even a single puff) for a 7-day period preceding the target follow-up day (e.g., 6-months or 12-months). Assessment of point prevalence abstinence yields a dichotomous outcome measure that can be analyzed via logistic regression and other procedures appropriate for categorical outcomes. Prolonged abstinence is similar to continuous abstinence (no smoking or other tobacco use between the quit day and a specified follow-up time-point) except that tobacco use during the first week after the quit day will not be counted as a violation of prolonged abstinence. The 1-week duration of the "grace period" reflects the fact that all pharmacotherapies should be at full strength by this time period. Abstinence assessments will permit determination of biochemically-confirmed abstinence status at specific endpoints (e.g., 6 months and 12 months) as well as when lapse/relapse occurred. Thus, prolonged abstinence yields measures that can be analyzed in logistic regression models and in survival analysis.

Participants claiming abstinence at the 6 and 12 month follow-ups must provide breath samples showing CO levels < 10 ppm and at 12 months all those claiming abstinence must supply cotinine samples ≤ 20 ng/ml. All participants will be queried as to NRT use as this could affect cotinine levels. For purposes of survival analysis, the prolonged abstinence measure will be based on a combination of self-reported relapse (any tobacco use that occurs at least once each week over two consecutive weeks, after the first week grace period) and biochemical confirmation. If self-reported abstinence is not supported by biochemical testing at a subsequent visit, then the date of relapse will be defined as the date midway between the last biochemically-confirmed date of abstinence and the date of the disconfirmed report of abstinence.

Efficacy contrast strategy. We plan to use a two-step strategy to evaluate relative treatment efficacy. In the first step, abstinence rates in each pharmacotherapy condition will be contrasted with the placebo condition to determine whether it is significantly different from the placebo condition. It should be noted that the placebo condition refers to the combined data from all five placebo conditions (i.e., placebo patch, placebo lozenge, placebo patch + lozenge, placebo bupropion, and placebo bupropion + lozenge). These data will be combined to produce an overall placebo abstinence rate that will reflect the effects of the different placebo conditions. It is possible that the various placebo conditions will vary in efficacy. However, we are interested in comparing each treatment with a "generic" placebo effect, and hence, we believe it is appropriate to compare each intervention with the performance across all placebo conditions. The performance of each placebo condition will be presented separately in publications so that readers may discern the effects of specific placebos. Also, effect sizes will be presented for contrasts of each active treatment with its respective placebo (see additional discussion of this in "Limitations and Concerns").

In the first step of efficacy testing, no correction for multiple tests will be used for the comparisons as we do not wish to delimit the number of efficacious therapies (relative to placebo) simply because we are evaluating many of them. We predict that all active treatments will be superior to placebo except for the nicotine patch.
The second, and crucial, step of efficacy testing will compare those pharmacotherapy conditions found to be significantly superior to the placebo condition with one another. This will leave a total of four active treatments to be compared in a pairwise fashion to determine relative superiority. For the six pairwise comparisons, we plan to use a correction to alpha in order to reduce the likelihood of making a Type I decision error. We propose using Holm-adjusted p-values[156] that will provide more power than the Bonferroni correction.[157] Estimated point prevalence abstinence rates for each treatment condition are presented in Table 5.

Dichotomous outcome data (point prevalence abstinence and prolonged abstinence) at the end of treatment (EOT), 6 months, and 12 months will be tested using logistic regression models using the two-step contrast strategy described above. These endpoints will also be analyzed in longitudinal models using generalized estimating equations (GEE[158,159]), which allows for modeling covariance structures and autocorrelation across repeated observations.[160] Survival analysis will be used to analyze prolonged abstinence. Such analyses will also be used to model the relations of targeted genotypes with outcomes.[161]

In addition to cessation outcomes, we will contrast pharmacotherapies on side effects, and their ability to suppress withdrawal symptoms (elevation, trajectory, variability) and weight gain. Multilevel modeling will be used in the latter contrasts as in our previous research (see Preliminary Studies 4 and 5).[63,64,101,162,163] We will conduct contrasts both with and without covariates so that the generalizability of our findings may be gauged. Control variables will include gender, age, prior use of cessation medications, study site, dependence level, and depression history.

Tests of Moderation: Constructing a Smoking Cessation Treatment Algorithm

In order to develop useful and valid decision rules for assigning smokers to treatments, we will explore whether important characteristics of smokers are related to their response to treatment, whether these associations are reliable across two large data sets (Projects 1:Efficacy & 3: Effectiveness), and whether they can be used to generate a treatment algorithm that is simple for clinicians to use successfully in treatment settings.

A treatment algorithm will be useful to the extent that the predictors are readily available or cheaply gathered, and to the extent that they reliably and validly index optimal patient responses to treatments across different treatment contexts. Based upon these considerations, the following variables will be explored as potential decision indicators: gender, dependence level, depression proneness/history, and negative affect. (Other variables will also be screened as mentioned below.) All of these variables can be assessed relatively quickly/cheaply (albeit imperfectly), and all have been shown in past research to predict differential response to treatment.[13,47,56, 57] Dependence level will be measured with the WI-PRISM (Preliminary Study 2) and FTND. In addition, we plan to test depression proneness/history in several ways: (1) using the three best questions from the Depression Proneness Inventory, (2) using a single item of prior depression history, and (3) using depression diagnosis or relevant questions from the structured psychiatric interview. The latter will not be amenable to routine clinical use, but it will allow us to determine agreement with the other two measures.

Although logistic regression is frequently used in attempts to develop treatment allocation strategies or algorithms,[164-166] conventional logistic regression models are limited by difficulty in the interpretation of model coefficients, especially in the presence of collinearity, nonlinearity, and interactions. An alternative algorithm development approach that addresses these limitations is decision tree analysis.[165,167-169] Decision tree analysis comprises a variety of techniques that generally involve a form of binary recursive partitioning of individuals to optimize a specified outcome or decision. In general, tree analysis uses available splitting (or predictor) variables (e.g., dependence level, gender, depression history) to separate individuals recursively so as to produce an optimal decision for subsets of individuals with particular characteristics. The successive partitioning of a group of individuals involves starting with a root node consisting of all individuals and then subdividing the root node into leaf nodes based on the values of one or more splitting variables (e.g., gender).

We plan to use two types of decision tree analysis to develop treatment algorithms. The first type is logistic regression tree analysis. In this analysis, each leaf node yields a linear logistic regression model in addition to the number of sample cases in each class. One advantage of this type of decision tree analysis is that it can estimate the probability that the predicted class will be correct for a future case. These predicted probabilities can be used to establish optimal cutpoints for continuous predictor variables (e.g., nicotine dependence). Chan and Loh[166] have developed a logistic regression tree analysis algorithm and computer program called LOTUS (Logistic regression Tree with Unbiased Selection) that we will use.

The second decision tree analytic strategy that we will use is similar to classification and regression tree (CART) analysis.[167] The approach we will use[170] improves on CART by providing a variable selection technique with negligible bias, better handling of missing data, and the ability to include categorical predictor variables with

many categories. This improved classification tree analysis algorithm is called QUEST (Quick, Unbiased and Efficient Statistical Tree).[170] The main difference between the LOTUS algorithm and the QUEST algorithm is that each leaf node in QUEST is a binary predicted class (e.g., male vs. female) with the predicted proportions of sample cases in each class. Thus, QUEST does not use statistical models in its leaf nodes as LOTUS does. One advantage of QUEST is that the user can specify misclassification costs to determine relative accuracy of the treatment algorithm.

As noted above, both the LOTUS and QUEST algorithms and programs were developed by Wei-Yin Loh, Ph.D. and colleagues.[166,170] Dr. Loh, a Professor of Statistics at the University of Wisconsin-Madison and a co-investigator on this grant, will assist in the treatment algorithm analyses. One algorithm would comprise only OTC interventions (e.g., the nicotine lozenge). Here the smoker himself/herself could be advised (perhaps via media) which OTC treatments work best for which types of smokers (e.g., males vs. females, low vs. high dependence severity). A second algorithm would involve only monotherapies, while the final algorithm could involve any of the therapies studied in this research including dual pharmacotherapies. It is important to note that we will examine the role of both 2- and 4-mg lozenges in the algorithms to explore whether the algorithm depends upon a particular lozenge dose (which will be somewhat confounded with dependence level).

The effectiveness of algorithms will be contrasted not only with each other, but also with different comparison models. An *optimal monotherapy model* comparison involves comparing efficacy rates produced by an algorithm with rates produced by the best single treatment. For instance, the efficacy rates predicted via application of the OTC algorithm might be compared with the success rate of the single best monotherapy or the single best treatment among all treatments. The *mean pharmacotherapy* model will compare the efficacy rates prognosticated based upon use of an algorithm with the average efficacy across all the pharmacotherapies for all participants. Finally, the *placebo model* will compare the algorithm efficacy with the average efficacy across the various placebo conditions. In discussions of the various algorithms, and in making treatment recommendations, we will also review costs and side effects so that these factors may be weighted relative to abstinence effects per se.

One important concern with respect to decision rules is their generalizability. We will attempt to build-in generalizability in several ways: (1) The sample is large and diverse. (2) Decision rules will be constructed with an eye to parsimony and replicability.[171,172] Thus, unless a decision rule is easy to use and clearly supported by statistical and clinical significance, the rule will not be invoked. (3) We will avoid higher-order models in which treatment decisions at a single point or node depends upon status of more than two indicators. Higher-order interactions tend to be less stable across populations.[173] (4) The stability of algorithms will be examined across the Madison and Milwaukee sites, and across ethnicities using bootstrapping procedures.[174,175] (5) Finally, all the indicators will be assessed in Project 3: Effectiveness to allow for validation. This will allow us to examine how well the algorithms work in a context highly representative of the clinical use of these agents. Once algorithms have been tested in the two populations they may be altered in order to achieve the broadest clinical utility. (The sample used in the present research will not be split into derivation and validation samples since research suggests that decision rules are most stable when generated with the largest initial population).[175,176]

Another use of LOTUS and QUEST models will be to test indicators/splitting variables that are not already suggested by the extant literature. Such techniques can efficiently scan multiple predictor variables. Therefore, variables such as ethnicity, alcohol use/abuse history, prior longest abstinence period, individual WISDM-68 subscales, or genotype will be entered into models to guide future research and theory.

Tests of Mediation

Mediational tests will be guided by theoretical models of treatment effects. As noted in the Background and Significance section, potential mediators are: withdrawal symptoms (including urges, negative affect), positive affect, stress reactivity, reactivity to temptation events (e.g., seeing other smokers), expectations of smoking reinforcement, reactivity to lapse cigarettes, and capacity to experience pleasure due to nonpharmacologic reinforcers. It is important to note that the above potential mediators will be carefully characterized so that we can sensitively assess the effects of treatments on these variables prior to evaluating a potential mediational role, as we have done in the past (Preliminary Study 5). For instance, withdrawal symptoms will be evaluated in

terms of their different profile dimensions (e.g., average or initial level, growth over time).[63,64,162] Moreover, because mediational relations are highly compromised by unreliability of the mediator,[70] to the extent possible, mediators will be represented by latent variables (e.g., reflecting a growth parameter).

Figure 2. Basic Mediational Model

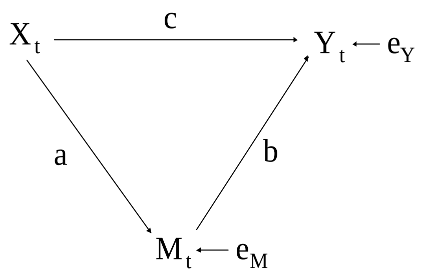

Figure 2 depicts the basic model used to test mediational relations. In this Figure X_t represents the primary causal variable at time t (e.g., treatment condition), M_t represents the mediator, while Y_t represents the dependent variable (e.g., survival duration). The e_M and e_Y variables represent sources of unexplained variance in the mediator and dependent variable. A mediational relation is one in which X has a direct effect on M, M has a direct effect on Y controlling for X, and the relation of X and Y (path c) is meaningfully reduced when the indirect paths a and b are included in the model.[177] To estimate the strength of the mediation effect hypothesized, we assume a partial mediation model and note that extent of mediation can be indexed by ab/(c + ab)[178] with the standard error of the indirect effect estimated via the large sample test of Sobel or via bootstrapping depending on size of sample or subsample tested.[179,180]

While it would be desirable to use a longitudinal mediational design[70] with multiple waves of X variables, the fact that X (pharmacotherapy condition) will remain constant across the study precludes more complex longitudinal designs. (We will not code level of lozenge use since this may share nonrecursive relations with M). However, we will take advantage of the prospective nature of the design by preserving temporal ordering of events in the analytic plan. Thus, X will represent pharmacotherapy condition, which temporally precedes other variables and is not vulnerable to reciprocal influence by other variables. M will be modeled across different time spans and types of measures. For instance, withdrawal elevation could be modeled as elevation (mean level) across the first 1, 2 or 4 weeks post-quit or as a trajectory (e.g., linear slope) across diverse time spans. In addition, Y will be selected to reflect survival functions that post-date the time span captured in M. Of course, smoking (i.e., lapsing) may occur during the time span captured by M. We will explore how post-quit smoking affects M by covarying out contemporaneous smoking from the mediator. Moreover, it is likely that the effects of treatments are not mediated by any single variable, but rather influence outcomes via multiple actions. Thus, we will explore multiple-mediator models. This will help ensure that the mediational model is not misspecified by omitting important mediational paths.[179] Because individual mediational effects are not assumed to be large, we will test for mediation even in the absence of significant X-Y relations.[179,181].

In tests of mediation, each active pharmacotherapy condition will be contrasted with the placebo condition in an *upper level mediational model.*[182]. These analyses will reveal whether specific pharmacotherapy effects on outcome are mediated by particular M(s). SEM analyses can be used to determine whether significant mediational relations differ across the different pharmacotherapies. Multigroup SEM analyses can also be used to determine whether mediation differs as a function of individual differences such as gender or level of nicotine dependence, since moderation of mediational effects can also result in model misspecification.[179] This is important because different mechanisms may account for treatment effects in different populations.

Lower level mediational models will also be tested.[183] For instance, we will test whether a lower level variable such as a genotype is related to subsequent smoking likelihood, and if it is, whether this relation is mediated by a variable such as withdrawal severity. Tests of such mediational paths will not only reveal important information about relapse, but multi-group models may reveal whether particular pharmacotherapies work via such lower level paths (e.g., by reducing withdrawal dimensions).

Attrition

We will adhere to the intent-to-treat principle in this research so that participants who drop out of treatment or follow-up will be counted as smoking. However, attrition is important since analyses such as those examining mediational effects require continued participation. We have achieved high retention during follow-up in the past (Preliminary Studies). The present study will use greater incentives for participation and provide more information about study requirements than our past research but we expect 15% attrition during treatment. It is

important to note that the multi-level modeling strategies to be used in analyzing much of the EMA data in the proposed study are compatible with moderate levels of missing data.[184] Finally, participants will not be randomized to a treatment condition (and therefore entered into the intent-to-treat analyses) until Visit 2. Therefore, if a participant withdraws from the study before any treatment has been provided, ostensibly due to the assessment burden, the data from that participant will not be entered into analyses. However, we will monitor early attrition in order to assess for differential attrition and bias.

Power

We will focus on long-term abstinence rates in estimating power. Because numerous studies contain data on efficacy, and because these studies yield a great range of efficacy rates, we will consult meta-analyses to help us generate effect size estimates. An analysis of 28 placebo-patch study arms in the PHS Guideline[2] revealed a 6-month point-prevalence outcome of 10% (that Guideline focused on 6-month outcomes). Placebo arms for other pharmacotherapies in the PHS Guideline generated abstinence rates ranging from 10-17%, with most rates in the 10-13% range. Many of these studies involved fairly intensive counseling or frequent clinic visits similar to the proposed research. The Cochrane report[4] studies showed that placebo arms comprised by nicotine patch studies generated an overall abstinence rate of 8%. Since the Cochrane reports tended to use 12-month "sustained abstinence rates," the Cochrane rates are probably too conservative for 6-month estimates. Based upon this wealth of data on placebo group performance we estimate that 12% of the placebo participants will be abstinent at the 6-month follow-up.

Although meta-analyses suggest that nicotine patch treatment should roughly double abstinence rates relative to placebo (OR 1.7-1.9)[2,4,185], we are estimating a lower 6-month rate in Table 5 because in our recent research[11], patch treatment produced an OR relative to placebo of only 1.2. Second, in one of our recent studies, the patch produced a 6-month abstinence rate of only 14%.[39] Also, a recent meta-analysis of 25 patch studies suggests that the efficacy of the nicotine patch has declined steeply over the past 10 years relative to declines in the performance of placebo controls[24] (also see Fox et al.[186], for supportive evidence). Based upon such evidence, we believe that the patch will produce an OR relative to placebo of only about 1.4. Therefore, we project a 6-month abstinence rate of about 17% for the patch condition. We believe that a 5% increment in efficacy (17% abstinent in the nicotine patch group compared to 12% in the placebo group) is not clinically significant, and we have not powered this study to detect it.

The PHS Guideline revealed an OR for bupropion vs. placebo of 2.1. The Cochrane meta-analysis yielded an OR of 2.[29] Based upon these two consistent characterizations, we expect an OR of around 2 in the proposed research and therefore propose an estimated 6-month abstinence rate of 24% (see Table 5). The best data for estimating the lozenge efficacy is the Shiffman trial,[25] which yielded an OR of about 2.3. This yields a 6-month abstinence rate of about 27% (see Table 5).

Table 5. Estimated abstinence rates at 6 months.

Placebo	Patch	Lozenge	Patch + Lozenge	Bupropion	Bupropion + Lozenge
12%	17%	27%	40%	24%	42%

The PHS Guideline meta-analysis yielded an OR of 1.9 for NRT combinations relative to monotherapy, while the Cochrane report generated an OR of 1.6.[4] These would yield abstinence estimates of 51% and 41 % relative to the lozenge per se. (Because we do not expect the nicotine patch to produce high levels of abstinence in this research, we do not believe that it is very meaningful to demonstrate an effect relative to the patch. Therefore, we are powering this study to show an effect relative to the bupropion and lozenge monotherapies). Consistent with the PHS Guideline, we expect the combination therapies to yield ORs of about 1.5. While some recent trials of combination therapies have not shown such strong effects, none of these used the lozenge. An OR of about 1.5 would produce 6-month point prevalence abstinence rates close to 40%, and we give a slight edge to the lozenge + bupropion combination due to the unique mechanisms of action of these two agents. See Table 5 for estimated abstinence rates at 6 months.

In order to simplify power estimates, we will assume use of logistic regression at 6-month follow-up. Our goal in powering this study is to balance Type I and Type II errors (cf., Westfall et al.[157]). The proposed study has six arms including the placebo condition. Step 1 involves five uncorrected tests in which each active arm is

contrasted with the placebo condition. Step 2 involves tests among those agents found to differ from the placebo condition. We predict that all of the active treatments except for the nicotine patch will be superior to the placebo and the study is adequately powered to detect these effects even using the Holm adjustments to alpha (power > .91 except for the control-patch comparison).

Table 6. Power for the comparison of 6-month point prevalence abstinence rates of active treatments that are predicted to differ from placebo.

Comparison	Differential	Power (α=.05, uncorrected for multiple tests)	Power (Holm adjusted p-values)
Lozenge vs. Patch + Lozenge	13%	0.89	0.78
Lozenge vs. Lozenge + Bupropion	15%	0.95	0.87
Bupropion vs. Patch + Lozenge	16%	0.98	0.92
Bupropion vs. Bupropion + Lozenge	18%	0.99	0.96

The second step of efficacy testing involves comparisons among the active agents that differ significantly from the placebo control. We anticipate that four agents will differ from the control condition. Table 6 provides power estimates for uncorrected and Holm-corrected tests of the four active treatments that we predict will be superior to placebo. We predict that the dual pharmacotherepies (patch + lozenge; bupropion + lozenge) will be superior to the monotherapies (lozenge alone, bupropion alone) and the study is adequately powered to detect these predicted effects. Although we are predicting that bupropion + lozenge will yield a higher abstinence rate than the patch + lozenge (42% versus 40%, respectively), we do not believe that this is a clinically significant increment and the study is not powered to detect this effect.

Limitations and Concerns

One concern is that the different agents will have different targeted durations of use. For instance, the lozenge will be made available to participants for 12 weeks postquit, while bupropion and patches will be available for 8 weeks postquit. While we could have attempted to impose a standard duration of use across agents, we decided to compare drugs with one another when their use is consistent with both their package inserts and the PHS Guideline.[2]

Another concern is that these treatments will all be evaluated with only a single level of counseling. It is possible that relative efficacies might differ if we employed a different intensity or type of psychosocial intervention. We do not consider this a serious concern as most evidence suggests that pharmacotherapy efficacy does not interact with intensity of psychosocial intervention/counseling.[1,2] Second, since these same pharmacotherapies will be used in Project 3: Effectiveness with very modest adjuvant intervention, we will obtain a good sense of their relative efficacy across two very different levels of psychosocial intervention. Finally, pragmatic considerations prevented our examining efficacy across a range of adjuvant treatments.

Another concern is that we do not plan to have all individuals use two agents (whether active or placebo) in all conditions. We could have designed the study such that all participants receiving active therapies simultaneously used a placebo agent (e.g., active patch + placebo bupropion), and all placebo participants received two placebos (e.g., placebo bupropion + placebo lozenge). We decided not to use two agents in all conditions for the following reasons: (1) This would have added great expense to an already expensive study. (2) This would have created more burden for participants in a study where burden is somewhat high. (3) This would have been extremely difficult to employ in Project 3: Effectiveness and would compromise the purpose of that study because it would not have reflected how these agents would be used in clinical settings. If we used dual agents in this study but not in the effectiveness study, this would have created a troublesome confound across those two studies preventing straightforward comparison. (4) There are several recent trials showing that dual treatments (all involving bupropion + NRT) do not significantly enhance outcomes (e.g., Jorenby et al.[11]). This suggests that a placebo effect from the use of two agents is not a potent influence on outcomes.

The placebo condition is an amalgam of different placebo subtypes (e.g., placebo patch, placebo lozenge). This is a limitation in that each active agent will not be contrasted with its specific placebo control. Rather, active agents will be contrasted in significance tests with the whole placebo condition because: (1) it would be prohibitive in cost to contrast each agent with its own control with adequate power; (2) participants assigned to each type of agent have an equal chance of getting placebo; and (3) all active agents will be compared with a

common benchmark (the same index of placebo effects). Finally, a major focus of the study is to determine relative efficacies of the active pharmacotherapies. Demonstrating efficacy relative to placebo, in our view, will be a relatively low hurdle of some of the agents (e.g., lozenge, patch + lozenge). The more crucial and clinically important tests will involve tests of relative efficacy among the active agents. However, if reviewers of this application seriously question this placebo strategy, we will assemble design experts to consider alternatives, especially any recommended by reviewers.

Another concern is that we do not propose using an active placebo. An active placebo might change participants' mood or sensations and therefore affect abstinence rates – either increasing or decreasing them. Therefore, we decided that an active placebo should be used only if a passive placebo were also used and the size of the study precluded the use of two placebo conditions.

One final limitation is that we will include individuals who have previously used some of the pharmacotherapies being tested. Therefore, it is possible that an individual may have sufficient experience with an agent (e.g., patch) to know whether s/he is in the active condition. In addition, expectations of treatment efficacy for the individual may vary by previous experience. Since eliminating people with prior use of cessation medications would prevent completion of the proposed work, we decided to examine and control such relations statistically.

Connections with TTURC Theme and Companion Research Proposals

One of the exciting features of the proposed UW TTURC research program, *Tobacco Dependence: Treatment and Outcomes*, is the integration of measures, theories, and techniques generated by different disciplines in an effort to advance our understanding of tobacco cessation. The proposed research will help us better understand the process of quitting smoking and the outcomes of attempting to quit smoking, both for the smoker (Project 2: Longterm Outcomes) and for broader society (Project 4: Healthcare Utilization). Project 1: Efficacy, will compare the efficacies of multiple pharmacotherapies for smoking cessation, reveal treatment mechanisms and generate a treatment algorithm. In a complementary fashion, Project 3: Effectiveness, will use the same cessation pharmacotherapies, but in a primary care setting. Thus, we will capture both efficacy and effectiveness data within the proposed studies. These two studies will both shed light on how well pharmacotherapies work, and allow researchers to develop effective treatment algorithms. However, Project 1 will produce more information on how treatments work and the genetic basis of nicotine dependence, and Project 3 will have more immediate relevance to real-world use of cessation medications. Together, Projects 1 & 2 will use integrated assessments of medical, physiological, psychological, social, and genetic factors to reveal how post-treatment abstinence outcomes yield downstream effects on psychosocial and health endpoints. Thus Project 1 will concentrate on how well treatments work, and for whom they work, while Project 2 will examine global impacts of treatment and quitting. In sum, this research program will use diverse study designs and methods ranging from a highly controlled efficacy study vs. a generalizable primary care effectiveness study, will collect both randomized control and longitudinal data, and integrate assays across a wide variety of disciplines - - from genetic to medical, to social mapping, to policy. These complementary research designs and measures promise to yield a deeper understanding of how well, by what mechanisms, and for whom, smoking cessation pharmacotherapies work - - as well as characterizing the long-term consequences of quitting and relapsing.

E. Human Subjects

Risks to the Subjects

Human Subjects Involvement and Characteristics: The 1520 subjects will be smokers 18 years of age or older. Inclusion criteria will be: (1) smoking at least 10 cigarettes/day (on average) for the previous 6 months, (2) a breath sample with a CO level > 9 ppm, (3) being motivated to quit, (4) able to read and write English, (5) agree to respond to EMA prompts throughout the day, (6) plan to remain in the treatment catchment area for at least 12 months after the initiation of treatment, (7) no medical contraindications for use of bupropion (e.g., no uncontrolled hypertension, history of bipolar illness, recent myocardial infarction, recent heavy alcohol use, seizure history, or use of MAO inhibitors or other contraindicated medications), (8) no risk of suicide, (9) no other

member of the household is participating, and (10) women are not pregnant and agree to prevent pregnancy during the course of treatment. There will be no restrictions on participation based on sex or race.

Sources of Materials: Subjects in this program will provide data to the program for the express purpose of research. Data will consist of answers to questionnaires and interviews assessing smoking history, demographics, nicotine dependence, personality, affect and affective vulnerability, psychopathology, stress, social networks, physical activity and food intake, vital signs (including blood pressure, temperature, heart rate, body weight and height), breath samples to permit determination of carbon monoxide, blood samples to permit assays for cotinine, DNA, lipoprofiles, creatinine, simultaneous fasting blood glucose and insulin, hemoglobin A1C, blood carotenoids, and c-reactive protein. Carbon monoxide and cotinine assays reflect smoking status. Data regarding lung and cardiovascular function will be collected using specific medical procedures, including ultrasound Carotid Intima-Medial Thickness test (CIMT), Ultrasound Brachial Artery Reactivity Test (UBART) of endothelial functioning, and an exercise stress test.

Potential Risks: Risks associated with this research are judged to be minimal. The only significant risks are those posed by the various pharmacotherapies (i.e., bupropion, nicotine patch, and nicotine lozenge), smoking withdrawal per se and the exercise stress test. With respect to the pharmacotherapies, participants will be made aware of the common side effects before they consent to participate in the study. It should be noted that both nicotine patch and nicotine lozenge are available over the counter, suggesting minimal risk. The nicotine patch has very few side effects, but up to 50% of participants may have a local skin reaction. The most likely side effects associated with the nicotine lozenge are heartburn, hiccup, nausea, upper respiratory tract infections, coughing and sore throat. Bupropion has been shown to be quite safe in large-scale clinical trials for smoking cessation. The most likely risks associated with bupropion therapy are insomnia and dry mouth. Other risks, such as seizure, are much less likely in medically screened subjects. Participants will be told of these risks and their likelihood before consenting to participate. Smoking withdrawal is associated with a number of unpleasant symptoms, such as sleep disturbance, hunger, craving, and negative mood. Most smokers have tried to quit in the past and are familiar with these phenomena. Though unpleasant, smoking withdrawal symptoms pose no acute health risk. Participants will be informed about the likely effects of smoking withdrawal. The most substantial risk associated with the exercise stress test is the risk of a coronary event. However, the risk of a coronary event in medically supervised exercise treadmill tests is less than 1 per 5000-10,000 tests, and events are usually minor in nature (e.g., angina, dizziness) due to the test being supervised. Serious coronary or other events are rare due to the monitoring and the cessation of the test in the presence of ECG changes, any arrhythmias, abnormal symptoms, etc. Medical supervision will be available for all tests. One final potential risk includes the fact that participants will be informed about any urgent medical condition that they may have previously been unaware of (e.g., advanced atherosclerosis that might lead to stroke). The other medical assessments pose little or no risk. Brachial and carotid artery ultrasound studies are safe, noninvasive, and without discomfort. There are no known adverse physiologic effects related to diagnostic medical ultrasound. Subjects who elect not to participate in this research program, or are eliminated due to screening failure, will be given a list of alternative smoking cessation programs.

Adequacy of Protection Against Risks

Recruitment and Informed Consent: As in our previous research, participants will be recruited via advertisements in newspapers, radio, television, billboards, and other media. In addition, free media (e.g., press conferences) will be utilized. Participants will be recruited in both Madison, WI and Milwaukee, WI. Advertisements and publicity will contain a phone number for interested individuals to call to contact study personnel. After calling this number, participants will undergo initial phone screening to rule out those with clear contraindications. The study will be briefly described, questions answered, and potentially qualifying individuals will be invited to attend an Orientation Session. At the Orientation Session the general requirements for participation will be reviewed (e.g., session attendance, need for follow-up, participation in assessments, participation in the proposed follow-up cohort study – Project 2: Longterm Outcomes). In addition, participants will be informed of the nature of the treatments involved. They will be told that five different pharmacotherapy conditions will be studied and that participants will receive either one of those treatments or a placebo medication. In addition, participants will be told that everyone will receive counseling designed to aid them in their cessation attempt. After answering additional questions about research participation and treatment, participants will be asked to read and sign a

consent form as well as an appropriate HIPAA form. Subjects will also be given copies of the consent form and HIPAA form to take home. After signing a consent agreement, potential subjects will be scheduled for an individual screening session, where previously assessed inclusion/exclusion criteria will be confirmed and other inclusion/exclusion criteria will be assessed. Individuals will be encouraged to ask any further questions about the study protocol throughout the study.

Protection Against Risk: Subjects will be screened to ensure that they are medically appropriate for nicotine replacement therapy and bupropion, and in-person physician evaluation will be available if any questions arise. With respect to the baseline treadmill stress electrocardiogram tests, participants will participate only if they do not have contraindications and are clinically stable. Participants will be screened prior to the test by trained physiologists for absolute and relative contraindications to stress testing, using physician reviewed protocols. Participants will have documented clearance by physicians for testing prior to the test, and informed consent will be obtained. Twelve-lead ECGs will be reviewed by a physician prior to the test to ensure that the participant does not have ECG or clinical contraindications to the test. Test termination criteria are also standard for stress testing, including the development of chest pain, more than 1 mm. of horizontal or downsloping ST segment depression in standard leads and other usual ECG criteria in addition to clinical criteria including abnormal hemodynamics, serious arrhythmias, abnormal symptoms, significant dyspnea, or abnormal oxygen saturation measurements. Subjects will be given a telephone number to contact the study physician if they experience serious adverse events. Adverse events (AEs) and serious adverse events (SAEs) will be assessed at each study visit and study staff will verify that all adverse effects are being addressed either by the subject, his/her regular physician, or offer to have the study physician address the concern. In addition, suicidality will be assessed at every visit and follow-up contact. Individuals who report any suicidal ideation will be contacted by a licensed psychologist who will assess the level of risk and provide referrals as needed. Confidentiality of subject data and information will be accomplished by using subject numbers as unique identifiers, allowing us to keep subject data separate from identifying information. No publications or presentations resulting from this research program will contain any identifying information about individual participants.

<u>Potential Benefits of the Proposed Research to the Subjects and Others</u>
The potential benefits for smokers participating in this study include the chance to receive free smoking cessation pharmacotherapy and smoking cessation counseling, both of which double a smoker's odds of quitting.[2] The risks of this research are chiefly associated with the provision of bupropion as one of the pharmacotherapies. These risks are reasonable because this medication has been shown to be safe in numerous large clinical trials using medical screening criteria similar to those that will be used in this study. Because the health risks associated with continued smoking dramatically outweigh those associated with bupropion use, and because it is likely that many subjects will successfully quit smoking as a result of their participation in this research, the potential risks to subjects are acceptable compared to the potential benefits. The availability of consultation with the research program, including physician consultation, also decreases the likelihood of adverse consequences from bupropion use. In addition, this research has the potential to provide treatment algorithms for clinicians trying to help patients quit smoking. This could result in more efficient provision of maximally efficacious treatment for smokers.

<u>Importance of the Knowledge to be Gained</u>
This is the first research study to provide a head-to-head comparison of numerous smoking cessation pharmacotherapies, including combination pharmacotherapies. The data from this study may allow researchers and clinicians to determine which agents are most efficacious overall. In addition, the proposed development of treatment algorithms will allow clinicians and researchers to tailor smoking cessation pharmacotherapy so that each smokers is given the best, most efficient pharmacotherapy for his/her quit attempt. Finally, this research may help elucidate the mechanisms of action and interaction for the various pharmacotherapies, which may prompt the development of new pharmacotherapies. Given the limited risks of bupropion and nicotine replacement therapies, and the rigorous pre-treatment screening and the availability of both physicians and psychologists to address any adverse effects, we believe that the potential risks involved in participating in the study are outweighed by the benefits to both society and the individual.

Participation of Women

Based on previous research conducted in Madison and Milwaukee, we project that 855 (approximately 56%) participants will be women. Because women are well represented in the two communities, there is no need for a specific recruitment strategy. The research plan does include analyses that will evaluate data by gender. Differential response to treatment based on gender is one of the central hypotheses of the research. See targeted enrollment table.

Participation of Minorities

Previous work in Madison and Milwaukee indicates that the proposed recruitment strategies will result in a diverse mix of participants. We anticipate approximately 17% African-American participants, with another 2% split between Native American/Alaska Native and Asian participants. Approximately 2% of participants will have Hispanic heritage. The research plan also includes some analyses that will evaluate data by minority status. Given the demographics of the communities where recruitment is occurring and the size of the study, it is anticipated that the study will recruit sufficient African American participants to allow these data to be separately evaluated. See targeted enrollment table.

Participation of Children

Children under the age of 18 will not be included in the proposed clinical research because none of the medications being used in the proposed study have been approved by the FDA for use by children under that age. Children between 18 and 21 will be eligible to participate. In the state of Wisconsin, persons of this age are allowed to consent to their own participation in clinical research.

Data and Safety Monitoring Plan

The following Data Safety and Monitoring Plan (DSMP) pertains to all research that is supported under the National Cancer Institute/National Institute on Drug Abuse/National Institute on Alcohol Abuse and Alcoholism Transdisciplinary Tobacco Use Research Center (TTURC) Award. This plan comprises not only the research conducted directly by the University of Wisconsin Center for Tobacco Research and Intervention (CTRI) researchers, but also research conducted by other investigators who are supported by TTURC funds. All investigators must agree to comply with the procedures outlined in this DSMP. This DSMP does not reduce any investigator's obligation to comply with the requirements of the Institutional Review Board (IRB) at his/her home institution or the IRB of any collaborating organizations.

Monitoring the progress of trials and the safety of participants. Each Project Principal Investigator is responsible for routine monitoring of the trial's progress. This includes scheduled biweekly meetings with study staff and review of written documentation. Data that are reviewed at these meetings include the number and type of participants enrolled, the number and reasons for exclusions from enrollment, the number treated and the stage of treatment, summary of adverse events, individual review of serious adverse events and study participation and outcome data.

Additionally, each Project Principal Investigator is responsible for briefing the TTURC Principal Investigator on the trial's progress on a regularly scheduled basis (typically biweekly). As data become available, the Data Manager, Project Principal Investigator, and TTURC Principal Investigator will review the data on a regularly scheduled basis (typically biweekly) to determine progress.

To facilitate participant safety, study participants must meet study inclusion criteria. Once enrolled in the trial, follow-up protocols will assess for the presence of adverse events. Should either excessive risk to study participants and/or lack of measurable benefit to study participants be determined, the study will be stopped and all participants notified in a manner appropriate to the nature of the risk and/or lack of benefit.

Plans for assuring compliance with requirements regarding the reporting of adverse events. This DSMP requires that investigators notify the National Cancer Institute (NCI) and the University of Wisconsin IRB of the occurrence of any serious adverse event (SAE), or any adverse event (AE) which is severe, unexpected, and possibly related to study medication or protocol. Such notification must occur within five days of investigators becoming

aware of the event.

If the study in question involves a pharmaceutical agent, and the serious adverse event might be related to drug use, both the Food and Drug Administration and the manufacturer will also be notified with five days of investigators becoming aware of the event. Examples of serious adverse events would be untoward medical or treatment occurrences that result in death, are life-threatening, require hospitalization or prolonging of existing hospitalization, create persistent or significant disability/incapacity, or involve congenital abnormality/birth defects. Unanticipated adverse events would include less serious problems that merit reporting because they are severe, unexpected, and possibly related to study participation. Any serious adverse event (SAE) will be queried and reported even if it appears that the serious adverse event is unrelated to treatment participation.

Not only will investigators report any serious adverse events (whether or not they are unanticipated), but the Principal Investigator of each project will also be responsible for the accurate documentation, investigation and follow-up of all study-related adverse events.

Adverse event assessment, recording, reporting and investigation will be accomplished through staff training, structured or standardized assessments of untoward occurrences/events, and regular monitoring by study investigators. The Project Principal Investigator and the TTURC Principal Investigators have ultimate responsibility for ensuring that serious adverse events are detected and reported in a timely manner. Additionally, the IRB will receive an annual report of all serious adverse events and adverse events meeting the criteria listed above.

Plans for assuring that any action resulting in a temporary or permanent suspension of an NCI-funded clinical trial is reported to the NCI grant program director responsible for the grant. The NCI grant program director will be notified within five days if the TTURC Principal Investigator deems it necessary to suspend a clinical trial. In the case of a temporary suspension, the TTURC Principal Investigator and Project Principal Investigator will develop a plan for continuation of the study and discuss this plan with the NCI grant program director in a reasonable time frame.

Plans for assuring data accuracy and confidentiality and protocol compliance. The Data Manager and Project Principal Investigators will develop plans for assuring data accuracy and protocol compliance, which will be reviewed and approved by the TTURC Principal Investigator. Such plans will include data verification and protocol compliance checks. The Data Manager and Project Principal Investigator shall also be responsible for ensuring that the data for the project are securely stored, that storage is in compliance with University and federal regulations and that no unauthorized persons have access (electronic or physical) to any participant-identifiable data. In addition, all HIPAA regulations and guidelines will be followed.

F. Vertebrate Animals

None.

G. Literature Cited

1. Fiore MC, Bailey WC, Cohen SJ: Smoking cessation: Clinical practice guideline No. 18. Rockville, MD, U.S. Department of Health and Human Services, Public Health Service, Agency for Health Care Policy and Research, 1996
2. Fiore MC, Bailey WC, Cohen SJ: Treating tobacco use and dependence: Clinical Practice Guideline. Rockville, MD, U.S. Department of Health and Human Services, U.S. Public Health Service, 2000
3. Rigotti NA: Treatment of tobacco use and dependence. New England Journal of Medicine 2002, 346:506-512
4. Silagy C, Lancaster T, Stead I, Mant D, Fowler G: Nicotine replacement therapy for smoking cessation (Cochrane methodology review). Chichester, UK, John Wiley & Sons, Ltd., 2003
5. Solberg LI, Boyle RG, Davidson G, Magnan S, Link Carlson C, Alesci NL: Aids to quitting tobacco use: How important are they outside controlled trials? Preventive Medicine 2001, 33:53-58
6. Pierce JP, Gilpin EA: Impact of over-the-counter sales on effectiveness of pharmaceutical aids for smoking cessation. JAMA 2002, 288:1260-1264
7. Cromwell J, Bartosch WJ, Fiore MC, Hasselblad V, Baker TB: Cost-effectiveness of the clinical practice recommendations in the AHCPR guideline for smoking cessation. JAMA 1997, 278:1759-1766
8. Song F, Raftery J, Aveyard P, Hyde C, Barton P, Woolacott N: Cost-effectiveness of pharmacological interventions for smoking cessation: A literature review and a decision analytic analysis. Med Decis Making 2002, 22:S26-37
9. LeLorier J, Gregoire G, Benhaddad A, Lapierre J, Derderian F: Discrepancies between meta-analyses and subsequent large randomized, controlled trials. N Engl J Med 1997, 337:536-542
10. Hughes JR, Goldstein MG, Hurt RD, Shiffman S: Recent advances in the pharmacotherapy of smoking. JAMA 1999, 281:72-76
11. Jorenby DE, Leischow SJ, Nides MA, Rennard SI, Johnston JA, Hughes AR, Smith SS, Muramoto ML, Daughton DM, Doan K, Fiore MC, Baker TB: A controlled trial of sustained-release bupropion, a nicotine patch, or both for smoking cessation. New England Journal of Medicine 1999, 340:685-691
12. Croghan GA, Sloan JA, Croghan IT, Novotny P, Hurt RD, DeKrey WL, Mailliard JA, Ebbert LP, Swan DK, Walsh DJ, Wiesenfeld M, Levitt R, Stella P, Johnson PA, Tschetter LK, Loprinzi C: Comparison of nicotine patch alone versus nicotine nasal spray alone versus a combination for treating smokers: a minimal intervention, randomized multicenter trial in a nonspecialized setting. Nicotine and Tobacco Research 2003, 5:181-187
13. Smith SS, Jorenby DE, Leischow SJ, Nides MA, Rennard SI, Johnston JA, Jamerson B, Fiore MC, Baker TB: Targeting smokers at increased risk for relapse: Treating women and those with a history of depression. Nicotine & Tobacco Research 2003, 5:99-109
14. Wetter DW, Kenford SL, Smith SS, Fiore MC, Jorenby DE, Baker TB: Gender differences in smoking cessation. Journal of Consulting and Clinical Psychology 1999, 67:555-562
15. Borkovec TD, Castonguay LG: What is the scientific meaning of empirically supported therapy? Journal of Consulting and Clinical Psychology 1998, 66:136-142
16. McFall RM: Smoking cessation research. Journal of Consulting & Clinical Psychology 1978, 46:703-712
17. McFall RM: Manifesto for a science of clinical psychology. Clinical Psychologist 1991, 44:75-88
18. West R, Hajek P, Nilsson F, Foulds J, May S, Meadows A: Individual differences in preferences for and responses to four nicotine replacement products. Psychopharmacology (Berl) 2001, 153:225-230
19. Gold PB, Rubey RN, Harvey RT: Naturalistic, self-assignment comparative trial of bupropion SR, a nicotine patch, or both for smoking cessation treatment in primary care. Am J Addict 2002, 11:315-331
20. Gonzales D, Bjornson W, Durcan MJ, White JD, Johnston JA, Buist S, Sachs DPL, Rigotti NA, Niaura R, Taylor Hays J, Hurt RD: Effects of gender on relapse prevention in smokers treated with bupropion SR. American Journal of Preventive Medicine 2002, 22:234-239

21. Smith B, Sechrest L: Treatment of Aptitude X Treatment interactions. Journal of Consulting and Clinical Psychology 1991, 59:233-244
22. Centers for Disease Control and Prevention: Use of FDA-approved pharmacological treatments for tobacco dependence - United States, 1984-1998. MMWR 2000, 49:665-668
23. Pierce JP, Gilpin E, Farkas AJ: Nicotine patch use in the general population: results from the 1993 California Tobacco Survey. Journal of the Nationall Cancer Institute 1995, 87:87-93
24. Irvin JE, Hendricks PS, Brandon TH: The increasing recalcitrance of smokers in clinical trials II: Pharmacotherapy trials. Nicotine Tob Res 2003, 5:27-35
25. Shiffman S, Dresler CM, Hajek P, Gilburt SJA, Targett DA, Strahs KR: Efficacy of a nicotine lozenge for smoking cessation. Archives of Internal Medicine 2002, 162:1267-1276
26. Dale LC, Hurt RD, Offord KP, Lawson GM, Croghan IT, Schroeder DR: High-dose nicotine patch therapy. Percentage of replacement and smoking cessation. Jama 1995, 274:1353-1358
27. Russell MA: Nicotine replacement: The role of blood nicotine levels, their rate of change, and nicotine tolerance. Nicotine replacement: A critical evaluation. Edited by Pomerleau OF, Pomerleau CS. New York, Alan R. Liss, Inc., 1988, pp 63-94
28. Choi DC: Use of 2- and 4-mg lozenges delivers some 25 to 27% more nicotine during a quit attempt than do the 2- and 4-mg nicotine gum, respectively. (in press)
29. Hughes JR, Stead LF, Lancaster T: Antidepressants for smoking cessation. Cochrane Database Syst Rev 2003:CD000031
30. Hall SM, Humfleet GL, Reus VI, Munoz RF, Hartz DT, Maude-Griffin R: Psychological intervention and antidepressant treatment in smoking cessation. Archives of General Psychiatry 2002, 59:930-936
31. Shiffman S, Gitchell J, Pinney JM, Burton SL, Kemper KE, Lara EA: Public health benefit of over-the-counter nicotine medications. Tobacco Control 1997, 6:306-310
32. Zhu S, Melcer T, Sun J, Rosbrook B, Pierce JP: Smoking cessation with and without assistance: A population-based analysis. American Journal of Preventive Medicine 2000, 18:305-311
33. Lerman C, Roth D, Kaufmann V, Audrain J, Hawk L, Liu A, Niaura R, Epstein L: Mediating mechanisms for the impact of bupropion in smoking cessation treatment. Drug Alcohol Depend 2002, 67:219-223
34. Bohadana A, Nilsson F, Rasmussen T, Martinet Y: Nicotine inhaler and nicotine patch as a combination therapy for smoking cessation: a randomized, double-blind, placebo-controlled trial. Arch Intern Med 2000, 160:3128-3134
35. Piper ME, Federman EB, Smith SS, Fiore MC, Baker TB: Efficacy of bupropion SR alone and combined with 4-mg nicotine gum. Poster to be presented at the 10th Annual Meeting of the Society for Research on Nicotine and Tobacco, Scottsdale, AZ, 2004
36. Blondal T, Gudmundsson LJ, Tomasson K, Jonsdottir D, Hilmarsdottir H, Kristjansson F, Nilsson F, Bjornsdottir US: The effects of fluoxetine combined with nicotine inhalers in smoking cessation--a randomized trial. Addiction 1999, 94:1007-1015
37. Hughes JR: An algorithm for smoking cessation. Archives of Family Medicine 1994, 3:280-285
38. Hughes JR: Four beliefs that may impede progress in the treatment of smoking. Tobacco Control 1999, 8:323-326
39. Fiore MC, McCarthy DE, Jackson TC, Zehner ME, Jorenby DE, Mielke MM, Smith S, Guiliani TA, Baker TB: Integrating smoking cessation treatment into primary care: An effectiveness study. Preventive Medicine (in press)
40. Lerman C, Shields PG, Wileyto EP, Audrain J, Pinto A, Hawk L, Krishnan S, Niaura R, Epstein L: Pharmacogenetic investigation of smoking cessation treatment. Pharmacogenetics 2002, 12:627-634
41. Piper ME, Fox BJ, Welsch SK, Fiore MC, Baker TB: Gender and racial/ethnic differences in tobacco-dependence treatment: A commentary and research recommendations. Nicotine & Tobacco Research 2001, 3:291-297
42. State-specific prevalence of current cigarette smoking among adults- United States, 2002. MMWR 2004, 52:1277-1280
43. Eissenberg T, Adams C, Riggins EC, 3rd, Likness M: Smokers' sex and the effects of tobacco cigarettes: subject-rated and physiological measures. Nicotine and Tobacco Research 1999, 1:317-324
44. Perkins KA, Donny E, Caggiula AR: Sex differences in nicotine effects and self-administration: review of human and animal evidence. Nicotine Tob Res 1999, 1:301-315

45. Fagerstrom KO: Measuring degree of physical dependence to tobacco smoking with reference to individualization of treatment. Addict Behav 1978, 3:235-241
46. Russell MA, Peto J, Patel UA: The classification of smoking by factorial structure of motives. Journal of the Royal Statistical Society 1974, 137:313-333
47. Tonnesen P, Fryd V, Hansen M, Helsted J, Gunnersen AB, Forchammer H, Stockner M: Effect of nicotine chewing gum in combination with group counseling on the cessation of smoking. New England Journal of Medicine 1988, 318:15-18
48. Garvey AJ, Kinnunen T, Nordstrom BL, Utman CH, Doherty K, Rosner B, Vokonas PS: Effects of nicotine gum dose by level of nicotine dependence. Nicotine Tob Res 2000, 2:53-63
49. Heatherton TF, Kozlowski LT, Frecker RC, Fagerstrom KO: The Fagerstrom Test for Nicotine Dependence: a revision of the Fagerstrom Tolerance Questionnaire. Br J Addict 1991, 86:1119-1127
50. Piper ME, Ma GX, Bolt DM, McCarthy DE, Baker TB: The Wisconsin Predicting Relapse in Smoking Measure (WI-PRISM) - A new measure of relapse proneness. Poster presented at the 10th Annual Meeting for the Society for Research on Nicotine and Tobacco, Scottsdale, AZ 2004
51. Piper ME, Piasecki TM, Federman EB, Bolt DM, Smith SS, Fiore MC, Baker TB: A multiple motives approach to tobacco dependence: The Wisconsin Inventory of Smoking Dependence Motives (WISDM-68). Journal of Consulting & Clinical Psychology (in press)
52. Hall SM, Munoz RF, Reus VI, Sees KL: Nicotine, negative affect, and depression. J Consult Clin Psychol 1993, 61:761-767
53. Kinnunen T, Doherty K, Militello FS, Garvey AJ: Depression and smoking cessation: characteristics of depressed smokers and effects of nicotine replacement. J Consult Clin Psychol 1996, 64:791-798
54. Brandon TH: Negative affect as motivation to smoke. Current Directions in Psychological Science 1994, 3:33-37
55. Hitsman B, Borrelli B, McChargue DE, Spring B, Niaura R: History of depression and smoking cessation outcome: a meta-analysis. J Consult Clin Psychol 2003, 71:657-663
56. Hall SM, Munoz RF, Reus VI: Cognitive-behavioral intervention increases abstinence rates for depressive-history smokers. J Consult Clin Psychol 1994, 62:141-146
57. Zelman DC, Brandon TH, Jorenby DE, Baker TB: Measures of affect and nicotine dependence predict differential response to smoking cessation treatments. Journal of Consulting and Clinical Psychology 1992, 60:943-952
58. Kahler CW, Brown RA, Ramsey SE, Niaura R, Abrams DB, Goldstein MG, Mueller TI, Miller IW: Negative mood, depressive symptoms, and major depression after smoking cessation treatment in smokers with a history of major depressive disorder. Journal of Abnormal Psychology 2002, 111:670-675
59. Alloy L, Abramson L, Metalsky G, Harlages S: The Depression Proneness Inventory: A stress-responsive measure of vulnerability to depression. (unpublished manuscript) 1990
60. Patrick CJ, Curtin JJ, Tellegen A: Development and validation of a brief form of the Multidimensional Personality Questionnaire. Psychol Assess 2002, 14:150-163
61. Watson D, Clark LA, Tellegen A: Development and validation of brief measures of positive and negative affect: the PANAS scales. J Pers Soc Psychol 1988, 54:1063-1070
62. Hughes JR, Novy P, Hatsukami DK, Jensen J, Callas PW: Efficacy of nicotine patch in smokers with a history of alcoholism. Alcohol Clin Exp Res 2003, 27:946-954
63. Piasecki TM, Jorenby DE, Smith SS, Fiore MC, Baker TB: Smoking withdrawal dynamics: I. Abstinence distress in lapsers and abstainers. Journal of Abnormal Psychology 2003, 112:3-13
64. Piasecki TM, Jorenby DE, Smith SS, Fiore MC, Baker TB: Smoking withdrawal dynamics: II. Improved tests of withdrawal-relapse relations. Journal of Abnormal Psychology 2003, 112:14-27
65. Shiffman S, Johnston JA, Khayrallah M, Elash CA, Gwaltney CJ, Paty JA, Gnys M, Evoniuk G, DeVeaugh-Geiss J: The effect of bupropion on nicotine craving and withdrawal. Psychopharmacology (Berl) 2000, 148:33-40
66. Baker TB, Piper ME, McCarthy DE, Majeskie MR, Fiore MC: Addiction motivation reformulated: An affective processing modle of negative reinforcement. Psychol Rev (in press)
67. Patten CA, Martin JE: Does nicotine withdrawal affect smoking cessation? Clinical and theoretical issues. Annals of Behavioral Medicine 1996, 18:190-200
68. Robinson TE, Berridge KC: The neural basis of drug craving: an incentive-sensitization theory of addiction. Brain Res Brain Res Rev 1993, 18:247-291

69. van Ree JM, Gerrits MA, Vanderschuren LJ: Opioids, reward and addiction: An encounter of biology, psychology, and medicine. Pharmacol Rev 1999, 51:341-396
70. Cole DA, Maxwell SE: Testing mediational models with longitudinal data: Questions and tips in the use of structural equations modeling. Journal of Abnormal Psychology (in press)
71. Kazdin AE: Overview of research design issues in clinical psychology. Handbook of research methods in clinical psychology. Edited by Kendall PC, Butcher JN, Holmbeck GN. New York, Wiley, 1999, pp 3-30
72. Shiffman S, Engberg JB, Paty JA, Perz WG, Gnys M, Kassel JD, Hickcox M: A day at a time: predicting smoking lapse from daily urge. J Abnorm Psychol 1997, 106:104-116
73. American Psychiatric Association: Diagnostic and Statistical Manual of Mental Disorders, Fourth Edition. Washington, D.C., American Psychiatric Association, 1994
74. Goldberg SR: Stimuli associated with drug injections as events that control behavior. Pharmacol Rev 1976, 27:325-340
75. Harrison AA, Liem YT, Markou A: Fluoxetine combined with a serotonin-1A receptor antagonist reversed reward deficits observed during nicotine and amphetamine withdrawal in rats. Neuropsychopharmacology 2001, 25:55-71
76. Schuster CR, Thompson T: Self administration of and behavioral dependence on drugs. Annu Rev Pharmacol 1969, 9:483-502
77. Shiffman S, Waters AJ: Negative affect and smoking lapses: A prospective analysis. Journal of Abnormal Psychology (in press)
78. Wileyto EP, Niaura R, Audrain J, Kaufmann V, Epstein L, Brown RA, Lerman C: Time to event analysis of bupropion clinical trial data (poster presentation). Presented at the Society for Research in Nicotine and Tobacco, New Orleans, LA, 2003
79. Shiffman S, Shadel WG, Niaura R, Khayrallah MA, Jorenby DE, Ryan CF, Ferguson CL: Efficacy of acute administration of nicotine gum in relief of cue-provoked cigarette craving. Psychopharmacology (Berl) 2003, 166:343-350
80. Rossing MA: Genetic influences on smoking: candidate genes. Environ Health Perspect 1998, 106:231-238
81. Steinlein OK, Deckert J, Nothen MM, Franke P, Maier W, Beckmann H, Propping P: Neuronal nicotinic acetylcholine receptor alpha 4 subunit (CHRNA4) and panic disorder: an association study. Am J Med Genet 1997, 74:199-201
82. Picciotto MR, Zoli M, Rimondini R, Lena C, Marubio LM, Pich EM, Fuxe K, Changeux JP: Acetylcholine receptors containing the beta2 subunit are involved in the reinforcing properties of nicotine. Nature 1998, 391:173-177
83. Steinlein OK, Mulley, J. C., Propping, P., Wallace, R. H., Phillips, H. A., Sutherland, G.R., Scheffer, I.E., Berkovic, S. F.: A missense mutation in the neuronal nicotinic acetylcholine receptor alpha 4 subunit is associated with autosomal dominant nocturnal frontal lobe epilepsy. National Genetics 1995, 11:201-203
84. Silverman MA, Neale MC, Sullivan PF, Harris-Kerr C, Wormley B, Sadek H, Ma Y, Kendler KS, Straub RE: Haplotypes of four novel single nucleotide polymorphisms in the nicotinic acetylcholine receptor beta2-subunit (CHRNB2) gene show no association with smoking initiation or nicotine dependence. Am J Med Genet 2000, 96:646-653
85. Lerman C, Caporaso NE, Audrain J, Main D, Bowman ED, Lockshin B, Boyd NR, Shields PG: Evidence suggesting the role of specific genetic factors in cigarette smoking. Health Psychol 1999, 18:14-20
86. Comings DE, Gade R, Wu S, Chiu C, Dietz G, Muhleman D, Saucier G, Ferry L, Rosenthal RJ, Lesieur HR, Rugle LJ, MacMurray P: Studies of the potential role of the dopamine D1 receptor gene in addictive behaviors. Mol Psychiatry 1997, 2:44-56
87. Sabol SZ, Nelson ML, Fisher C, Gunzerath L, Brody CL, Hu S, Sirota LA, Marcus SE, Greenberg BD, Lucas FRt, Benjamin J, Murphy DL, Hamer DH: A genetic association for cigarette smoking behavior. Health Psychol 1999, 18:7-13
88. Comings DE, Ferry L, Bradshaw-Robinson S, Burchette R, Chiu C, Muhleman D: The dopamine D2 receptor (DRD2) gene: a genetic risk factor in smoking. Pharmacogenetics 1996, 6:73-79
89. Noble EP, St Jeor ST, Ritchie T, Syndulko K, St Jeor SC, Fitch RJ, Brunner RL, Sparkes RS: D2 dopamine receptor gene and cigarette smoking: a reward gene? Med Hypotheses 1994, 42:257-260

90. Spitz MR, Shi H, Yang F, Hudmon KS, Jiang H, Chamberlain RM, Amos CI, Wan Y, Cinciripini P, Hong WK, Wu X: Case-control study of the D2 dopamine receptor gene and smoking status in lung cancer patients. J Natl Cancer Inst 1998, 90:358-363
91. Bergen AW, Korczak JF, Weissbecker KA, Goldstein AM: A genome-wide search for loci contributing to smoking and alcoholism. Genet Epidemiol 1999, 17 Suppl 1:S55-60
92. Duggirala R, Almasy L, Blangero J: Smoking behavior is under the influence of a major quantitative trait locus on human chromosome 5q. Genet Epidemiol 1999, 17 Suppl 1:S139-144
93. Lerman C, Berrettini W: Elucidating the role of genetic factors in smoking behavior and nicotine dependence. Am J Med Genet 2003, 118B:48-54
94. Caporaso NG, A.: Issues involving biomarkers in the study of the genetics of human cancer. IARC Sci Publ 1997, 142:237-250
95. Sullivan P, Jiang, Y, Neale, MC, Kendler, KS, Straub, RE . Association of the tryptophan hydroxylase gene with smoking initiation but not progression to nicotine dependence. Am J Med Genet 2001, 105:479-484
96. Pianezza ML, Sellers EM, Tyndale RF: Nicotine metabolism defect reduces smoking. Nature 1998, 393:750
97. Oscarson M, Gullsten H, Rautio A, Bernal ML, Sinues B, Dahl ML, Stengard JH, Pelkonen O, Raunio H, Ingelman-Sundberg M: Genotyping of human cytochrome P450 2A6 (CYP2A6), a nicotine C-oxidase. FEBS Lett 1998, 438:201-205
98. Zabetian CP, Gelernter J, Cubells JF: Functional variants at CYP2A6: new genotyping methods, population genetics, and relevance to studies of tobacco dependence. Am J Med Genet 2000, 96:638-645
99. London SJ, Idle JR, Daly AK, Coetzee GA: Genetic variation of CYP2A6, smoking, and risk of cancer. Lancet 1999, 353:898-899
100. Garcia-Closas M, Caporaso N, Kelsey KK, Christain DC: Association between CYP1A1 polymorphism and smoking in a control population: Implications for the study of genetic effects on cancer risk (abstract). Proceedings of the American Association of Cancer Research 1997, 38:211
101. McCarthy DE, Fiore MC, Piasecki TM, Baker TB: Life before and after quitting smoking. Journal of Abnormal Psychology (under review)
102. McCarthy DE, Piasecki, T. M., Lawrence, D. L., Fiore, M. C., & Baker, T. B.: Efficacy of bupropion SR and individual counseling among adults attempting to quit smoking. Poster to be presented at the 10th Annual Meeting of the Society for Research in Nicotine and Tobacco 2004
103. Meyer G, McPhail G, Fiore MC: Using smoker demographics to select cost-effective recruitment advertising. Poster presented at the 2003 National Conference on Tobacco or Health 2003
104. Welsch SK, Smith SS, Wetter DW, Jorenby DE, Fiore MC, Baker TB: Development and validation of the Wisconsin Smoking Withdrawal Scale. Experimental and Clinical Psychopharmacology 1999, 7:354-361
105. Robins LN, Regier DA: Psychiatric disorders in America. New York, MacMillan, 1991
106. Kessler RC, McGonagle KA, Zhao S, Nelson CB, Hughes M, Eshleman S, Wittchen HU, Kendler KS: Lifetime and 12-month prevalence of DSM-III-R psychiatric disorders in the United States. Results from the National Comorbidity Survey. Archives of General Psychiatry 1994, 51:8-19
107. Wittchen HU, Robins LN, Cottler LB, Sartorius N, Burke JD, Regier D: Cross-cultural feasibility, reliability and sources of variance of the Composite International Diagnostic Interview (CIDI). The Multicentre WHO/ADAMHA Field Trials. Br J Psychiatry 1991, 159:645-653, 658
108. Wittchen HU: Reliability and validity studies of the WHO--Composite International Diagnostic Interview (CIDI): a critical review. J Psychiatr Res 1994, 28:57-84
109. Holden C: Mental health. Global survey examines impact of depression. Science 2000, 288:39-40
110. Girolamo G, Bassi M: Community surveys of mental disorders: Recent achievements and works in progress. Current Opinion in Psychiatry 2003, 16:403-411
111. Kessler RC, Berglund P, Demler O, Jin R, Koretz D, Merikangas KR, Rush AJ, Walters EE, Wang PS: The epidemiology of major depressive disorder: results from the National Comorbidity Survey Replication (NCS-R). Jama 2003, 289:3095-3105
112. Smith SS, Jorenby DE, Fiore MC, Anderson JE, Mielke MM, Beach KE, Piasecki TM, Baker TB: Strike while the iron is hot: Can stepped-care treatments resurrect relapsing smokers? Journal of Consulting & Clinical Psychology 2001, 69:429-439

113. Roberts LJ, McCrady BS: Alcohol problems in intimate relationships: Identification and intervention (NIH publication number 03-5284). Washington, D.C., U.S. Government Printing Office, 2003
114. Dawson DA: Methodological issues in measuring alcohol use., National Institute on Alcohol Abuse and Alcoholism Publication (on-line) Available at: http://www.niaaa.nih.gov/publications/arh27-1/18-29.htm, 2003
115. Sobell LC, Sobell MB, Leo GI, Cancilla A: Reliability of a timeline method: Assessing normal drinkers' reports of recent drinking and a comparative evaluation across several populations. Br J Addict 1988, 83:393-402
116. Greenfield TK, Kerr WC: Tracking alcohol consumption over time, National Institute on Alcohol Abuse and Alcoholism. Available at: http://www.niaaa.nih.gov/publications/arh27-1/30-38.htm,, 2003
117. Miller WR, Tonigan JS, Longabaugh R: The Drinker Inventory of Consequences (DrInC): An instrument for assessing adverse consequences of alcohol abule. Washington, D.C., U.S. Government Printing Office, 1995
118. Willett WC, Colditz GA: Approaches for conducting large cohort studies. Epidemiol Rev 1998, 20:91-99
119. Rimm EB, Giovannucci EL, Stampfer MJ, Colditz GA, Litin LB, Willett WC: Reproducibility and validity of an expanded self-administered semiquantitative food frequency questionnaire among male health professionals. Am J Epidemiol 1992, 135:1114-1126; discussion 1127-1136
120. Giovannucci E, Stampfer MJ, Colditz GA, Rimm EB, Trichopoulos D, Rosner BA, Speizer FE, Willett WC: Folate, methionine, and alcohol intake and risk of colorectal adenoma. J Natl Cancer Inst 1993, 85:875-884
121. Willett W, Stampfer M, Chu NF, Spiegelman D, Holmes M, Rimm E: Assessment of questionnaire validity for measuring total fat intake using plasma lipid levels as criteria. Am J Epidemiol 2001, 154:1107-1112
122. London SJ, Sacks FM, Caesar J, Stampfer MJ, Siguel E, Willett WC: Fatty acid composition of subcutaneous adipose tissue and diet in postmenopausal US women. Am J Clin Nutr 1991, 54:340-345
123. Hunter DJ, Rimm EB, Sacks FM, Stampfer MJ, Colditz GA, Litin LB, Willett WC: Comparison of measures of fatty acid intake by subcutaneous fat aspirate, food frequency questionnaire, and diet records in a free-living population of US men. Am J Epidemiol 1992, 135:418-427
124. Ascherio A, Rimm EB, Giovannucci EL, Colditz GA, Rosner B, Willett WC, Sacks F, Stampfer MJ: A prospective study of nutritional factors and hypertension among US men. Circulation 1992, 86:1475-1484
125. Stryker WS, Kaplan LA, Stein EA, Stampfer MJ, Sober A, Willett WC: The relation of diet, cigarette smoking, and alcohol consumption to plasma beta-carotene and alpha-tocopherol levels. Am J Epidemiol 1988, 127:283-296
126. Aldoori WH, Giovannucci EL, Rimm EB, Wing AL, Trichopoulos DV, Willett WC: A prospective study of diet and the risk of symptomatic diverticular disease in men. Am J Clin Nutr 1994, 60:757-764
127. Ascherio A, Hennekens CH, Buring JE, Master C, Stampfer MJ, Willett WC: Trans-fatty acids intake and risk of myocardial infarction. Circulation 1994, 89:94-101
128. Ascherio A, Rimm EB, Giovannucci EL, Spiegelman D, Stampfer M, Willett WC: Dietary fat and risk of coronary heart disease in men: cohort follow up study in the United States. Bmj 1996, 313:84-90
129. Curhan GC, Willett WC, Rimm EB, Spiegelman D, Stampfer MJ: Prospective study of beverage use and the risk of kidney stones. Am J Epidemiol 1996, 143:240-247
130. Curhan GC, Willett WC, Rimm EB, Stampfer MJ: A prospective study of dietary calcium and other nutrients and the risk of symptomatic kidney stones. N Engl J Med 1993, 328:833-838
131. Curhan GC, Willett WC, Speizer FE, Spiegelman D, Stampfer MJ: Comparison of dietary calcium with supplemental calcium and other nutrients as factors affecting the risk for kidney stones in women. Ann Intern Med 1997, 126:497-504
132. Giovannucci E, Rimm EB, Stampfer MJ, Colditz GA, Ascherio A, Willett WC: Intake of fat, meat, and fiber in relation to risk of colon cancer in men. Cancer Res 1994, 54:2390-2397
133. Giovannucci E, Rimm EB, Ascherio A, Stampfer MJ, Colditz GA, Willett WC: Alcohol, low-methionine--low-folate diets, and risk of colon cancer in men. J Natl Cancer Inst 1995, 87:265-273
134. Hankinson SE, Stampfer MJ, Seddon JM, Colditz GA, Rosner B, Speizer FE, Willett WC: Nutrient intake and cataract extraction in women: a prospective study. Bmj 1992, 305:335-339
135. Manson JE, Gaziano JM, Spelsberg A, Ridker PM, Cook NR, Buring JE, Willett WC, Hennekens CH: A secondary prevention trial of antioxidant vitamins and cardiovascular disease in women. Rationale, design, and methods. The WACS Research Group. Ann Epidemiol 1995, 5:261-269

136. Rimm EB, Ascherio A, Giovannucci E, Spiegelman D, Stampfer MJ, Willett WC: Vegetable, fruit, and cereal fiber intake and risk of coronary heart disease among men. Jama 1996, 275:447-451
137. Salmeron J, Ascherio A, Rimm EB, Colditz GA, Spiegelman D, Jenkins DJ, Stampfer MJ, Wing AL, Willett WC: Dietary fiber, glycemic load, and risk of NIDDM in men. Diabetes Care 1997, 20:545-550
138. Salmeron J, Manson JE, Stampfer MJ, Colditz GA, Wing AL, Willett WC: Dietary fiber, glycemic load, and risk of non-insulin-dependent diabetes mellitus in women. Jama 1997, 277:472-477
139. Willett WC, Stampfer MJ, Colditz GA, Rosner BA, Speizer FE: Relation of meat, fat, and fiber intake to the risk of colon cancer in a prospective study among women. N Engl J Med 1990, 323:1664-1672
140. Willett WC, Stampfer MJ, Manson JE, Colditz GA, Speizer FE, Rosner BA, Sampson LA, Hennekens CH: Intake of trans fatty acids and risk of coronary heart disease among women. Lancet 1993, 341:581-585
141. Wolk A, Manson JE, Stampfer MJ, Colditz GA, Hu FB, Speizer FE, Hennekens CH, Willett WC: Long-term intake of dietary fiber and decreased risk of coronary heart disease among women. Jama 1999, 281:1998-2004
142. Rifas-Shiman SL, Willett WC, Lobb R, Kotch J, Dart C, Gillman MW: PrimeScreen, a brief dietary screening tool: reproducibility and comparability with both a longer food frequency questionnaire and biomarkers. Public Health Nutr 2001, 4:249-254
143. Craig CL, Marshall AL, Sjostrom M, Bauman AE, Booth ML, Ainsworth BE, Pratt M, Ekelund U, Yngve A, Sallis JF, Oja P: International physical activity questionnaire: 12-country reliability and validity. Med Sci Sports Exerc 2003, 35:1381-1395
144. Schneider PL, Crouter SE, Lukajic O, Bassett DR, Jr.: Accuracy and reliability of 10 pedometers for measuring steps over a 400-m walk. Med Sci Sports Exerc 2003, 35:1779-1784
145. Frisch MB, Cornell J, Villanueva M, Retzlaff PJ: Clinical validation of the Quality of Life Inventory: A measure of life satisfaction for use in treatment planning and outcome assessments. Psychological Assessment 1992, 4:92-101
146. Holmes TH, Rahe RH: The Social Readjustment Rating Scale. J Psychosom Res 1967, 11:213-218
147. Mandell W: SAFE Social Network Questionnaire. Baltimore, MD, The Johns Hopkins University, School of Hygiene and Public Health, 1991
148. Leonard KE, Mudar P: Peer and partner drinking and the transition to marriage: a longitudinal examination of selection and influence processes. Psychol Addict Behav 2003, 17:115-125
149. McCallister L, Fischer C: A procedure for surveying personal networks. Sociological Methods & Research 1978, 7:131-148
150. Brewer DD: Forgetting in the recall-based elicitation of personal and social networks. Social Networks 2000, 22:29-43
151. Schumm WR, Paff-Bergen LA, Hatch RC, Obiorah FC, Copeland JM, Meens LD, Bugaighis MA: Concurrent and discriminant validity of the Kansas N=Marital Satisfaction Scale. Journal of Marriage and the Family 1986, 48:381-387
152. Kenford SL, Fiore MC, Jorenby DE, Smith SS, Wetter D, Baker TB: Predicting smoking cessation. Who will quit with and without the nicotine patch. JAMA 1994, 271:589-594
153. Westman EC, Behm FM, Simel DL, Rose JE: Smoking behavior on the first day of a quit attempt predicts long-term abstinence. Archives of Internal Medicine 1997, 157:33-340
154. Fiore MC, Kenford SL, Jorenby DE, Wetter DW, Smith SS, Baker TB: Two studies of the clinical effectiveness of the nicotine patch with different counseling treatments. Chest 1994, 105:524-533
155. Hughes JR, Keely JP, Niaura RS, Ossip-Klein DJ, Richmond RL, Swan GE: Measures of abstinence in clinical trials: Issues and recommendations. Nicotine & Tobacco Research 2003, 5:13-25
156. Holm S: A simple sequentially rejective Bonferroni Test procedure. Scandinavian Journal of Statistics 1979, 6:65-70
157. Westfall PH, Tobias RD, Rom D, Wolfinger RD, Hochberg Y: Multiple comparisons and multiple tests using the SAS system. Cary, NC, SAS Institute, Inc., 1999
158. Liang KY, Zeger SL: Longitudinal data analysis using generalized linear models. Biometrika 1986, 73:13-22
159. Shiffman S, Elash CA, Paton SM, Gwaltney CJ, Paty JA, Clark DB, Liu KS, Di Marino ME: Comparative efficacy of 24-hour and 16-hour transdermal nicotine patches for relief of morning craving. Addiction 2000, 95:1185-1195

160. Hall SM, Delucchi KL, Velicer WF, Kahler CW, Ranger-Moore J, Hedeker D, Tsoh JY, Niaura R: Statistical analysis of randomized trials in tobacco treatment: longitudinal designs with dichotomous outcome. Nicotine Tob Res 2001, 3:193-202
161. Heath AC, Madden, P.A., Martin, N.G.: Statistical methods in genetic research on smoking. Stat Methods Med Res 1998, 7:165-186
162. Piasecki TM, Jorenby DE, Smith SS, Fiore MC, Baker TB: Smoking Withdrawal Dynamics: III. Correlates of Withdrawal Heterogeneity. Exp Clin Psychopharmacol 2003, 11:276-285
163. Piasecki TM, Niaura R, Shadel WG, Abrams D, Goldstein M, Fiore MC, Baker TB: Smoking withdrawal dynamics in unaided quitters. Journal of Abnormal Psychology 2000, 109:74-86
164. Abu-Hanna A, de Keizer N: Integrating classification trees with local logistic regression in Intensive Care prognosis. Artif Intell Med 2003, 29:5-23
165. Tsien CL, Fraser HS, Long WJ, Kennedy RL: Using classification tree and logistic regression methods to diagnose myocardial infarction. Medinfo 1998, 9 Pt 1:493-497
166. Chan KY, Loh WY: An algorithm for building accurate and comprehensible logistic regression trees. Journal of Computational and Graphical Statistics (in press)
167. Breiman L, Friedman JH, Olshen RA, Stone CJ: Classification and regression trees. Belmont, CA, Wadsworth International Group, 1984
168. Crichton NJ, Hinde JP, Marchini J: Models for diagnosing chest pain: Is CART helpful? Statistics in Medicine 1997, 16:717-727
169. Mair J, Smidt J, Lechleitner P, Dienstl F, Puschendorf B: A decision tree for the early diagnosis of acute myocardial infarction in nontraumatic chest pain patients at hospital admission. Chest 1995, 108:1502-1509
170. Loh WY, Shih YS: Split selection methods for classification trees. Statistica Sinica 1997, 7:815-840
171. Dawes RM: The robust beauty of improper linear models in decision making. American Psycholgist 1979, 34:571-582
172. Gigerenzer G, Todd PM, ABC Research Group: Simple heuristics that make us smart. Oxford, UK, Oxford University Press, 1999
173. Hosmer DW, Lemeshow S: Applied Logistic Regression, Second Edition. New York, John Wiley & Sons, Inc., 2000
174. Gong G: Cross-validation, the jackknife, and the bootstrap: Excess error estimation in forward logistic regression. Journal of the American Statistical Association 1986, 81:108-113
175. Swets JA, Dawes RM, Monahan J: Better decisions through science. Sci Am 2000, 283:82-87
176. Nunnally JC, Bernstein IH: Psychometric Theory. New York, McGraw Hill, Inc., 1994
177. Kenny DA, Kashy DA, Bolger N: Data analysis in social psychology. The handbook of social psychology. Edited by Gilbert DT, Fiske ST. New York, McGraw-Hill, 1998, pp 233-265
178. MacKinnon DP, Dwyer JH: Estimating mediated effects in prevention studies. Eval Rev 1993, 17:144-158
179. Shrout PE, Bolger N: Mediation in experimental and nonexperimental studies: New procedures and recommendations. Psychological Methods 2002, 7:422-445
180. Sobel ME: Some new results on indirect effects and their standard errors in covariance structure models. Sociological methodology. Edited by Tuma N. Washington, D.C., American Sociological Association, 1986, pp 159-186
181. Collins LM, Graham JW, Flaherty B: An alternative framework for defining mediation. Multivariate Behavioral Research 1998, 33:295-312
182. Krull JL, MacKinnon DP: Multilevel mediation modeling in group-based intervention studies. Eval Rev 1999, 23:418-444
183. Kenny DA, Korchmaros JD, Bolger N: Lower level mediation in multilevel models. Psychol Methods 2003, 8:115-128
184. Bryk AS, Raudenbush SW: Hierarchical linear models, application and data analysis methods. Newbury Park, CA, Sage Publications, 1992
185. Silagy C, Lancaster T, Stead L, Mant D, Fowler G: Nicotine replacement therapy for smoking cessation. Cochrane Database of Systematic Reviews 2002:CD000146
186. Fox BJ, Welsch SK, Fiore MC, Baker TB, Hasselblad V: Have the nicotine patch and nicotine gum lost their efficacy over time? Poster presented at the annual meeting of the Society for Research on Nicotine and Tobacco, Seattle, WA 2001, March

H. Consortium/Contractual Arrangements

N/A

I. Consultants

University of Wisconsin budgetary procedures do not permit co-investigators who are not UW employees to be listed in the personnel section of the budget. Hence, co-investigators who are not UW employees are listed as consultants.

J. Letters of Support/Commitment

See attached letters.

HSC# 2004-0094

Research Subject Information and Consent Form

University of Wisconsin Center for Health Sciences
Medical School - Department of Medicine
Madison WI 53792

Protocol Title: Tobacco Dependence: Treatment and Outcomes.
Subtitled: Pharmacotherapies: Efficacy, Mechanisms and Algorithms (*Efficacy*) AND Natural History of Smoking and Quitting: Longterm Outcomes (*Longterm Outcomes*).

Study Sponsor: **National Institutes of Health (NIH)**

Principal Investigator: Timothy Baker, Ph.D.

Subject Name ___________________________ Subject Initials ___________

Invitation.

You are invited to participate in a clinical research study about smoking cessation, conducted by Dr. Tim Baker, designed to study which medications work best, how best to match individuals to medications, how to combine medications, and how medications achieve their effects. In addition, this study will follow subjects for three years after a quit attempt to track smoking behavior and health changes.

You are being invited to participate because you smoke more than 10 cigarettes per day, are not currently using any smoking cessation product nor participating in a stop-smoking program, are aged 18 or older, and would like to quit smoking.

Your participation will involve taking a medication to help you in your attempt to quit smoking. There are five different active medication conditions (nicotine patch, nicotine

lozenge, bupropion, patch + lozenge, and bupropion + lozenge) or five placebo conditions (placebo patch, placebo lozenge, placebo bupropion, placebo patch +placebo lozenge, and placebo bupropion + placebo lozenge) to which you can be randomly assigned. You have a 13 % chance of being assigned to one of the placebo conditions. The placebo conditions do not contain any active medication. You will attend 10 study visits at our clinic and be contacted by telephone 6 times over the first year. The second and third year of the study will require three study visits at our clinic and 2 telephone contacts.

This document describes the clinical research study and your role in it if you decide to participate. Please read this document carefully and do not hesitate to ask questions at any time. Your participation in this study is voluntary. It is up to you to decide whether or not you want to participate.

If you decide to participate, you will receive a signed copy of this document for your records. Also, if you decide to participate, you can change your mind at any time and withdraw from the study without giving a reason. This will not affect the standard of care you receive. You will be one of 1520 participants (maximum) enrolled in this study conducted at the Center for Tobacco Research and Intervention in either Madison or Milwaukee.

Because this study will last for a total of three years, if you do not plan to or expect to be able to participate in this study for that length of time for any reason (such as, job relocation, military service, planned pregnancy etc.), please let the study personnel know before continuing to read further.

It is very important that you tell study staff about all medical problems you have now or have had in the past, medications you have been, or are currently taking, or any allergic reactions you have had to medications, foods, etc.

What is the purpose of the research study?

This research study will last for three years. The first year involves a comparison of smoking cessation medications, including medications used in combination. The results of this study may allow researchers and clinicians to decide which medications are best for helping to quit smoking as well as what treatments work best for different people. In addition, the first year of the study may help to clarify how these medications work.

The purpose of the second and third years of this research study will be to get long-term results on the physical health, mental health, lifestyle factors, and the overall quality of life of people attempting to quit smoking. The results of this study may help researchers and clinicians better understand the health improvements that come from quitting or not quitting smoking.

Who can participate in this study?

To be in this study you must be medically able to take any of the study medications (bupropion, nicotine patch, and nicotine lozenge). Contraindications for bupropion are uncontrolled hypertension, history or current diagnosis of anorexia or bulimia, history of bipolar illness, recent myocardial infarction, recent heavy alcohol use, seizure history, allergic reactions to 3 or more classes of drugs, use of MAO inhibitors, or allergy to bupropion. Contraindications for the nicotine patch include serious cardiac arrhythmias, angina, recent heart attack, allergy to the nicotine patch, or allergy to adhesive. Contraindications for the nicotine lozenge include allergy to the nicotine lozenge, jaw dysfunction, phenylketonuria, serious cardiac arrhythmias, recent heart attack, pregnancy or hyperthyroidism.

If you are pregnant or breastfeeding you may not participate in this study. If you are a woman, you will be required to use an acceptable method of birth control to prevent pregnancy during the study (IUD, oral contraceptive, barrier methods, or abstinence). You will also be asked to sign a voluntary statement of intent to avoid pregnancy for the duration of the treatment phase of the study. During the study, if you suspect that you are pregnant, you must immediately notify the study physician. If you are pregnant, the study medication will be stopped and you will be referred for follow-up care.

What will my participation involve?

Initial year of this study: At the initial **orientation session** you will be asked questions about your smoking history, your personality, and your activities. You will also be asked questions about your beliefs, experiences, observations and opinions that may affect your attempt to stop cigarette smoking. You will also have your vital signs taken, which will include measurements of your height, weight, pulse, blood pressure, and carbon monoxide (CO) levels. Carbon monoxide is a gas that is found in the lungs at higher levels among cigarettes smokers and is measured by breathing into a small tube. You will be asked to read and complete several paper questionnaires. This visit should take about two hours.

At **visit 1**, you will again complete questionnaires and interviews about your smoking, your medical history, how you are feeling, any stress you have, your mental health, social support (family and friends), what you eat, and alcohol use. You will also receive your first stop smoking counseling session. This visit should take about three hours.

At **visit 2**, about one week later, you will be asked to come to your visit without eating or drinking anything but water for 12 hours and not smoking for 12 hours. You will be asked to give a blood sample (about 5 tablespoons) to test for heart disease (lipoprofiles, creatinine, C Reactive Protein), cotinine, diabetes risk (simultaneous fasting blood glucose and insulin, hemoglobin A1C) and diet (lipoprofile).

The health of your heart will be tested using tests such as ultrasound Carotid Intima-Medial Thickness (Carotid-IMT) and ultrasound Brachial Artery Reactivity Testing

(BART) for blood vessel functioning. Ultrasound scans will be performed on the blood vessel in one of your arms. Ultrasound is a painless test that involves bouncing sound beams off of your artery, allowing the artery to be seen on a screen and measurements made. A blood pressure cuff will be put on your lower arm and inflated above your systolic blood pressure (the blood pressure number on top) for approximately 4 minutes and 30 seconds. After release of the cuff, a repeat ultrasound of the artery in your forearm will be obtained. This has not been reported to be painful in several published research studies or by previous research subjects at the University of Wisconsin Hospital. You will then lie on your back with your head turned to the left. Ultrasound images of an artery in your neck will be obtained and recorded. This will be repeated with your head turned to the right.

You will have an exercise test. You will exercise on a treadmill or exercise bike while breathing through a special mouthpiece. Your blood pressure and electrocardiogram (ECG, measurement of the electrical activity of your heart) will be monitored. The ECG will include placing electrodes on your chest, arms, and legs. You will exercise until one of these three things happens: 1) you feel unable to continue, usually because you are too tired; 2) you develop significant shortness of breath; or 3) we stop you. We will stop you if there are concerns about your ECG or blood pressure in response to the exercise.

Finally, you will be asked to complete a test on the computer. For this test you will be asked to look at 2 pictures side by side on a computer screen. Then, the pictures will disappear and arrows will appear on one side of the computer screen. You will be asked to indicate which direction the arrows point. Some of the slides may be disturbing, but you will only be asked to look at them for a maximum of 2 seconds. You also will be asked to complete this test at either Visit 3 or Visit 1.

Visit two should take about three hours.

The remaining visits during the first year, **Visits 3-9,** all take about 30-90 minutes to complete. These visits include the completion of study questionnaires, vital signs and smoking status.

At Visit 3, you will be given an Electronic Diary (palmtop computers) to keep track of your withdrawal symptoms, urges, negative feelings, stressors, temptation events, positive feelings and pleasurable activities as well as your social activities for one week before and two weeks after your quit attempt. You will be trained on how to use the diary during this visit. The palmtop will prompt you four times a day to complete a short (2-3 minute) survey. You also will be asked to carry a pedometer to measure the distance you walk, for one week before your quit attempt to provide a baseline measure of activity and one week after you quit.

Visits 3-6 are weekly, and Visit 7 two weeks later, and Visit 8 is 4 weeks later. If you are not smoking at month 6, post quit attempt, you will be invited to come in for Visit 9. Almost all participants will be invited to Visit 10. Visit 10 is the beginning of the long-

term follow-up and participants will repeat a number of assessments that were done at the baseline visits.

At Visits 1, 3, 4, 5, 6, and 7 you will receive stop smoking counseling that will last between 10 and 20 minutes. Counseling will focus on understanding your past quit attempts and getting you ready to quit this time. Counseling will also help you develop new skills to cope with urges and avoid dangerous situations where you might be tempted to smoke. Counseling sessions will be audiotaped for quality control and to insure consistency of the counseling content. You will not be personally identified in these recordings. Audiotapes will be kept in a locked file and erased after the supervisor has reviewed them.

In addition, at Visit 3 you will be randomly assigned (like the flip of a coin) to receive one of the five active medication conditions: 1) nicotine patch, 2) nicotine lozenge, 3) bupropion tablets, 4) nicotine patch + lozenge, 5) bupropion tablets + lozenge. Or you will be randomly assigned to one of the five placebo conditions (placebo patch, placebo lozenge, placebo bupropion, placebo patch + placebo lozenge, placebo bupropion + placebo lozenge). A placebo contains only non-active medication, like a sugar pill. There will be 264 people in each active medication condition (1320 total) and 40 people in each placebo condition (200 total). Thus, you have an 87 % chance to be enrolled in an active medication condition and 13 % chance to be enrolled in a placebo medication condition.

If you are assigned to the patch treatment group, you will wear a 21-mg nicotine patch for 4 weeks after quitting smoking, then a 14-mg patch for weeks 5 and 6, and then a 7-mg patch for weeks 7 and 8. If you are using the lozenge (2-mg or 4-mg) you will use them for up to 12 weeks. You will use one lozenge every 1-2 hours during the first six weeks of treatment, using a minimum of 9 lozenges/day. Then you will decrease lozenge use to one lozenge every 2-4 hours during weeks 7-9, and then to one lozenge every 4-8 hours for weeks 10-12. If you are using bupropion SR, you will take one 150 mg pill per day for three days followed by two 150 mg pills per day for 8½ weeks.

Neither you, nor your study personnel, nor any other person directly involved with you during the study, will know what dose of study medication you are taking or whether you are taking a placebo. In an emergency, if a medical doctor needs to know what kind of study medication you have been taking so that you can get appropriate medical care, this information will be opened to determine which treatment you have been assigned to.

Follow-up phone calls will occur at weeks 12, 18, 24, 30, 42 & 52 to inquire about your tobacco use, alcohol use, withdrawal symptoms you have had, and how you feel about smoking and quitting. You will also be asked about risk of suicide during the week 12 call. These calls will take about 10-15 minutes.

Second and third years of the study: If you are not smoking at one year, you will continue in the follow up long-term portion of this study and will be scheduled for the

one-year post-quit in-person assessments (Visit 10/Year 1). If you are one of the first 540 people who have relapsed at one year, you will continue in the follow up long-term portion of this study and will be scheduled for the one-year post-quit in-person assessments (Visit 10). If you are not one of the first 540 people who have relapsed by year one, your participation will conclude at that time.

All subjects continuing in the long-term outcomes portion of this study will attend an in-person clinic visit at Years 1, 2, and 3 (12, 24, and 36 months) post-quit attempt. During these visits, you will repeat many of the baseline assessments, including information to determine smoking status, the nature of any relapse (where you were and who you were with, stressors, alcohol use, and so on), use of any new quit aids, any new quit attempts, and information on how you feel. Most of the questionnaires from Visit 1 will be given again, as well as measurement of your weight, vital signs, waist size, and carbon monoxide.

The **one-year clinic visit** (months 12) will last two days, about two hours per day. The first day will involve questionnaires and interviews about nicotine dependence, personality, how you are feeling, any stress you have, social support (family and friends), your exercise and what you eat. The second day will involve more heart measures including the ultrasound brachial artery reactivity test and a 12-hour fasting blood sample (about 5 tablespoons) for repeat testing like the tests that were performed at baseline. The ultrasound test was done at Visit 2 during the first part of this study. You will be asked to again carry a pedometer, to measure the distance you walk, for one week to provide a measure of your activity. You will also be asked to carry the Electronic Diary again but only for one week.

The **two-year clinic visit** (months 24) will last one day and take about two hours. You will complete the same questionnaires and interviews. You will be asked to carry the pedometer again for one week to measure your activity. No blood will be drawn at this visit.

The **three-year in-person clinic visit** (month 36) will again last two days. On the first day you will complete the same questionnaires and interviews as in the last two visits. The second day will involve more health measures including the ultrasound carotid intima-medial thickness test, the exercise test, and a blood sample. The blood sample will be drawn after not eating for 12 hours. These tests are the same as those done at Visit 2 during the first part of this study. You will again be asked to carry a pedometer for one week to provide a measure of your activity.

Follow-up phone calls will occur at months 18 and 30, after your quit attempt, to ask about your tobacco use, any withdrawal symptoms you may have had, how you feel about smoking and quitting, and your alcohol use. In total, this telephone call is estimated to take between 20 and 30 minutes.

Are there any risks?

Risks associated with taking the study medications are minimal.

Bupropion:
The most common adverse experiences associated with bupropion SR are headache, dry mouth, nausea, dizziness, and insomnia. Rarely, people have experienced seizures while taking bupropion SR. The incidence of seizure is 1 per 1,000 people taking bupropion. People with a history of seizures, a history of a seizure disorder such as epilepsy, currently taking Wellbutrin or any other medication containing bupropion, with a current or previous diagnosis of bulimia or anorexia nervosa, or currently taking a monoamine oxidase (MAO) inhibitor medication should not take bupropion or participate in this study. While on bupropion, participants should not abruptly discontinue the use of alcohol or sedatives (such as benzodiazepines) or take more than the recommended dose of bupropion because it may increase the risk of seizures. There is also the possibility (1 to 3 per 1000 people taking bupropion) of experiencing an allergic reaction resulting in skin rash, hives, swelling, and itchiness. In severe instances, an anaphylactic (whole body) reaction may occur resulting in chest pain, shortness of breath and difficulty breathing that requires medical treatment and may be life threatening. Bupropion is also associated with a slight risk for hypertension (high blood pressure) in some people particularly those taking both bupropion and nicotine replacement therapy. Your blood pressure will be monitored during the time you are taking study medications.

Bupropion and Suicidality: Patients with depression who are treated with antidepressants such as bupropion should be observed closely for clinical worsening of their depression and for suicidality especially at the beginning of drug treatment or when the dose changes, either increasing or decreasing. Patients with depression who are on bupropion and their families should be encouraged to be alert to the emergence of anxiety, agitation, panic attacks, insomnia, irritability, hostility, impulsivity, akathisia (restlessness, inability to sit still), hypomania, mania, worsening of depression, and suicidal ideation, especially early during treatment with bupropion. Such symptoms should be reported to the study physician or staff especially if they are severe, abrupt in onset, or were not part of the patient's prior depressive symptoms.

Nicotine Patch:
The most common adverse experiences associated with the nicotine skin patch are diarrhea, indigestion, nausea and vomiting, dry mouth, muscle and joint pain, sleeplessness, and abnormal dreams. Side effects associated with wearing an adhesive patch include skin rash, redness, itching or irritation of the skin. In most cases, these side effects have been mild to moderate in intensity and resolve once the patch is removed.

Nicotine Lozenge:
The most likely side effects associated with the nicotine lozenge are heartburn, hiccup, nausea, upper airway infections, coughing and sore throat.

Placebo:
There is no guarantee that study medications will lessen nicotine withdrawal symptoms, and, those participants who receive the placebo medication conditions are unlikely to experience lessened withdrawal symptoms. These nicotine withdrawal symptoms include cravings for cigarettes, irritability, increase in appetite, anxiety, depressed mood, difficulty concentrating, and sleep disturbance.

It is important to discuss with the study staff all medication that you are taking, both prescription and over the counter. Monoamine oxidase inhibitors may be harmful if taken at the same time as study medication.

Aside from medication, it is also required that during this study you avoid any tobacco products other than cigarettes. You should also avoid excessive drinking of alcohol while participating in this study. If you are using any of these substances, it is important that you inform the study staff.

Exercise testing**:**
Exercise testing is routinely done in patients with heart disease but it has some risks. During the exercise test, you may notice fatigue, chest discomfort, or shortness of breath. These are signs that may indicate that the test needs to be stopped. Other possible side effects of the exercise test are abnormal blood pressure, fainting, abnormal heart beats (too slow, too fast or ineffective), and very rarely a heart attack. About 1 in every 10,000 patients with heart disease dies during an exercise test. Prolonged chest pain or serious heart rhythm problems happen in about 4 of every 10,000 patients. Up to 2% of patients with heart failure having an exercise test have had a serious abnormal heart rhythm, but none caused immediate death. If you experience any of the above side effects, you will be asked to stop the test. Personnel of the UW Hospital - Outpatient Cardiac Rehabilitation Program, trained to manage such side effects, will supervise your exercise test.

Ultrasounds:
Brachial and carotid artery ultrasounds are safe, noninvasive, and not uncomfortable. There are no known adverse physiologic effects related to diagnostic medical ultrasound. Inflation of a forearm blood pressure tourniquet has not been reported to be uncomfortable in several studies reported in the literature or in our experience. The test lasts for less than 5 minutes. You may become tired or have a stiff neck during the ultrasound of the neck.

Possible discovery of findings related to medical imaging
You will be informed of all findings of clinical significance that may be revealed during the imaging procedure.

Blood drawing:
Blood drawing is associated with temporary pain at the needle insertion site. It also can be associated with bruising, fainting, or, in rare circumstances, a skin infection.

Computer task:
In this task, you will view a series of slides presented on a computer monitor. Some of the images you will view may be somewhat upsetting. Some people get mildly distressed but in most people the feeling passes quickly. If at any time you become too disturbed by the pictures, please stop the task and let a staff person know about your discomfort.

Questionnaires:
The topics discussed in interviews or in questionnaires may cause temporary embarrassment or emotional discomfort for some people. If this happens to you, please let study personnel know about your discomfort. You may elect not to answer any questions that make you uncomfortable.

Quitting Smoking:
Persons who quit smoking may experience a number of unpleasant symptoms as part of the nicotine withdrawal syndrome. These symptoms may include the following: anger, frustration, irritability, craving for nicotine, anxiety, difficulty concentrating, restlessness, decreased heart rate, increased appetite or weight gain. In addition, smoking cessation can increase the likelihood of depression is some individuals and this is the reason that we assess depression and suicidality during the study. Counseling received in the study may help you cope with symptoms such as these. Before you begin taking the study drug, and at several other times during the study, you will be asked some questions about depression. If your answers show that you are dangerously depressed or suicidal, you will not be allowed to stay in the study. Instead, you will be referred to treatment resources that can provide help for your depression. You should promptly report any problems that develop during the study. You will be told of any changes in the way the study will be done and any new risks to which you may be exposed.

Are there any benefits to me?

By participating in this study, you may receive help in quitting smoking. The study medications bupropion, nicotine patch, and nicotine lozenge, as well as smoking cessation counseling may help you stop smoking although this cannot be guaranteed. If you are assigned to the placebo group, no benefit is expected. You will also receive the results of certain laboratory tests at no cost to you. The information gathered during this study may be of future benefit to other people who wish to stop smoking.

Are there any alternatives to participation in this study?

Study staff will discuss risks and benefits of other treatments to help you stop cigarette smoking before you decide to participate in this study. Other treatments include counseling alone, or counseling in combination with either nicotine replacement therapy (such as nicotine patches, aerosols, or gum), or with an approved medication called bupropion.

Will there be compensation for injury resulting from this research?

In the event you are physically injured as a result of participating in this research, emergency care will be available. You will, however, be responsible for the charge for the emergency care. There is no commitment to provide any compensation for research related injury. You should realize, however, that you have not released this institution from liability for negligence. Please contact the investigator, Dr. Timothy Baker, at 608-262-8673 if you are injured or for further information.

Are there any costs?

The study medication (bupropion, nicotine patch, nicotine lozenge) and all clinic visits, counseling and tests related to the study will be provided at no cost to you. All other costs related to your medical care will be your responsibility.

Will I be paid for participating?

You will be reimbursed for your participation in the study based on a pro-rated scale depending on the number of study visits completed. There is a maximum of $285 for completion of all visits during the first year of the study. This includes $50 for Visits 1 and 2, $5 each for Visits 3-8, $40 for participating in the genetic substudy (optional), $40 for Visit 9 (month six), $100 for Visit 10 (year one) and $25 for the return of the electronic diary. For the long-term outcome portion of this study, there is a maximum of $350. This includes $150 for the year two visit and $200 for the year three visit. This represents a maximum amount of $635 for completing all visits for the three-year study period, participating in the genetic substudy, and the return of the electronic diary. If you are not smoking at the end of one year following your quit attempt, you will continue in the long-term portion of the study and thus be eligible for the study maximum of $635. The first 540 people who have relapsed (smoking) at the end of year one will also continue in the long-term portion of the study. If you relapse (smoking) and are not one of the first 540 people, you will finish your participation at one year and therefore be eligible for a maximum reimbursement of $285.

Who will receive the results of this research?

Study staff will have access to your study records. Authorized representatives from GlaxoSmithKline Inc. (the company that will provide the medication for this study) and the Food and Drug Administration (FDA) may inspect your medical records and information collected during the study to assess compliance with the study protocol. The results of this study may also be used for medical and scientific publications, but you will not be identified personally in any presentations or reports about this research.

The UW-Health Sciences Institutional Review Committee (IRB) may also review your original study records to ensure that the study is being conducted properly. Because of the need to release information to these parties, absolute confidentiality cannot be guaranteed.

We have received a Certificate of Confidentiality from the federal government, which will help us protect your privacy. The Certificate protects against the involuntary release of information about you collected during the course of the study to persons not connected with the study. The researchers involved in this project cannot be forced to disclose your identity or any information about you collected in this study in any legal proceedings at the federal, state, or local level, regardless of whether they are criminal, administrative, or legislative proceedings. However, you or the researcher may choose to voluntarily disclose the protected information under certain circumstances. The Certificate of Confidentiality does not prevent the researchers from disclosing voluntarily, without your consent, information that would identify you as a participant in the research project if required by law (e.g. child abuse, reportable communicable disease, threat of harm to self or others) or as required by federal agencies who may review our records under limited circumstances, (e.g. such as a U.S. Department of Health and Human Services (DHHS) request for information for an audit or program evaluation or an FDA request under the Food, Drug and Cosmetics Act.)

By signing this document, you consent and give approval to such reviews, data storage, and disclosure of your confidential information as mentioned above. Aside from the above-mentioned circumstances, every effort will be made to keep your identity confidential. Finally, you should understand that the principle investigator is not prevented from taking steps, including reporting to authorities, to prevent serious harm to yourself or others.

If I start the study, can I choose not to participate?

Your participation in this study is voluntary. You may choose not to participate or to stop your participation in the study at any time without giving a reason. You will not be penalized and will not lose benefits to which you are otherwise entitled.

The principle investigator may also remove you from the study at any time and for any reason. Based on the assessment of the principle investigator, some of the reasons that you might be removed from the study include, but are not limited to, the following: 1.) to improve your medical care, 2.) if you are not taking study medication as instructed by study staff, 3.) if you are not following instructions of your study staff, 4.) if you are not coming to study visits, 5.) if the study is stopped.

Study staff will tell you if any new information becomes available during the study that may affect your willingness to continue participation in this study.

If you choose to withdraw from the study or if you are asked by your personal doctor to leave the study, you must notify study staff immediately and return all study medication given to you. This includes empty packets and any unused study medication.

If you withdraw or are removed from the study, for any reason, study staff will ask you to complete some of the tests previously mentioned in this document and any other procedures that the principle investigator feels are medically necessary. You may also be asked questions about your experience with the study medication and your smoking status.

What if I have questions?

If I have questions about the research study, please contact Dr. Timothy Baker at 608-262-8673. If you have questions on the rights of research subjects, you may contact the hospital Patient Relations Representative at (608) 263-8009.

Authorization / consent to participate in study.

My signature below indicates that I am 18 years or older and have read all of the information given to me in this document. The information in this document has been explained to me and I have had the opportunity to ask questions. My signature below indicates that I voluntarily agree to participate in the study described in this document. I will receive a copy of this signed consent.

_______________________________ _____________

Subject's Signature **Date**

I, the undersigned, certify that to the best of my knowledge, the subject signing this consent form has had the study fully and carefully explained by me and the subject has been given the opportunity to ask questions regarding the contents of this consent form and his/her participation in this research study.

_______________________________ _____________

Signature of Person Obtaining Consent **Date**

FOR WOMEN ONLY:

VOLUNTARY STATEMENT OF INTENT TO AVOID PREGNANCY

I, ____________________ (print name) agree to attempt to avoid pregnancy while I am participating in this study. I will continue to employ medically acceptable means of contraception that have been approved by study staff. These methods include IUD, oral contraceptive, barrier methods, or abstinence. I will immediately contact Dr. Baker if pregnancy is suspected. I am aware that I may decline to sign this statement and my refusal to sign will have no effect on my further treatment; however, I cannot participate in this research study.

______________________________ ______________

Subject's Signature **Date**
